# Single-cell analysis reveals an Angpt4-initiated EPDC-EC-CM cellular coordination cascade during heart regeneration

**DOI:** 10.1093/procel/pwac010

**Published:** 2022-05-18

**Authors:** Zekai Wu, Yuan Shi, Yueli Cui, Xin Xing, Liya Zhang, Da Liu, Yutian Zhang, Ji Dong, Li Jin, Meijun Pang, Rui-Ping Xiao, Zuoyan Zhu, Jing-Wei Xiong, Xiangjun Tong, Yan Zhang, Shiqiang Wang, Fuchou Tang, Bo Zhang

**Affiliations:** Key Laboratory of Cell Proliferation and Differentiation of the Ministry of Education, Peking University Genome Editing Research Center, College of Life Sciences, Peking University, Beijing 100871, China; Key Laboratory of Cell Proliferation and Differentiation of the Ministry of Education, Peking University Genome Editing Research Center, College of Life Sciences, Peking University, Beijing 100871, China; Key Laboratory of Cell Proliferation and Differentiation of the Ministry of Education, Peking University Genome Editing Research Center, College of Life Sciences, Peking University, Beijing 100871, China; Beijing Advanced Innovation Center for Genomics (ICG), College of Life Sciences, Peking University, Beijing 100871, China; State Key Laboratory of Membrane Biology, College of Life Sciences, Peking University, Beijing 100871, China; Key Laboratory of Cell Proliferation and Differentiation of the Ministry of Education, Peking University Genome Editing Research Center, College of Life Sciences, Peking University, Beijing 100871, China; Key Laboratory of Cell Proliferation and Differentiation of the Ministry of Education, Peking University Genome Editing Research Center, College of Life Sciences, Peking University, Beijing 100871, China; Key Laboratory of Cell Proliferation and Differentiation of the Ministry of Education, Peking University Genome Editing Research Center, College of Life Sciences, Peking University, Beijing 100871, China; Key Laboratory of Cell Proliferation and Differentiation of the Ministry of Education, Peking University Genome Editing Research Center, College of Life Sciences, Peking University, Beijing 100871, China; Beijing Advanced Innovation Center for Genomics (ICG), College of Life Sciences, Peking University, Beijing 100871, China; State Key Laboratory of Membrane Biology, Institute of Molecular Medicine, College of Future Technology, Peking University, Beijing 100871, China; Institute of Molecular Medicine, College of Future Technology, Peking University, Beijing 100871, China; State Key Laboratory of Membrane Biology, Institute of Molecular Medicine, College of Future Technology, Peking University, Beijing 100871, China; Key Laboratory of Cell Proliferation and Differentiation of the Ministry of Education, Peking University Genome Editing Research Center, College of Life Sciences, Peking University, Beijing 100871, China; Institute of Molecular Medicine, College of Future Technology, Peking University, Beijing 100871, China; Key Laboratory of Cell Proliferation and Differentiation of the Ministry of Education, Peking University Genome Editing Research Center, College of Life Sciences, Peking University, Beijing 100871, China; State Key Laboratory of Membrane Biology, Institute of Molecular Medicine, College of Future Technology, Peking University, Beijing 100871, China; Institute of Cardiovascular Sciences and Key Laboratory of Molecular Cardiovascular Sciences, School of Basic Medical Sciences, Ministry of Education, Peking University Health Science Center, Beijing 100191, China; State Key Laboratory of Membrane Biology, College of Life Sciences, Peking University, Beijing 100871, China; Key Laboratory of Cell Proliferation and Differentiation of the Ministry of Education, Peking University Genome Editing Research Center, College of Life Sciences, Peking University, Beijing 100871, China; Beijing Advanced Innovation Center for Genomics (ICG), College of Life Sciences, Peking University, Beijing 100871, China; Key Laboratory of Cell Proliferation and Differentiation of the Ministry of Education, Peking University Genome Editing Research Center, College of Life Sciences, Peking University, Beijing 100871, China

**Keywords:** scRNA-seq, zebrafish, heart regeneration, Angpt4, EPDC

## Abstract

Mammals exhibit limited heart regeneration ability, which can lead to heart failure after myocardial infarction. In contrast, zebrafish exhibit remarkable cardiac regeneration capacity. Several cell types and signaling pathways have been reported to participate in this process. However, a comprehensive analysis of how different cells and signals interact and coordinate to regulate cardiac regeneration is unavailable. We collected major cardiac cell types from zebrafish and performed high-precision single-cell transcriptome analyses during both development and post-injury regeneration. We revealed the cellular heterogeneity as well as the molecular progress of cardiomyocytes during these processes, and identified a subtype of atrial cardiomyocyte exhibiting a stem-like state which may transdifferentiate into ventricular cardiomyocytes during regeneration. Furthermore, we identified a regeneration-induced cell (RIC) population in the epicardium-derived cells (EPDC), and demonstrated Angiopoietin 4 (Angpt4) as a specific regulator of heart regeneration. *angpt4* expression is specifically and transiently activated in RIC, which initiates a signaling cascade from EPDC to endocardium through the Tie2-MAPK pathway, and further induces activation of *cathepsin K* in cardiomyocytes through RA signaling. Loss of *angpt4* leads to defects in scar tissue resolution and cardiomyocyte proliferation, while overexpression of *angpt4* accelerates regeneration. Furthermore, we found that ANGPT4 could enhance proliferation of neonatal rat cardiomyocytes, and promote cardiac repair in mice after myocardial infarction, indicating that the function of Angpt4 is conserved in mammals. Our study provides a mechanistic understanding of heart regeneration at single-cell precision, identifies Angpt4 as a key regulator of cardiomyocyte proliferation and regeneration, and offers a novel therapeutic target for improved recovery after human heart injuries.

## Introduction

Myocardial infarction (MI) leads to heart failure and constitutes an important cause of morbidity and mortality in humans, largely due to the limited capacity for myocardial regeneration (Jessup and Brozena, 2003). Although mammalian hearts are capable of regeneration during the neonatal stage, this ability is quickly lost within 7 days after birth in mice (Porrello et al., 2011). In contrast, lower vertebrates such as zebrafish (*Danio rerio*) can recover completely from various heart injuries through efficient cardiac regeneration, leaving little or no scar tissue, during both the embryonic stage and throughout adulthood (Poss et al., 2002; Chablais et al., 2011; Gonzalez-Rosa et al., 2011; Schnabel et al., 2011; Wang et al., 2011). Past studies have revealed many cellular processes and molecular mechanisms of heart regeneration. Cardiomyocytes (CM) have been found to undergo dedifferentiation and proliferation after cardiac injury, and pre-existing cardiomyocytes were identified as the major cellular source to replenish lost cardiomyocytes (Jopling et al. 2010). Furthermore, atrial cardiomyocytes (CM-A) could transdifferentiate into ventricular cardiomyocytes (CM-V) during heart regeneration in zebrafish larvae (Zhang et al., 2013). Recently, non-cardiomyocytes have also been found to participate in the heart regeneration process (Cao and Poss, 2018; Fernandez et al., 2018). Epicardial cells (EP) comprise the outmost layer of the heart, while endocardial cells (EC) make up the inner lining of the heart, both protecting cardiomyocytes lying between these two layers of cells. In addition, EP and EC also provide important paracrine signals to promote cardiomyocyte proliferation upon cardiac injury (Tahara et al., 2016). Epicardium-derived cells (EPDC) are believed to derive from epicardial cells during development, and are also reported to be involved in the heart regeneration process (Lepilina et al., 2006; Bollini et al., 2014; Vieira et al., 2017). Therefore, comprehensive characterization of the fundamental mechanisms involved in the intrinsic heart regeneration capability in zebrafish, and particularly how different cardiac cell types cooperate together to achieve complete regeneration, could shed light on novel therapeutic strategies to restore human heart function after injury. However, previous research has primarily focused on cardiomyocytes, with few reports involving interactions between cardiomyocytes and non-cardiomyocytes, rarely covering interactions among multiple cell types, especially at the single-cell level (Itou et al., 2012; Zhao et al., 2014; Liu and Zhong, 2017; Honkoop et al., 2019).

A common observation of the regeneration process is the reactivation of developmental programs. Studies in different species have shown that many mechanisms directing heart development are also involved in heart regeneration (Uygur and Lee, 2016). For example, cardiomyocytes reduced sarcomeric structures and dedifferentiated into a more embryonic-like state after cardiac injury (Jopling et al., 2010; Sallin et al., 2015; Honkoop et al., 2019). Non-cardiomyocyte cells also activated embryonic programs upon cardiac injury (Lepilina et al., 2006; Vieira et al., 2017; Fernandez et al., 2018). However, genome-wide transcriptome comparison of major cardiac cell types between cardiac development and regeneration is rarely reported. Thus, a comprehensive comparison between heart development and regeneration at the single-cell level would provide valuable clues to explore not only conserved genetic programs and signaling pathways shared by these two processes, but also regeneration-specific cell populations and factors, especially regeneration-specific secreted proteins/ligands mediating cell-cell interactions, which might provide better therapeutic targets for treatment of human cardiovascular disease with improved positive effects.

Here, to define transcriptomic dynamics as well as signaling cascades and cellular interaction networks during zebrafish cardiac regeneration at the single-cell level, we performed high-precision single-cell RNA sequencing (scRNA-seq) on all major cardiac cell types—CM-A, CM-V, EC, EP, and EPDC—from four different stages after ventricular cardiomyocyte ablation, and compared the results with those from untreated control zebrafish at the same time points. We constructed a continuous molecular trajectory for ventricular cardiomyocytes following ablation and identified a specific group of atrial cardiomyocytes with potential for transdifferentiation into ventricular cardiomyocytes, as well as a specific regeneration-induced EPDC population designated as RIC. By functional screening of genes specifically up-regulated in the RIC, we showed that Angpt4 plays an important role in ensuring efficient cardiomyocyte recovery by initiating a signaling cascade through activation of the Tie2-MAPK signaling pathway and retinoic acid (RA) synthesis in EC followed by up-regulation of RA receptors and collagenase gene *ctsk* in cardiomyocytes and increased cardiomyocyte proliferation. The ability of Angpt4 to promote cardiomyocyte proliferation and cardiac repair are conserved in mammals, as demonstrated in neonatal rat cardiomyocytes and mice. Remarkably, Angpt4 is specifically involved in heart regeneration, and dispensable for zebrafish development. Our work provides a comprehensive molecular profile of zebrafish heart development and regeneration at the single-cell level, identified a regeneration specific factor, and also revealed a coordination network among different cell types essential for the cardiac regeneration process, thus providing novel molecular and cellular insights to promote heart repair in humans.

## Results

### Single-cell transcriptome analyses of zebrafish heart development and regeneration

We took advantage of a *Tg*(*vmhc:mCherry-NTR*; *amhc*:*EGFP*) double transgenic zebrafish to specifically ablate CM-V through metronidazole (MTZ) treatment and tracked both ventricular and atrial cardiomyocytes during heart development and regeneration using the double fluorescence reporters (Zhang et al., 2013). MTZ treatment for 4 h at 3 days post-fertilization (dpf) caused a dramatic loss of mCherry-positive fluorescent signals and significant reduction of ventricular size within 1 day post-treatment (dpt), leading to pericardial edema (Fig. S1A and S1B), consistent with a previous report (Zhang et al., 2013). Subsequently, new mCherry-positive cardiomyocytes arose and fully restored the lost CM-V by 4 dpt, as expected.

**Figure 1.**
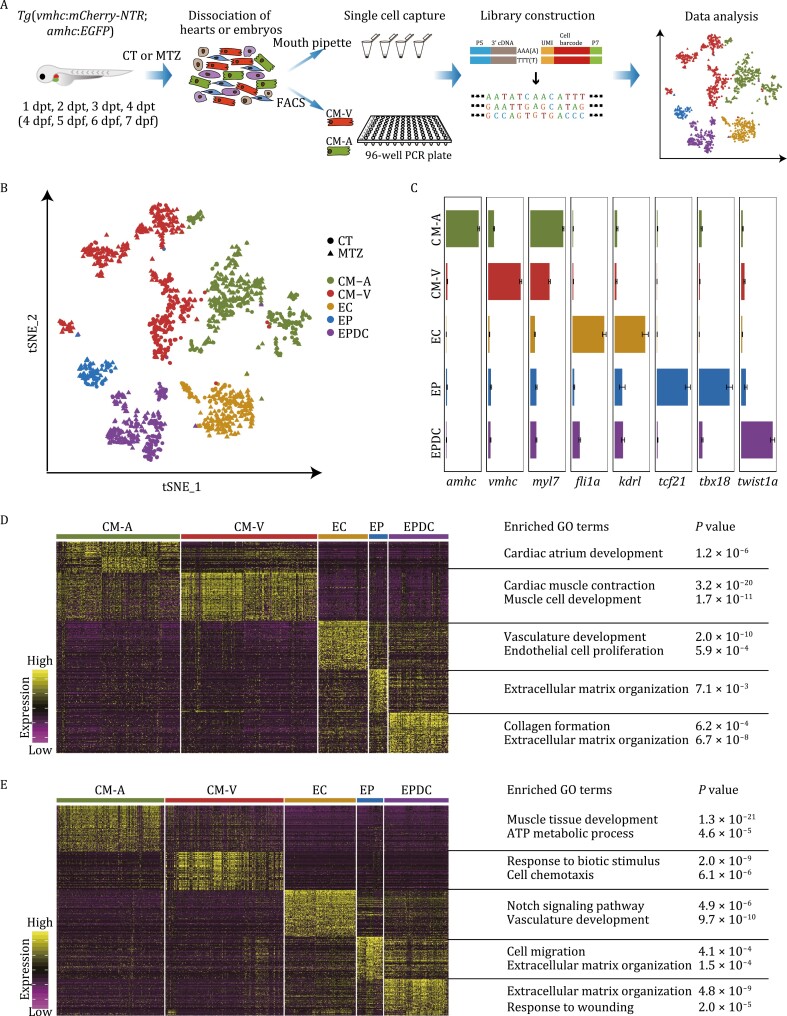
Single-cell RNA sequencing revealed molecular diversity of cells during zebrafish cardiac development and regeneration. (A) Schematic illustration of the workflow for the isolation of single cardiac cells followed by single-cell transcriptome analysis used in this study. CT, untreated control embryos; MTZ, MTZ-treated embryos; dpt, days post-treatment; dpf, days post-fertilization. (B) *t-SNE* analysis results of all captured cardiac populations colored by cluster identity. Single cardiac cells were collected at four stages after ventricular cardiomyocyte ablation induced by MTZ treatment and also from untreated control embryos (CT) of the corresponding developmental stages. CT cell number, 604; MTZ cell number, 977; total cell number, *n* = 1,581. CM-A, atrial cardiomyocyte; CM-V, ventricular cardiomyocyte; EC, endocardial cell; EP, epicardial cell; EPDC, epicardial-derived cell. (C) Bar plots showing the expression of marker genes in each cluster from (B). The expression levels are presented as mean ± s.e.m. (D and E) Heatmap showing the expression levels of DEGs for CT cells (D) and MTZ cells (E) of each cluster. Representative GO terms for each cluster were analyzed by Metascape and shown in the right column.

To characterize the molecular mechanisms underlying heart regeneration at the single-cell level, we obtained 2,336 cardiac cells from four regeneration stages (1 dpt, 2 dpt, 3 dpt, and 4 dpt) after MTZ-induced CM-V ablation, as well as from the same time points for untreated control fish, and performed high-precision scRNA-seq using a modified single-cell tagged reverse transcription (STRT) protocol (Fig. 1A and Table S1) (Picelli et al., 2014; Li et al., 2017, 2019). After stringent filtration, we retained 1581 single-cell transcriptomes for subsequent analyses, with about 2500 genes detected in each cell, on average (Fig. S1C and Fig. S1D). These transcriptomes showed similar levels of housekeeping gene *actb2*, indicating a high consistency of our sequencing processes (Fig. S1E). We first performed t-distributed stochastic neighbor embedding (*t-SNE*) analysis, revealing five major cell clusters (Figs. 1B, S1A, S1B, and S1F). Based on the expression of well-known marker genes, we identified these clusters as CM-A, CM-V, EC, EP, and EPDC (Fig. 1C), encompassing all the major cardiac cell types in zebrafish larvae. These results demonstrate the high quality and reliability of our scRNA-seq data, which provide an important resource for transcriptome-wide dissection of genetic and molecular mechanisms as well as cellular coordination underlying heart development and regeneration.

To reveal differences between normal heart development and the regeneration process, we first explored the transcriptional characteristics of cells from untreated control fish by GO analysis of DEGs (Fig. 1D and Table S2). As expected, both CM-A and CM-V showed enrichment of GO terms related to cardiomyocyte development and function. EC were enriched in GO terms related to vasculature development, and EP were primarily characterized by extracellular matrix (ECM) organization, which has been reported to provide signals for cardiac cell proliferation and maturation (Ieda et al., 2009). EPDC were associated with genes involved in focal adhesion and ECM organization, reflecting their molecular characteristics during development. Next we performed GO analysis on DEGs from MTZ-treated embryos (Fig. 1E and Table S2). Apart from GO terms related to development, we found that MTZ-treated CM-A specifically activated ATP metabolic processes, suggesting an increase in energy metabolism after cardiac injury. Regenerating CM-V specifically induced the response to biotic stimuli after cardiac injury, and genes involved in cell chemotaxis were also up-regulated in these cells, consistent with a previous report that cell migration is necessary for regeneration (Itou et al., 2012). Interestingly, the non-muscle cells (EC, EP, and EPDC) also showed evident responses to cardiomyocyte ablation, as indicated by the specific enrichment of the GO terms “Response to wounding” and “Notch signaling pathway”. These results indicate a coordination and orchestration involving cellular cooperation and signaling cascades during the heart regeneration process.

Transcription factors (TFs) are key and fundamental players in transcription regulation. We next explored differentially expressed TFs in each cell type and revealed novel TFs as well as previously reported TFs involved in the heart regeneration process (Fig. S2). We found that after MTZ treatment, CM-A activated expression of transcription factor genes related to cell mobility, such as *hmga2* and *hmgb3b* (Fig. S2) (Cai et al., 2020; Ren et al., 2020). This is consistent with the requirement for migration of certain CM-A cells towards the ventricular area during heart regeneration for transdifferentiation into CM-V (Zhang et al., 2013). MTZ-treated CM-A also up-regulated *phb*, which is involved in metabolic mitochondrial function and oxidative phosphorylation (Lourenco and Artal-Sanz, 2021). This is consistent with the enrichment of GO terms of ATP metabolic process, and indicates an increase of energy requirement in MTZ-treated CM-A (Fig. 1E). MTZ-treated CM-V activated expression of *pitx2* and *nfe2l2a* (Fig. S2), which have been reported to activate antioxidant response and promote heart repair after cardiac injury in mice (Tao et al., 2016). EC up-regulated *ybx1* after MTZ treatment (Fig. S2), which has been shown to be beneficial for cardiac repair after myocardial infarction in mice (Huang et al., 2019). After MTZ treatment, EP up-regulated *tfa* (Fig. S2), which encodes Transferrin-a, a protein important for heart development and commonly used for diagnosis of human heart failure (Xu et al., 2015; Sierpinski et al., 2021). We also found EP down-regulated expression of *atf3* and *jdp2b* (Fig. S2), whose overexpression leads to cardiac dysfunction, while deficiency preserves cardiac function after cardiac injury in mice (Kalfon et al., 2019). EPDC activated *twist1b* (Fig. S2), encoding a key TF involved in epithelial-to-mesenchymal transition (EMT) (Thiery et al., 2009), after MTZ treatment, suggesting an enhancement of the EMT process after cardiac injury, which is also observed in adult zebrafish and mice after cardiac injury (Lepilina et al., 2006; Duan et al., 2012). Taken together, we provided an informative and valuable resource for interrogation of transcription dynamics of different cell types during zebrafish heart development and regeneration.

### Cardiomyocytes and heart regeneration: trajectory construction of CM

**Figure 2.**
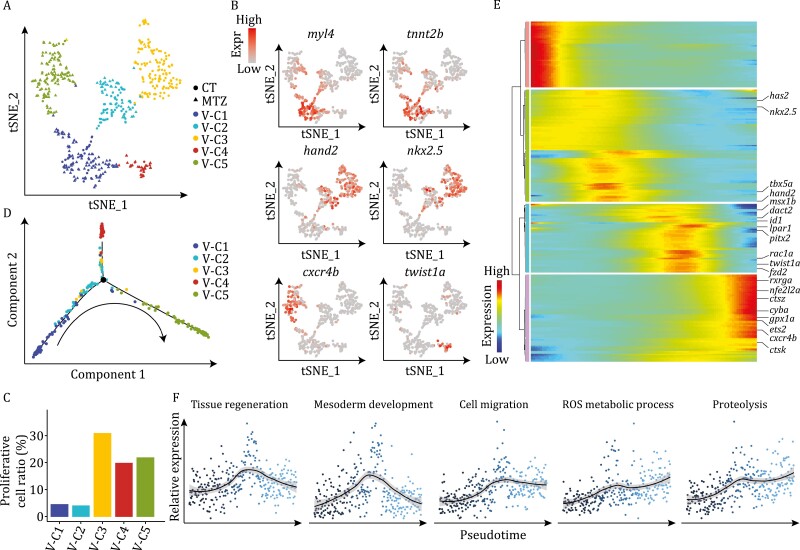
Molecular signatures of ventricular cardiomyocytes during development and regeneration. (A) *t-SNE* analysis results of CM-V from both untreated (CT) and MTZ-treated embryos, showing classification of five populations. Each dot represents a cell, and are colored according to the clusters and shaped by sample conditions. *n* = 510 cells. (B) *t-SNE* maps showing the expression patterns of *myl4*, *tnnt2b*, *hand2*, *nkx2.5*, *cxcr4b,* and *twist1a* in CM-V. (C) Cell cycle score analysis of CM-V subpopulations. (D) Pseudotime trajectory of the MTZ-treated CM-V colored by clusters identified in (A). Arrow indicates the order of pseudotime. (E) Heatmap visualization of changes in expression of representative genes following pseudotime of MTZ-treated CM-V. (F) Pseudotemporal expression patterns of different gene ontologies. Average expression levels smoothed using LOESS are shown.

CM-V are the primary target cells destroyed upon MTZ treatment, and thus are the major cell type in need of restoration, so we first characterized the dynamics and heterogeneity of ventricular cardiomyocytes during the regeneration process. We re-clustered the CM-V cells identified in Fig. 1B, and increased resolution, which resulted in five subtypes (Figs. 2A and S3). Clusters V-C1, V-C2, and V-C3 consisted of ventricular cardiomyocytes from both control and MTZ-treated embryos, of which V-C1 showed specific expression of *myl4* and *tnnt2b*, which are associated with physiological functions of cardiomyocytes such as cardiac muscle contraction, while V-C3 specifically expressed early cardiomyocyte transcription factors such as *hand2* and *nkx2.5* (Fig. 2B). These results indicate that V-C1 represents the population of cardiomyocyte in a well-differentiated and functional state, while V-C3 represents the population of cardiomyocyte precursors. Interestingly, we noticed that V-C2 consisted of cells expressing both early (*hand2* and *nkx2.5*) and mature (*myl4* and *tnnt2b*) cardiomyocyte markers, indicating that this cluster was in an intermediate stage of cardiomyocyte development. These conclusions are further supported by cell cycle analysis results showing that V-C3 has the highest proliferative potential, while V-C1 cells are essentially quiescent (Fig. 2C). Remarkably, clusters V-C4 and V-C5 were almost exclusively composed of MTZ-treated CM-V (Figs. 2A and S3), and both were active in proliferation (Fig. 2C), indicating that they may represent specific intermediate stages during the post-injury regeneration process. Particularly, cells from V-C4 showed specific expression of *twist1a* (Fig. 2B), consistent with previous reports that ECM reorganization is involved in heart regeneration, whereas cells from V-C5 exhibited high expression of *cxcr4b* (Fig. 2B), a cell chemotaxis factor which has been reported to be required for heart regeneration (Itou et al., 2012; Wang et al., 2013a).

We further performed pseudotime analysis using MTZ treated CM-V to reveal the molecular cascades in these cells during regeneration. The result indicates a clear path of sequential stages during the regeneration process, starting from V-C1, then passing through V-C2, V-C3, and V-C4, and eventually reaching V-C5 (Fig. 2D). We subsequently scored the expression signature of genes in specific GO terms to explore their transcriptional changes along the pseudotime trajectory. We found that genes involved in cell migration (e.g., *fzd2*, *rac1a*), mesoderm development (e.g., *dact2*, *lpar1*) and tissue regeneration (e.g., *has2*, *msx1b*) were transiently up-regulated at mid-stages of regeneration (Fig. 2E and 2F), consistent with the previous notion that these biological processes may play important roles in heart regeneration (Wang et al., 2013a; Zhang et al., 2013). We also noticed a transient up-regulation of key early cardiac development transcription factors during the regeneration process, such as *hand2*, *nkx2.5*, and *tbx5a*, indicating that regeneration involves de-differentiation of cardiomyocytes. Furthermore, genes necessary for reactive oxygen species (ROS) metabolism (e.g., *cyba*, *gpx1a*) and proteolysis (e.g., *ctsz*, *ctsk*) were up-regulated mostly at later stages of regeneration, illustrating the involvement of these biological events at the end of regeneration. Genes involved in ROS signaling have been reported to be specifically induced upon cardiac injury and are required for heart regeneration (Han et al., 2014). Proteolysis may be responsible for collagen degradation, which is an essential prerequisite for complete cardiac regeneration (Gamba et al., 2017). These data revealed the molecular cascades orchestrating the highly dynamic transitions of CM-V during the process of cardiac regeneration.

After cardiac injury, CM-A can also contribute to the regeneration process by migration and transdifferentiation into CM-V (Zhang et al., 2013). To reveal the properties of CM-A during heart regeneration, we performed *t-SNE* analysis using both control and MTZ-treated CM-A and identified five clusters (Fig. S4A and S4B). Cells from cluster A-C1 were MTZ-specific, and highly expressed *bmp4*, *bmp5*, and *tgfb3* (Fig. S4A–C), suggesting BMP and TGFβ signaling may be involved in the process of migration and transdifferentiation of CM-A during ventricular cardiomyocyte regeneration. To investigate the cellular events during the transdifferentiation of CM-A to CM-V, we used the RaceID algorithm (StemID) to characterize the degree of cell differentiation (Grun et al., 2016). The results showed that the MTZ-specific A-C1 cluster presented the highest StemID score, suggesting that these cells were in the least differentiated state (Fig. S4D). Not surprisingly, cell cycle analysis revealed that these A-C1 cells were also highly proliferative, indicating that they are likely to be the population contributing to CM-V regeneration by proliferation and transdifferentiation (Fig. S4E).

### EPDC and heart regeneration: a RIC population in the EPDC

Non-muscle cells have also been reported to play essential roles in zebrafish heart regeneration, though precise mechanisms are still largely unknown. In response to cardiac injury, certain epicardial cells undergo EMT, by activating expression of several key regulatory genes, such as *twist1a* and *snai2*, and differentiate into new cell types referred to as EPDC (Cao and Poss, 2018). Depletion of EP could block cardiomyocyte proliferation after ventricular apex resection and reduce the efficiency of heart regeneration (Wang et al., 2015). EC also responds immediately upon cardiac injury and activates the MAPK signaling pathway (Liu and Zhong, 2017). However, interactions between different types of non-muscle cells during heart regeneration and the upstream regulators of MAPK signaling have not been investigated.

We explored the molecular characteristics of non-muscle cardiac cells from both normal and post-injury hearts, and identified five cell clusters by *t-SNE* analysis. Based on the expression of known marker genes, we assigned these clusters as EC, EP, EPDC-C1, EPDC-C2, and proliferating cell (PC) (Fig. S5A–C). The PC cluster highly expressed genes associated with cell proliferation, such as *mki67* (Fig. S5C). Both EPDC-C1 and EPDC-C2 highly expressed genes involved in the EMT process, such as *twist1a* (Fig. S5C), consistent with previous reports showing epicardial cells undergo EMT and give rise to EPDC (Lie-Venema et al., 2007). Interestingly, EPDC-C1 is composed of cells from both MTZ-treated and untreated control embryos (including 77 untreated cells and 22 MTZ-treated cells), while EPDC-C2 is mainly composed of MTZ-treated cells (including nine untreated cells and 124 MTZ-treated cells). EPDC-C2 showed enriched expression of *cxcl12a* (Fig. S5C), which was previously reported to be specifically induced after cardiac injury and essential for heart regeneration (Itou et al., 2012). We termed this regeneration-specific EPDC cluster as the RIC population, and sought to further explore its potential functions in heart regeneration.

### Functional screening identified Angpt4 as a specific and key regulator of heart regeneration that mediates interaction between EPDC and EC cells through activation of MAPK signaling.

To explore potential roles of the RIC population, we performed a functional screen of genes specifically expressed in this population using a fast functional screening strategy based on the clustered regularly interspaced short palindromic repeats (CRISPR)/CRISPR-associated protein (Cas) 9 (CRISPR/Cas9) genome editing system, in which mosaic knockout mutant founder (F_0_) embryos could phenocopy homogenous homozygous mutants (Wu et al., 2018) (Fig. S6A). Among ten genes investigated, *angpt4* mosaic mutant embryos displayed the most striking heart regeneration phenotype after injection of Cas9 protein together with four specific gRNAs into one-cell stage fertilized eggs. Above 40% of the F_0_ embryos failed to regenerate their heart after MTZ-induced ablation of cardiomyocytes (Fig. S6B). As *angpt4* encoded angiopoietin 4 is a secreted factor, this suggests Angpt4 may mediate interactions between EPDC and other cardiac cell types. Thus, we focused on *angpt4* to explore the role of EPDC and potential cellular interactions during heart regeneration.

**Figure 3.**
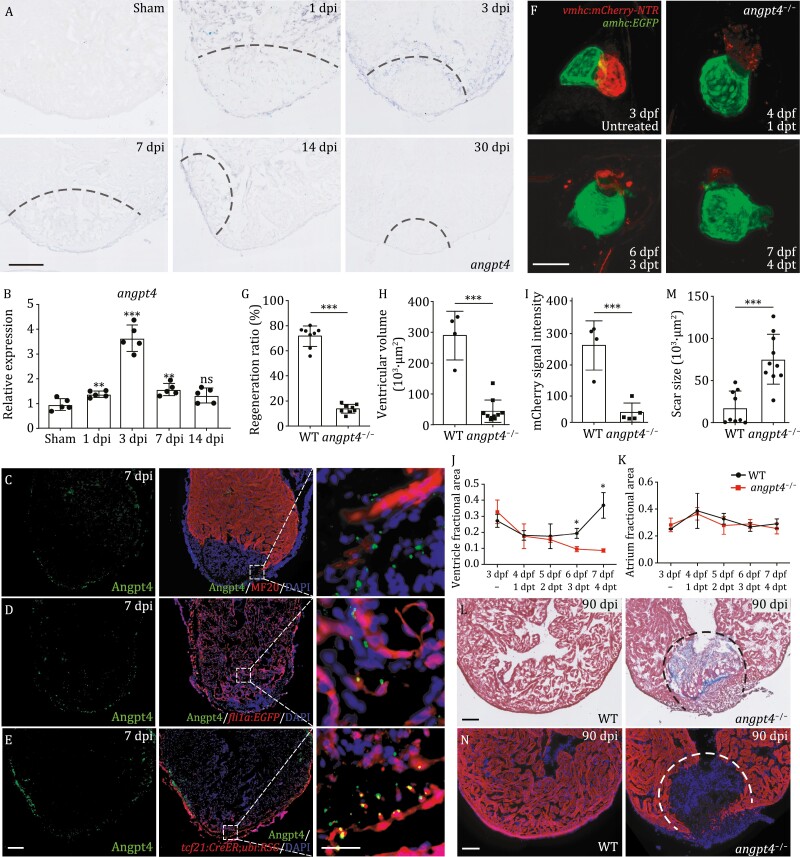
Angpt4 is a key regulator for embryonic and adult heart regeneration. (A) *In situ* hybridization results on tissue cryosections of adult wildtype zebrafish heart at different stages after cryoinjury. The results showed that *angpt4* is transiently up-regulated during heart regeneration. Representative images of three independent replicates are shown. dpi, days post-cryoinjury. Scale bar, 100 μm. (B) qRT-PCR results showing the expression levels of *angpt4* during heart regeneration. Two-tailed Student’s *t*-test, ns, not significant, **P < 0.01, ***P < 0.001. *n* = 5 for each group. Error bar represents standard deviation. (C) Immunostaining results of Angpt4 (green) and MF20 (red; labeling cardiomyocytes) in 7 dpi zebrafish heart cryosections. Right panel shows the enlarged view of the rectangle region. (D) Immunostaining results of Angpt4 (green) and EGFP (red) in *Tg (fli1a:EGFP)* heart cryosections at 7 dpi. Right panel shows the enlarged view of the rectangle region. (E) Immunostaining results of Angpt4 (green) and EGFP (red) in *Tg* (*tcf21:CreER; ubi:loxP-dsRed-STOP-loxP-EGFP*) heart sections at 7 dpi. *ubi:loxP-dsRed-STOP-loxP-EGFP* was abbreviated as *ubi:RSG*. Scale bar, 100 μm. Right panel shows the enlarged view of the rectangle region. Scale bar, 25 μm. (F) Maximum intensity projections of *angpt4* mutant embryos with or without MTZ-treatment under *Tg* (*vmhc:mCherry-NTR; amhc:EGFP*) transgenic background, showing the failure to regenerate ventricular cardiomyocytes (marked by red fluorescent signal). dpf, days post fertilization. dpt, days post-treatment. Scale bar, 50 μm. (G) Statistical analysis results of successful regeneration ratio in MTZ-treated WT and *angpt4* mutant embryos. Two-tailed Student’s *t*-test, ***P < 0.001. *n* = 8 for each group. Error bar represents standard deviation. (H–K) Statistical analysis results of ventricular volume (H), mCherry fluorescence intensity (I), ventricle fractional area (J) and atrium fractional area (K) in MTZ-treated WT and *angpt4* mutant embryos. Fractional area change = (Diastolic area − Systolic area)/Diastolic area. Two-tailed Student’s *t*-test, *P < 0.05, ***P < 0.001. *n* = 4–10 for each group. Error bar represents standard deviation. (L) Representative Masson’s trichrome staining results of adult WT and *angpt4*^−*/*−^ zebrafish heart cryosections prepared at 90 dpi. *angpt4*^−*/*−^ hearts showed larger scar area than WT hearts. Red, muscle cells stained with acid fuchsin; blue, collagen stained with aniline blue. Dotted line indicates scar area. Scale bar, 100 μm. (M) Statistical analysis results of scar size in 90 dpi of WT and *angpt4*^−*/*−^ hearts. Two-tailed Student’s *t*-test, ***P < 0.001. *n* = 9–10 for each group. Error bar represents standard deviation. (N) Immunofluorescence staining results of cardiomyocyte marker MF20 in WT and *angpt4*^−*/*−^ hearts at 90 dpi. Dotted line indicates scar area. Scale bar, 100 μm.

We examined the expression patterns of *angpt4* during larvae and adult zebrafish heart regeneration by *in situ* hybridization. We first examined and compared the expression patterns of *angpt4* in *Tg* (*vmhc*:*mCherry-NTR*; *amhc*:*EGFP*) zebrafish embryos with and without MTZ-induced ventricular cardiomyocyte ablation. Temporally, *angpt4* was significantly up-regulated in the regenerating heart at 1 dpt, after which the mRNA signals gradually decreased, and eventually vanished at 4 dpt, coincident with the timing for the complete recovery of ventricles (Fig. S7A). In contrast, *angpt4* expression was undetectable in the hearts of untreated control embryos (without MTZ treatment) at all the stages investigated in our experiments (Fig. S7A). Spatially, we noticed that after MTZ-treatment, *angpt4* was widely induced to express in the cardiac tissues, including atrium, ventricle, and out flow tract (OFT, also known as bulbus arteriosus), with a clear enrichment at the OFT region (Fig. S7A). To reveal the location and distribution of EPDC cells in zebrafish embryos, we analyzed our scRNA-seq data and found *fn1a* (*fibronectin 1a*), encoding ECM component fibronectin, was highly enriched in the EPDC population during normal development (Fig. S7B). Interestingly, *in situ* hybridization result showed that *fn1a* transcripts were also enriched at the OFT region in zebrafish embryos, comparable to the distribution of *angpt4* transcripts (Fig. S7C). These data suggest EPDC mainly locate at the OFT region in larvae zebrafish, and support our scRNA-seq data that *angpt4* was mainly expressed in EPDC after cardiac injury in zebrafish larvae. Specific activation of *angpt4* was also observed during adult zebrafish heart regeneration, where *angpt4* was quickly up-regulated at the injury area at as early as 1 day post-cryoinjury (dpi) in the regenerating heart, reached the highest level at 3 dpi, and gradually declined until its transcripts were undetectable at 30 dpi (Fig. 3A). qRT-PCR results revealed a similar expression pattern of *angpt4* during the regeneration process (Fig. 3B). Next, we investigated the localization of Angpt4 protein during zebrafish heart regeneration by co-immunostaining with specific cardiac cell type markers on cryosections. The results showed that the Angpt4 signals appeared specifically at the margin areas of injured hearts, and most of the signals were located in EGFP positive cells from *Tg* (*tcf21*:*CreER*; *ubi*:*loxP-dsRed-STOP-loxP-EGFP*) zebrafish heart sections of 7 dpi fish (Fig. 3C–E). No co-localization was observed in either MF20-labeled cardiomyocytes or endocardial/endothelial cells labeled by *Tg*(*fli1a*:*EGFP*) (Fig. 3C–E). These immunostaining results further confirmed our scRNA-seq data showing that *angpt4* was induced in EPDC during zebrafish heart regeneration.

To confirm the essential role of *angpt4* in heart regeneration, we generated a stable mutant line bearing a 2-bp deletion in exon 1 of zebrafish *angpt4* locus (Fig. S8A and S8B). *angpt4* homozygous mutant embryos showed no apparent defects in development (Fig. S8C), adult mutants are both viable and fertile, and showed normal heart morphology (Fig. S8D and S8E), indicating that *angpt4* is dispensable for cardiac development and maturation in zebrafish. In contrast to the dispensable role of *angpt4* in development, regeneration following MTZ-induced ventricular injury was largely abolished in *angpt4* mutant embryos, although the atrium remained morphologically normal (Figs. 3F, 3G and S1B). Accordingly, ventricular functions were also severely impaired in the MTZ-treated *angpt4* mutants, as measured by ventricular volume, mCherry fluorescence intensity, and fractional area change at 4 dpt, while atrial function remained normal (Fig. 3H–K). Furthermore, we examined whether *angpt4* is necessary for adult zebrafish regeneration. Masson’s trichrome staining showed that adult heart regeneration was also impaired in *angpt4* mutants, with significantly larger scars compared to WT fish (Fig. 3L and 3M). MF20 immunostaining results also showed *angpt4* mutants failed to recover injured cardiomyocytes (Fig. 3N). These results demonstrated that Angpt4 is specifically required for heart regeneration processes in both embryonic and adult zebrafish.

**Figure 4.**
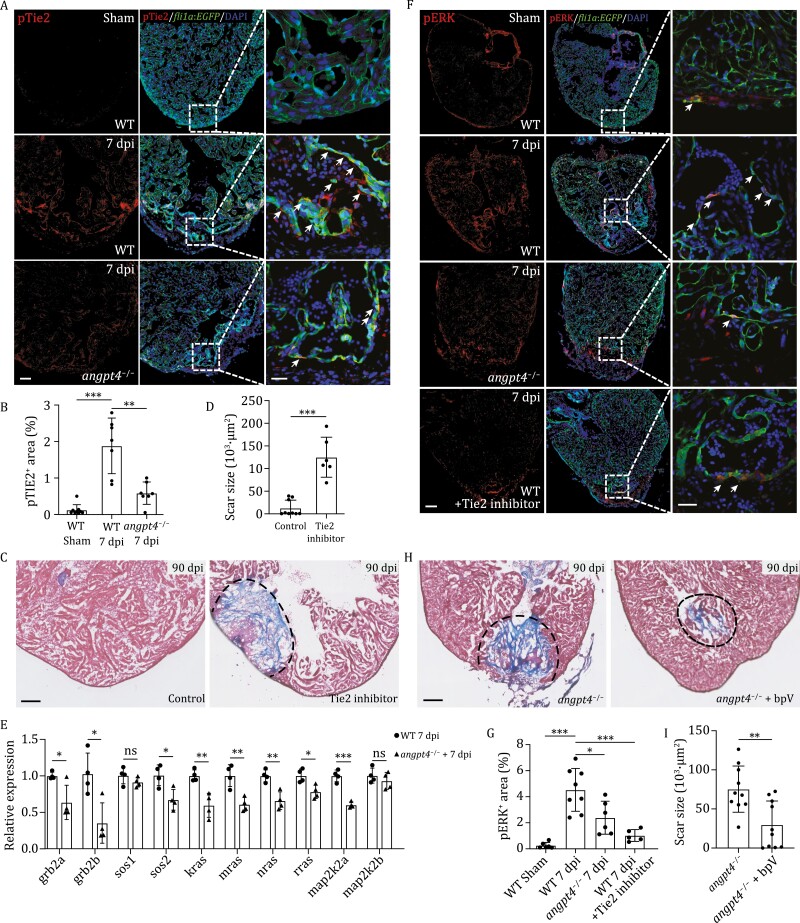
Angpt4 regulates heart regeneration by activating endocardial Tie2-MAPK signaling pathway. (A) Immunofluorescence staining results showing the pTie2 (Tyr992) signal in heart sections from WT at 7 days post sham operation, and WT and *angpt4* mutant at 7 dpi. Scale bar, 100 μm. Right panels showed higher magnification views of the rectangle region. Arrows indicate pTie2-positive EC. Representative images of three independent replicates are shown. Red, pTie2; Green, EGFP, representing EC. Scale bar, 25 μm. (B) Statistical analysis results of relative pTie2 signals in 7 dpi of WT and *angpt4*^−*/*−^ hearts. Two-tailed Student’s *t*-test, **P < 0.01, ***P < 0.001. *n* = 7 for each group. Error bar represents standard deviation. (C) Masson’s trichrome staining results of adult zebrafish heart cryosections from WT and Tie2 inhibitor treated WT fish at 90 dpi. Red, muscle cells stained with acid fuchsin; blue, collagen stained with aniline blue. Scale bar, 100 μm. (D) Statistical analysis results of scar size in 90 dpi WT and Tie2 inhibitor treated WT fish. Two-tailed Student’s *t*-test, ***P < 0.001. *n* = 6–10 for each group. Error bar represents standard deviation. (E) qRT-PCR results showing expression level of several components of the MAPK signaling pathway in 7 dpi WT or *angpt4* mutant zebrafish hearts. Two-tailed Student’s *t*-test, ns, not significant, *P < 0.05, **P < 0.01, ***P < 0.001. *n* = 4 for each group. Error bar represents standard deviation. (F) Immunofluorescence staining of pERK in WT fish at 7 d post sham operation, and WT, *angpt4* mutant, and Tie2 inhibitor treated WT heart sections at 7 dpi under *Tg* (*fli1a:EGFP*) transgenic background. Scale bar, 100 μm. Right panels show higher magnification views of the rectangular region. Arrows indicate pERK signals in EGFP-positive cells. Representative images of three independent replicates are shown. Red, pERK; Green, EGFP, representing EC. Scale bar, 25 μm. (G) Statistical analysis results of relative pERK signals in WT fish at 7 days post sham operation, and WT, *angpt4* mutant, and Tie2 inhibitor treated WT fish at 7 dpi. Two-tailed Student’s *t*-test, *P < 0.05, ***P < 0.001. *n* = 6–8 for each group. Error bar represents standard deviation. (H) Masson’s trichrome staining results of adult zebrafish heart cryosections from *angpt4* mutants and bpV treated *angpt4* mutants at 90 dpi. Red, muscle cells stained with acid fuchsin; blue, collagen stained with aniline blue. Scale bar, 100 μm. (I) Statistical analysis results of scar size at 90 dpi in *angpt4* mutants and bpV treated *angpt4* mutants. Two-tailed Student’s *t*-test, **P < 0.01. *n* = 10 for each group. Error bar represents standard deviation.

Angiopoietins, including ANGPT1, ANGPT2, and ANGPT4, have been reported to act as ligands of the tyrosine kinase receptors TIE1 and TIE2 to activate MAPK signaling pathways during tumor angiogenesis in mammals (Huang et al., 2010). However, the role of Angpt4 has not been reported in zebrafish, and whether Angpt4 could activate MAPK signaling pathway during heart regeneration remains unclear. Interestingly, we found that *tie2* was specifically expressed in EC in zebrafish (Fig. S5D). In order to determine whether Angpt4 regulates zebrafish heart regeneration through Tie2 kinase signaling, we first analyzed the level of phosphorylated Tie2, the activated form of Tie2, by immunostaining in an endocardial/endothelial reporter fish line *Tg* (*fli1a*:*EGFP*). The transgenic zebrafish wildtype for *angpt4* exhibited strong endocardial pTie2 signals near the injury area at 7 dpi. In contrast, pTie2 staining was clearly weaker in the *angpt4* mutants at the same stage (Fig. 4A and 4B). Furthermore, Tie2 kinase inhibitor was injected intraperitoneally daily from 3 dpi to 30 dpi. We found that fibrotic scar tissues in adult fish after Tie2 kinase inhibitor treatment were significantly larger than that in control fish at 90 dpi, which phenocopied the regeneration defects of the *angpt4* mutants (Fig. 4C and 4D). These data suggested that Angpt4 regulates heart regeneration through endocardial Tie2 activation.

Angpt4/Tie2 signals were reported to be able to activate downstream MAPK signaling pathway during lymphatic vessel development (Kesler et al., 2015), and the MAPK signaling pathway has been shown to be activated specifically in endothelial cells during heart regeneration (Liu and Zhong, 2017; Missinato et al., 2018). To further explore whether Angpt4 functions through downstream MAPK signals during zebrafish heart regeneration, we first evaluated the expression of genes in the MAPK signaling pathway. qRT-PCR results showed numerous genes in the MAPK signaling pathway were expressed at lower levels in *angpt4* mutants compared to WT at 7 dpi, indicating MAPK signaling was impaired after *angpt4* loss of function (Fig. 4E). We then evaluated the level of phosphorylated ERK (pERK), an indicator of MAPK pathway activation, by immunostaining in the *Tg*(*fli1a*:*EGFP*) fish. In the hearts of zebrafish wildtype for *angpt4*, pERK was specifically up-regulated in the EGFP-positive EC at 7 dpi in the injury area (Fig. 4F). In contrast, pERK was only weakly detectable in the *angpt4* mutants at the same stage, indicating that ERK phosphorylation is dependent on Angpt4 during cardiac regeneration (Fig. 4F and 4G). Moreover, pERK signal was significantly reduced after Tie2 inhibition by intraperitoneal injection of Tie2 kinase inhibitor daily from 3 dpi to 7 dpi, which again phenocopied *angpt4* mutants (Fig. 4F and 4G). Furthermore, supplementation with bpV, a MAPK agonist, partially rescued the heart regeneration defects in the *angpt4* mutants (Fig. 4H and 4I), confirming that Angpt4 functions through downstream activation of the Tie2-MAPK pathway. Our results revealed a cellular interaction network between EPDC and EC via Angpt4-Tie2-MAPK signaling pathway, which is essential for heart regeneration.

### Angpt4 and endocardial MAPK signaling regulate cardiomyocyte proliferation and scar removal through activation of RA signaling in CM upon heart injury

**Figure 5.**
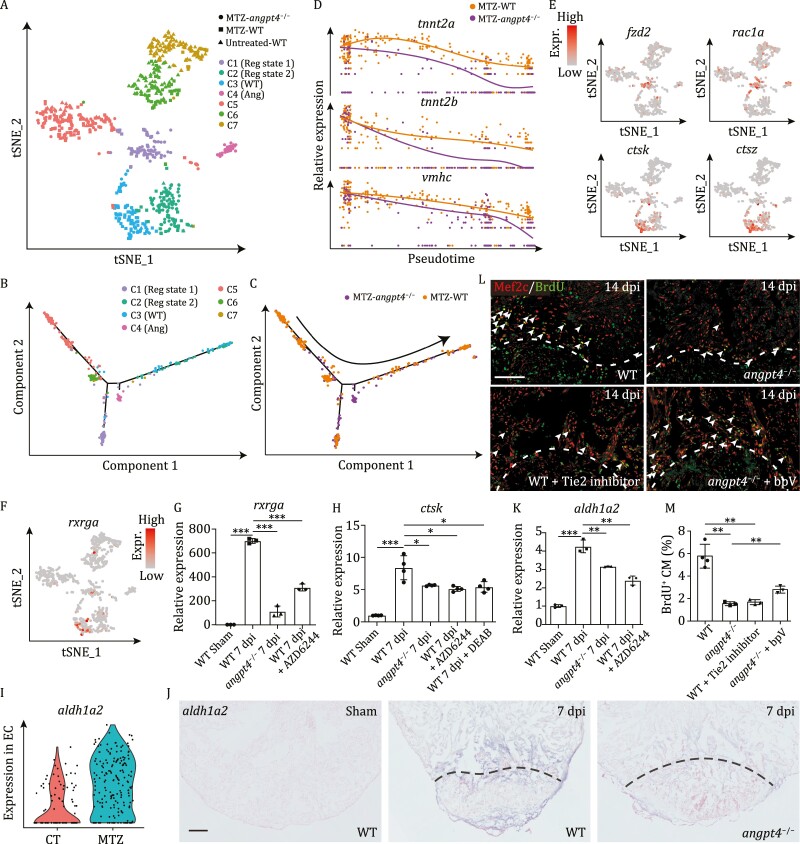
**Single cell transcriptome analysis of ventricular cardiomyocytes from *angpt4* mutant embryos during heart regeneration**. (A) *t-SNE* analysis showing heterogeneity of CM-V from untreated and MTZ-treated wildtype (WT) embryos as well as MTZ-treated *angpt4* mutant embryos. Cells are colored by clusters and shaped by sample conditions. Reg state, regeneration state; WT, WT specific population; Ang, *angpt4* mutant specific population. (B and C) Pseudotime analysis using MTZ-treated wildtype (WT) and *angpt4* mutant CM-V, colored by the clusters identified in panel A (B) and genotype (C), respectively. Arrow indicates the order of pseudotime. (D) The expression pattern of *tnnt2a*, *tnnt2b*, and *vmhc* following pseudotime in MTZ-treated wildtype (WT) embryos and *angpt4* mutants. The expression pattern of WT and *angpt4* mutants were fitted by Monocle2. (E) *t-SNE* maps derived from scRNA-seq data of ventricular cardiomyocytes from WT and *angpt4* mutant embryos based on Fig. 5A, showing expression patterns of *fzd2*, *rac1a*, *ctsk*, and *ctsz*. (F) *t-SNE* maps derived from scRNA-seq data of ventricular cardiomyocytes from WT and *angpt4* mutant embryos based on Fig. 5A, showing expression pattern of *rxrga*. (G) qRT-PCR results showing expression levels of *rxrga* in WT fish at 7 days post sham operation, and WT, *angpt4* mutant, and MAPK signaling inhibitor AZD6244 treated WT zebrafish at 7 dpi. Two-tailed Student’s *t*-test, ***P < 0.001. *n* = 3 for each group. Error bar represents standard deviation. (H) qRT-PCR results showing *ctsk* expression level in WT fish at 7 days post sham operation, and WT, *angpt4* mutant, MAPK signaling inhibitor AZD6244 treated WT, and RA signaling inhibitor DEAB treated WT zebrafish at 7 dpi. Two-tailed Student’s *t*-test, *P < 0.05, ***P < 0.001. *n* = 4 for each group. Error bar represents standard deviation. (I) Violin plot showing *aldh1a2* was up-regulated in EC after MTZ treatment. (J) *In situ* hybridization results showing expression pattern of *aldh1a2* in WT fish at 7 d post sham operation, and WT and *angpt4* mutant at 7 dpi. Representative images of three replicates are shown. Scale bar, 100 μm. (K) qRT-PCR results showing expression levels of *aldh1a2* in WT fish at 7 days post sham operation, and WT, *angpt4* mutant, and MAPK signaling inhibitor AZD6244 treated WT zebrafish at 7 dpi. Two-tailed Student’s *t*-test, **P < 0.01, ***P < 0.001. *n* = 3 for each group. Error bar represents standard deviation. (L) Immunofluorescence staining results showing BrdU signals near the injury site in adult zebrafish heart cryosections from WT, *angpt4* mutant, Tie2 inhibitor treated WT, and bpV treated *angpt4* mutant at 14 dpi. Red, Mef2c; Green, BrdU. Scale bar, 50 μm. (M) Statistical analysis results of BrdU positive cardiomyocytes in (L). Two-tailed Student’s *t*-test, **P < 0.01. *n* = 3–4 for each group. Error bar represents standard deviation.

Cardiomyocyte proliferation and scar removal are two crucial and ultimate events in cardiac regeneration. To further explore the effect of *angpt4* mutation on these processes, we analyzed single-cell transcriptomes of CM-V from MTZ-treated *angpt4* mutant embryos. We combined these data with the scRNA-seq data from the CM-V of both untreated and MTZ-treated wildtype embryos and performed *t-SNE* analysis (Figs. 5A and S9A). Seven clusters were identified, among which clusters C1–C4 are mainly composed of MTZ-treated cells, indicating a regeneration-specific process. To further reveal the impact of *angpt4* mutation on the heart regeneration progress, we constructed a pseudotime trajectory using CM-V from MTZ-treated wildtype and mutant embryos. While the CM-V from MTZ-treated wildtype embryos could fully complete the regeneration process, the MTZ-treated *angpt4*^−/−^ embryos only reached an intermediate stage of the trajectory and then branched away from the wildtype path (Fig. 5B and 5C). Additionally, compared to *angpt4* mutants, MTZ-treated wildtype embryos showed relatively higher expression of mature cardiomyocyte marker genes important for cardiac functions, such as *vmhc*, *tnnt2a*, and *tnnt2b* (Fig. 5D), further confirming the essential function of *angpt4* in regulating heart regeneration.

To reveal the molecular mechanisms underlying impaired heart regeneration in *angpt4* mutants, and how Angpt4-Tie2-MAPK signaling regulates heart regeneration, we evaluated the gene expression signatures in different clusters. We noticed that cluster C1 specifically expressed cell migration genes, such as *rac1a* and *fzd2*, and consisted of cells from both *angpt4*^+/−^ and *angpt4*^−/−^ MTZ-treated embryos, indicating that cell migration is independent of Angpt4 (Fig. 5E). Interestingly, cluster C3 mainly contained cells from the MTZ-treated wildtype embryos, while cluster C4 mainly consisted of cells from MTZ-treated *angpt4*^−/−^ embryos, indicating clear divergence between the wildtype and mutant embryos in the process of heart regeneration. We examined the differentially expressed genes between clusters C3 and C4, and found C3 highly expressed RA signaling receptor *rxrga* (*retinoid x receptor*, *gamma a*) and its downstream gene *ctsk* (*cathepsin K*) (Fig. 5E and 5F), indicating the RA signaling pathway was activated in ventricular cardiomyocytes and this activation depended on Angpt4. This notion is confirmed by qRT-PCR and *in situ* hybridization results, which showed that *rxrga* and *ctsk* were significantly up-regulated at 7 dpi in the wildtype heart, whereas the up-regulation is largely impaired in the *angpt4* mutant heart (Figs. 5G, 5H and S9B). In fact, RA synthesis has been reported to be induced in EC and EP cells after cardiac injury, and activation of RA signal pathway is necessary for cardiomyocyte proliferation (Kikuchi et al., 2011b). In addition, RA signaling has been shown to regulate the expression of collagenase-encoding cathepsin genes during limb regeneration and bone formation (Saneshige et al., 1995; Ju and Kim, 1998). In mice, *Ctsk* has been reported to be induced in cardiomyocytes after cardiac injury, and its deficiency leads to reduced collagen degradation, a process required for scar removal, and impaired cell proliferation after myocardial infarction (Gamba et al., 2017; Fang et al., 2019). RA receptor genes have also been reported to be inducible at the transcriptional level in response to RA signals (Feng et al., 2010). However, the upstream inducer of RA signaling during zebrafish heart regeneration is still unknown. Interestingly, we noticed that *aldh1a2* (*aldehyde dehydrogenase 1 family*, *member A2*), encoding an enzyme responsible for RA synthesis, was up-regulated in the EC cluster after MTZ treatment, as revealed by our scRNA-seq data of WT zebrafish (Fig. 5I). Furthermore, *in situ* hybridization and qRT-PCR results showed that *aldh1a2* was significantly up-regulated in the WT zebrafish heart after cardiac injury, but the up-regulation was clearly impaired in the *angpt4* mutant heart (Fig. 5J and 5K), indicating that Angpt4 is largely responsible for the up-regulation of *aldh1a2*. In addition, qRT-PCR results showed that heart regeneration-induced *aldh1a2* up-regulation was also significantly inhibited by MAPK signaling inhibitor AZD6244 (Fig. 5K), suggesting RA synthesis not only requires Angpt4 but is also dependent on the activation of the MAPK pathway. Together, these data strongly indicate that endocardial MAPK signaling, activated by EPDC-secreted Angpt4, acts as the upstream activator of RA production in EC. Not surprisingly, the up-regulation of *rxrga* and *ctsk* during heart regeneration was also significantly inhibited after treatment with MAPK signaling inhibitor AZD6244 (Fig. 5G and 5H), further confirming that RA signaling activation is downstream to the MAPK pathway. Consistently, the induction of *ctsk* expression after cardiac injury was also reduced by treatment with RA signaling inhibitor DEAB (Fig. 5H), indicating that the expression of *ctsk* is not only dependent on the MAPK pathway, but also positively regulated by RA signaling during heart regeneration. Taken together, our data indicate that RA signaling mediates the interaction between EC and CM cells, and Angpt4 regulates cardiac regeneration through activation of endocardial MAPK signaling in EC and further activation of myocardial *ctsk* expression through RA signaling in CM.

To further reveal the influence of *angpt4* mutation on cardiomyocyte proliferation during heart regeneration, we compared the index of cardiomyocyte proliferation in *angpt4* mutant and wildtype hearts at 14 dpi, by quantifying the percentage of BrdU and Mef2c double positive nuclei. The results showed that cardiomyocyte proliferative ability was significantly reduced in *angpt4* mutants (Fig. 5L and 5M). We also found that inhibition of Tie2 in wildtype zebrafish phenocopied the proliferation defects of *angpt4* mutants, while activating MAPK signaling by bpV treatment could partially rescue the proliferation ability in *angpt4* mutants, indicating that EPDC-derived Angpt4 regulates cardiomyocyte proliferation through activation of Tie2-MAPK pathway in EC during heart regeneration (Fig. 5L and 5M). Taken together, we revealed an important molecular and cellular coordination network during cardiac regeneration, which is initiated by up-regulation of *angpt4* in EPDC, leading to activation of MAPK signaling in EC and further activation of RA signaling, collagen degradation, and cell proliferation in CM, eventually leading to complete recovery of both structure and function of the injured heart.

### ANGPT4 promotes cardiomyocyte proliferation and cardiac repair in fish and mammals

**Figure 6.**
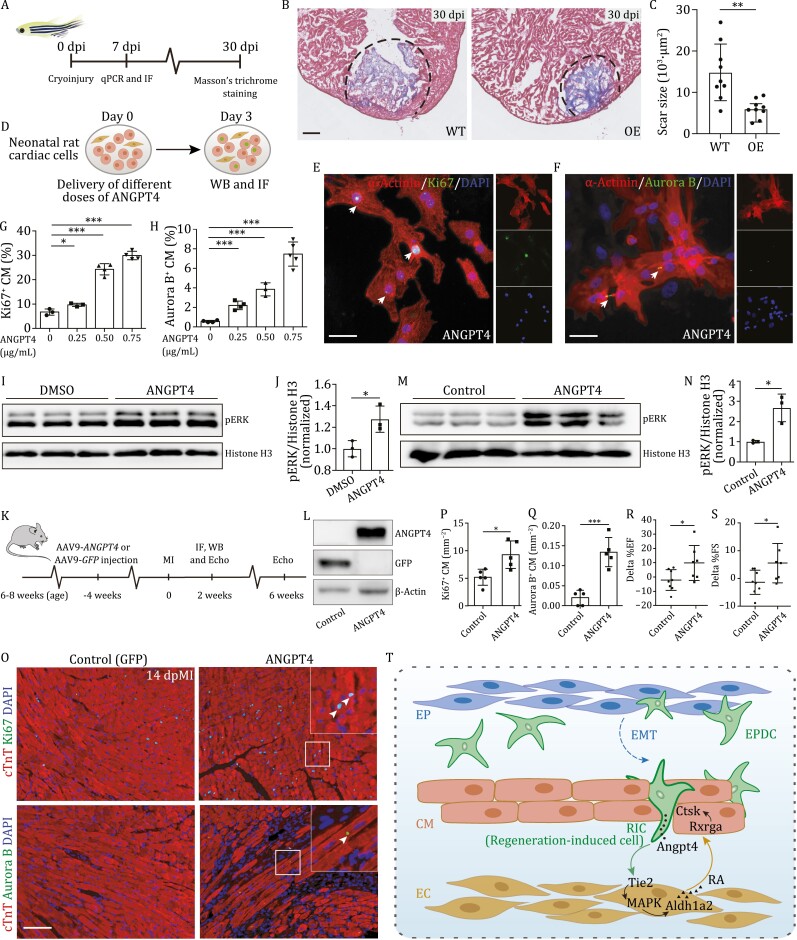
**ANGPT4 promotes cardiac repair *in vitro* and *in vivo***. (A) Experimental design to evaluate heart regeneration potential in *angpt4* overexpressing zebrafish. (B) Masson’s trichrome staining results of adult zebrafish heart sections from wildtype (WT) and *Tg*(*cmlc2:EGFP-angpt4*) (OE) at 30 dpi. Red, muscle cells stained with acid fuchsin; blue, collagen stained with aniline blue. Scale bar, 100 μm. (C) Statistical analysis results of scar size in 30 dpi of WT and *Tg*(*cmlc2:EGFP-angpt4*) (OE) hearts. Two-tailed Student’s *t*-test, **P < 0.01. *n* = 9 for each group. Error bar represents standard deviation. (D) Experimental design for treatment with different doses of recombinant ANGPT4 protein and studying its effect on the expression of cell cycle markers in NRCM. (E and F) Immunofluorescence staining results showing Ki67 (E) and Aurora B (F) signals in NRCM following treatment with 0.75 μg/mL recombinant human ANGPT4 protein. Scale bar, 25 μm. (G and H) Statistical analysis of Ki67 and Aurora B positive NRCM in (E and F). Two-tailed Student’s *t*-test, *P < 0.05; ***P < 0.001. *n* = 3–5 for each group. Error bar represents standard deviation. (I) Western blot of pERK in cultured primary neonatal rat cardiac cells with or without 0.75 μg/mL ANGPT4 protein treatment. (J) Statistical analysis of (I) showing pERK signals were significantly increased in 0.75 μg/mL ANGPT4 treated cardiac cells. Vertical axis shows the pERK-to-Histone H3 ratio, normalized to the control group. Student’s *t*-test, *P < 0.05. *n* = 3 for each group. Error bar represents standard deviation. (K) Experimental timeline for evaluation of cardiomyocyte proliferation and cardiac function after MI in mice injected with AAV9-*GFP* and AAV9-*ANGPT4*. (L) Western blot analysis showing the expression of *GFP* or *ANGPT4* 3 weeks after intravenous AAV9-*GFP* or AAV9-*ANGPT4* injection. (M) Western blot analysis showing the activation of pERK at 2 weeks post MI in AAV9-*ANGPT4* injected mouse hearts. (N) Statistical analysis of (M), showing pERK signals were significantly increased in AAV9-*ANGPT4* injected mice relative to AAV9-*GFP* injected mice. Vertical axis shows the pERK-to-Histone H3 ratios, normalized to the control group. Student’s *t*-test, *P < 0.05. *n* = 3 for each group. Error bar represents standard deviation. (O) Immunofluorescence staining showing Ki67 and Aurora B signals at 2 weeks post MI in cardiomyocytes of AAV9-*GFP* and AAV9-*ANGPT4* injected mice. Scale bar, 100 μm. (P and Q) Statistical analysis of Ki67 and Aurora B positive cardiomyocyte in (M). Two-tailed Student’s *t*-test, *P < 0.05; ***P < 0.001. *n* = 5 for each group. Error bar represents standard deviation. (R and S) Statistical analysis showing differences of cardiac function indexed by EF (%) and FS (%) measured by echocardiography between 2 weeks and 6 weeks post MI. Two-tailed Student’s *t*-test, *P < 0.05. *n* = 7–8 for each group. Error bar represents standard deviation. (T) Model depicting an Angpt4-mediated cellular coordination network among major cardiac cell types during zebrafish heart regeneration. Angpt4 is specifically activated and required for heart regeneration in both zebrafish and mammals. This model illustrates the underlying molecular and cellular mechanisms, where Angpt4 initiates a cellular coordination cascade along an EPDC (RIC)-EC-CM axis by activating MAPK-RA signaling pathways during heart regeneration.

Next, we wanted to determine whether overexpression of *angpt4* could promote heart regeneration in zebrafish, and especially also in mammals. We first constructed a *Tg* (*cmlc2*:*EGFP-angpt4*) transgenic zebrafish line to constitutively express *angpt4* fused with *EGFP* in cardiomyocytes (Fig. 6A). We detected apparent EGFP signal at 3 dpf, and qRT-PCR results showed that *angpt4* was significantly increased in this transgenic line (Fig. S10A and Fig. S10B ). We then performed cryoinjury in adult *Tg* (*cmlc2*:*EGFP-angpt4*) transgenic fish, and examined whether overexpression of *angpt4* could activate downstream Tie2 and MAPK signaling. Immunostaining results revealed a higher level of pTie2 and pERK signal in the *angpt4* overexpressing hearts compared to WT at 7 dpi (Fig. S10C–F). qRT-PCR results also showed many genes in the MAPK signaling pathway were up-regulated in *angpt4* overexpressing hearts (Fig. S10G), as expected. We then analyzed the scar size of WT and *angpt4* overexpressing hearts at 30 dpi. *angpt4* overexpression led to significantly smaller scar tissue compared to wildtype fish, indicating that overexpression of *angpt4* could activate pTie2 and pERK signals and facilitate heart regeneration in zebrafish (Fig. 6B and 6C).

To validate whether the essential role of Angpt4 in regulating heart regeneration is conserved in mammals, we applied recombinant human ANGPT4 protein to isolated and cultured primary neonatal rat cardiac cells, and evaluated NRCM (neonatal rat cardiomyocyte) proliferation potential by immunostaining of Ki67 and Aurora B (Fig. 6D). The results showed that supplementation of ANGPT4 in the culture medium significantly increased the percentage of Ki67-positive and Aurora B-positive cardiomyocytes compared to the DMSO control. Furthermore, the increase in NRCM proliferation ability correlated well with ANGPT4 concentration (Fig. 6E–H). Since we have showed that Angpt4 regulates cardiomyocyte proliferation through Tie2-MAPK signaling in zebrafish, we then examined whether the mechanism is conserved in the rat cardiac cells. We performed immunostaining and Western blot against endothelial marker CD31 and found endothelial cells present in our culture system (Fig. S11A and Fig. S11B). FACS analysis result showed CD31-positive endothelial cells accounted for approximately 3% of total cells (Fig. S11C). Furthermore, Western blot results showed that ANGPT4 protein treatment increased the pERK level in the cultured neonatal rat cardiac cells (Fig. 6I and 6J), suggesting ANGPT4 promotes NRCM proliferation through activation of pERK signaling.

To further verify whether ANGPT4 could also promote *in vivo* cardiac repair in mammals, we intravenously injected AAV9 vectors expressing human *ANGPT4* or *GFP* into adult mice, and verified *ANGPT4* expression in cardiac ventricles after 3 weeks (Fig. 6K and 6L). We found overexpressing *ANGPT4* could activate pERK signaling after MI in mouse hearts (Fig. 6M and 6N). Then, we evaluated cardiomyocyte proliferation ability by immunostaining of Ki67 and Aurora B. The results showed that *ANGPT4* overexpression significantly enhanced mitosis and cytokinesis of cardiomyocytes at 2 weeks post MI (Fig. 6O–Q). Moreover, mice injected with AAV9-*ANGPT4* showed significantly better recovery of cardiac function after myocardial infarction, as assessed by ejection fraction (EF) and fractional shortening (FS), compared with control mice treated with AAV9-*GFP* (Fig. 6R and 6S), suggesting that expression of *ANGPT4 in vivo* could promote cardiac repair in adult mice that would otherwise be non-regenerative after injury.

In summary, we revealed an Angpt4-initiated EPDC-EC-CM cellular collaboration network among three major cardiac cell types to coordinate and regulate heart regeneration in zebrafish (Fig. 6T). More importantly, we showed that the role of Angpt4 is conserved in mammals. The essential and conserved function of Angpt4 as well as the novel mechanisms involving cellular coordination may shed light on treatments of human heart disease.

## Discussion

### Single-cell transcriptome analysis of zebrafish heart during development and regeneration

Zebrafish has remarkable ability to regenerate after cardiac injury both in the larval stage and adulthood. Understanding the mechanisms underlying zebrafish heart regeneration would provide novel insights into treatment of human cardiovascular disease. After cardiac injury, cardiomyocytes acquire a state of partial dedifferentiation and re-activate embryonic programs (Jopling et al., 2010; Sallin et al., 2015; Honkoop et al., 2019). Non-cardiomyocytes, such as EP, EC, and EPDC, are also activated after cardiac injury and induced to express certain embryonic markers (Lepilina et al., 2006; Vieira et al., 2017; Fernandez et al., 2018). These observations raised an interesting question about the similarity of the process and mechanisms between regeneration and development. Not surprisingly, many genes required for development have also been reported to be involved in heart regeneration, e.g., *hand2* and *tbx5a* (Schindler et al., 2014; Grajevskaja et al., 2018). However, a comprehensive comparison between regeneration and development is still lacking, and little is known about the existence and function of regeneration-specific cell populations and factors. Furthermore, interactions between different cardiac cell types during heart regeneration are also poorly investigated.

In this paper, we have profiled all the major cell types of zebrafish embryonic hearts through high-precision single-cell transcriptome analyses before and after MTZ-induced cardiac regeneration. To best of our knowledge, this is the first comprehensive and extensive analysis of all major cardiac cell types throughout the whole regeneration process, and with a detailed comparison with normal development. Our data provide a unique resource and opportunity to understand molecular and cellular mechanisms underlying heart development and regeneration, especially for characterizing regeneration-specific cell populations and factors. Indeed, based on these data, we have identified and explored cell populations (such as RIC) and key factors (such as Angpt4) specific for heart regeneration, crosstalk between signaling pathways (such as MAPK and RA) during heart regeneration processes, as well as an important EPDC-EC-CM interaction cascade ensuring successful heart regeneration (Fig. 6T). Furthermore, we provide evidence showing that the importance of Angpt4 in promoting heart regeneration is well conserved in mammals, providing valuable clues for improving mammalian heart repair and potential novel treatments for MI.

### Injury models to study heart regeneration

Apart from applying genetic ablation in larvae zebrafish to induce heart regeneration, we also adopted cryoinjury to adult zebrafish to introduce cardiac injury for evaluation and comparison of heart regeneration mechanisms. Three cardiac injury models are currently used in zebrafish: ventricular apex amputation, cryoinjury, and genetic ablation (Gonzalez-Rosa et al., 2017). Compared to apex amputation, cryoinjury is considered to be more similar to mammalian myocardial infarction, as the injured tissue is not directly removed from the heart, but rather gradually eliminated through apoptosis. However, neither amputation nor cryoinjury can be applied to larvae or juvenile zebrafish. Therefore, genetic ablation induced by the NTR-MTZ system was established to study heart regeneration in developing zebrafish, and so far is the only method available for this purpose (Zhang et al., 2013). Thus, the genetic ablation system is ideal for performing genetic screens in larval and juvenile zebrafish.

Therefore, we chose the well-established ventricular cardiomyocyte ablation method induced by NTR-MTZ for our scRNA-seq experiments and subsequent genetic screenings for important regulators of heart regeneration in zebrafish larvae, then validated our results as well as investigated the molecular and cellular mechanisms in adult zebrafish heart regeneration using the cryoinjury model, and further extended our study to rodent models. For the heart regeneration study in rodents, we adopted myocardial infarction in adult mice (Cahill et al., 2017). Although no comprehensive evaluation and comparison of different cardiac injury models within zebrafish or between zebrafish and rodents are available in the literature, our consistent findings that Angpt4 is required for heart regeneration either after ventricular cardiomyocyte ablation in larval zebrafish or cryoinjury in adult zebrafish, and that overexpression of *Angpt4* could promote heart regeneration in both zebrafish and rodents, together well indicate that heart regeneration shares common essential mechanisms across different cardiac injury models and regeneration processes between embryonic and adult zebrafish, and is also conserved among different organisms.

### EPDC-derived Angpt4 signaling is essential for heart regeneration

Non-cardiomyocytes, including EC, EP, and EPDC, play important roles during heart regeneration. Compared with EP and EC, EPDC are relatively less investigated. EPDC are believed to derive from epicardial cells during development. Unfortunately, no ideal marker genes have been identified with expression exclusively restricted to the EPDC (Cao and Poss, 2018). Besides, little is known about the differentiation and localization of EPDC in zebrafish larvae. By analyzing our scRNA-seq data, we found that *fn1a*, an ECM encoding gene, expressed highly specifically in the EPDC cluster of zebrafish embryos, and therefore could be used as a marker to represent the EPDC. Our *in situ* hybridization result indicates that *fn1a* is strongly expressed at the OFT area, suggesting that EPDC mainly locate at the OFT region in larval zebrafish. Recently, EPDC were reported to respond to cardiac injury and promote cardiomyogenesis in adult zebrafish (Lepilina et al., 2006; Bollini et al., 2014; Vieira et al., 2017; de Bakker et al., 2021). However, whether EPDC are also involved in larval zebrafish heart regeneration is unclear.

Here, we explored the molecular features of EPDC at the single-cell level during zebrafish embryonic heart development and regeneration, and identified a regeneration-induced EPDC population termed RIC, characterized by specifically up-regulated genes, including *angpt4*. Our results revealed an important role of EPDC during heart regeneration, by promoting cardiomyocyte recovery through activating a signaling cascade initiated by Angpt4 secreted from EPDC and mediated by EC, thus comprising a cellular coordination pathway from EPDC to EC and then to CM. Further studies are needed to investigate the upstream signals and mechanisms for the activation of EPDC after cardiac injury. Single-cell multi-omics sequencing and spatial transcriptomics could also provide valuable clues to further understand the characteristics and mechanisms underlying the zebrafish heart regeneration process, especially in revealing the dynamics and functions of EPDC.

Angiopoietins are a class of secreted factors mainly recognized to be involved in tumor angiogenesis (Davis et al., 1996; Huang et al., 2010; Parmar and Apte, 2021). ANGPT4 has also been reported to be involved in retina angiogenesis in mice (Elamaa et al., 2018). However, the expression and function of angiopoietin family genes are poorly investigated either in zebrafish or during regeneration in any organism. By functional screening of genes in zebrafish specifically induced in EPDC by cardiac injury, we identified *angpt4* as an essential gene for heart regeneration. Our further analysis showed that Angpt4 is not required for zebrafish development, but could be rapidly activated to promote heart regeneration through activation of endocardial MAPK signaling, which then induced the synthesis of RA in EC, and further activated RA receptors and downstream collagenase gene *ctsk* in the regenerating cardiomyocytes.

MAPK signaling pathway has been shown to be activated in EC after cardiac injury, and is required for zebrafish heart regeneration (Liu and Zhong, 2017), though its upstream activators and downstream effectors/signals linked to cardiomyocytes are unclear. In this study, we provide evidence showing that Angpt4 promotes heart regeneration by acting as the upstream inducer of MAPK signaling in response to cardiac injury both in zebrafish and mammals. We further revealed that RA production could be one of the downstream events after MAPK signaling activation in EC. RA synthesis was previously reported to be induced both in EC and EP after zebrafish cardiac injury and activation of RA signaling is necessary for zebrafish heart regeneration, though its upstream signals remain elusive (Kikuchi et al., 2011b). Our work established a link between MAPK and RA signaling pathways in EC, and placed MAPK signaling as the upstream activator of RA synthesis. Though RA and MAPK signal pathways were both reported to be involved in zebrafish heart regeneration, our data first proposes an interaction between these two pathways during this process. Furthermore, our results also revealed *ctsk* was regulated by MAPK and RA signals, thus providing a potential mechanism of how endocardial MAPK pathway and RA signals together regulate cardiomyocyte behavior during regeneration.

Overall, we provide an Angpt4-initiated cellular coordination network illustrating the synergistic interactions involving MAPK and RA pathways along the EPDC-EC-CM axis during cardiac regeneration. Nevertheless, the upstream events activating *angpt4* expression as well as detailed molecular mechanisms coordinating the activity and interaction of these three cell types need further study.

### Potential application of Angpt4 in treatment of human cardiovascular disease

Intriguingly, Angpt4 is not expressed in normal heart and is dispensable for zebrafish development and survival, but is necessary for cardiac regeneration, indicating that it is a key regulator specific to regeneration. We also found that cardiac overexpression of *angpt4* could facilitate heart regeneration in both zebrafish and mammals, while not interfering with survival and development. Remarkably, we found ANGPT4 protein could promote the cell proliferation ability of NRCM in a dose dependent manner, and overexpression of *ANGPT4* could promote mouse cardiomyocyte proliferation and cardiac repair *in vivo*. These data indicate Angpt4 is an ideal candidate to be used for the treatment of human cardiovascular disease.

Importantly, neither deficiency nor cardiac overexpression of *angpt4* caused deleterious effects on the viability of embryonic or adult zebrafish, indicating minimal safety concerns in using Angpt4 as a potential therapeutic target to facilitate cardiac repair in humans. Furthermore, Angpt4 is a secreted factor, therefore it is simple and easy to deliver, and could offer better control in dosage and time windows compared to intracellular proteins such as transcription factors, which are key concerns for medical applications. Nevertheless, we noticed that overexpressing *angpt4* could enhance pERK signal and up-regulate genes in the MAPK signaling pathway during zebrafish heart regeneration, including oncogenic genes *kras* and *nras*. This raises certain concerns about potential safety issues of therapeutic applications of Angpt4. However, as human cardiomyocytes are rarely proliferative in both homeostasis and after injury such as myocardial infarction, up-regulation of these oncogenic genes are more likely to promote the proliferation ability of the cardiomyocytes and benefit recovery of lost cardiomyocytes, rather than cause continuous proliferation leading to tumor formation. Besides, as a secreted protein, Angpt4 induction of the expression of oncogenic genes could be strictly modulated, thus avoiding unwanted deleterious effects. Nevertheless, the application of Angpt4 in the therapy of human cardiac disease needs further validation and careful safety assessment. In fact, Angpt1 has been applied to facilitate skeletal muscle regeneration, angiogenesis and bone repair (Cho et al., 2006; Lee et al., 2008; Youn et al., 2018).

Overall, our study provides a comprehensive analysis of *in vivo* zebrafish heart development and regeneration at single-cell resolution, and provides valuable clues to understand mammalian heart diseases as well as potential mechanisms to activate heart regeneration as a treatment for MI in humans.

## Methods

### Animal husbandry

Zebrafish (Tübingen, TU) were raised at 28.5°C under a 14 h/10 h light/dark cycle in a circulating system and handled according to the regulation of Institutional Animal Care and Use Committee (IACUC) of Peking University. The Peking University IACUC reference number was LSC-ZhangB-2. The following transgenic zebrafish lines were used: *Tg* (*vmhc*:*mCherry*-*NTR*; *amh*c*:EGFP*) (Zhang et al., 2013), *Tg* (*fli1a*:*EGFP*) (Wang et al., 2013b), *Tg* (*tcf21*:*CreER*) (Kikuchi et al., 2011a), *Tg* (*ubi*:*loxP*-*dsRed*-*STOP*-*loxP*-*EGFP*)^*pku372*^, and *Tg* (*cmlc2*:*EGFP*-*angpt4*). The *Tg* (*ubi*:*loxP-dsRed-STOP-loxP-EGFP*)^*pku372*^ transgenic line was generated by using *Tol2*-based transgenesis (Kawakami et al., 2004), after engineering of the pCM206 (pENTR5ʹ_ubi) and Tg (β-actin:loxP-dsRed-STOP-loxP-EGFP) plasmids (Mosimann et al., 2011; Paffett-Lugassy et al., 2017). The two plasmids were kindly provided by C. Geoffrey Burns (Massachusetts General Hospital, Boston, MA, USA). Considering Angpt4 is a secreted factor, we used *cmlc2* promoter to drive expression of *angpt4* in cardiomyocytes, similar to a previous published study (Gemberling et al., 2015). The construct to create *Tg* (*cmlc2*: *­EGFP-angpt4*) was generated by cloning the full length of *angpt4* coding sequence and EGFP, separated by T2A peptide, under the control of *cmlc2* promoter. *Tol2* system was used to generate the transgenic line under TU background. The following four target sites were used to generate *angpt4* mosaic mutant embryos for the fast functional screening of heart regeneration phenotype using the CRISPR/Cas9 system: GGGAACGGTCAAGGGAGACG; CTTACTCTCCAGATGACTGG; CATTTGGCGTAATGCCTGTC; GTTCCACTCGAAGGGAGTAC. Cas9 protein (Novoprotein, E365) was used, and gRNAs were prepared as previously reported (Chang et al., 2013). Among them, the first target site was further used to generate the stable *angpt4* mutant allele bearing the 2-bp deletion.

Mice were maintained in the Laboratory Animal Center (an animal facility accredited by the Association for Assessment and Accreditation of Laboratory Animal Care) at Peking University, Beijing, China. Adult male mice were randomly divided into experimental groups. All procedures involving experimental animals were performed following protocols approved by the Committee for Animal Research of Peking University and conformed to the Guide for the Care and Use of Laboratory Animals.

### MTZ treatment

The *Tg* (*vmhc*:*mcherry-NTR*; *amhc*:*EGFP*) larvae were treated with 5 mmol/L MTZ (Sigma) at 3 dpf for 4 h, as previously described (Zhang et al., 2013). Siblings were treated with 0.2% DMSO as a control. The embryos were washed with water from the circulating system of the fish facility several times after treatment. All the transgenic embryos used were in the heterozygous state. Treated embryos were imaged at 4 dpt, and we calculated the percentage of embryos which successfully recovered mCherry signals as the regeneration ratio of each clutch. Eight clutches each of WT embryos and *angpt4* mutants were quantified.

### Tissue isolation and single-cell preparation

For isolating ventricular and atrial cardiomyocytes, the *Tg* ­(*vmhc*:*mCherry-NTR*; *amh*c*:EGFP*) transgenic embryos were digested with 0.13% trypsin (Sigma), 0.1% collagenase II (GIBCO), and 0.1% collagenase IV (GIBCO) in L15 medium (Sigma) at 37°C for 20 min with pipetting. An equal volume of L15 medium with 0.1% BSA (Sigma) was added to stop digestion. Then the cell suspension was centrifuged at 500 ×*g* for 5 min at 4°C, and the pellet was resuspended in 400 μL of L15 medium with 0.1% BSA. Through BD FACS Aria SORP (Special Order Research Product) sorting, we collected individual mCherry-positive cells (ventricular cardiomyocytes) or EGFP-positive cells (atrial cardiomyocytes) into 96-well plates (Axygen) containing 2 μL lysis buffer with unique cell barcode primers.

To obtain non-muscle cardiac cells, the hearts were isolated by manual dissection of embryos under a dissecting microscope (Leica, M165FC), and then digested as described above. Single cells were randomly picked and transferred into prepared lysis buffer with a mouth pipette.

### Single-cell RNA-seq library construction and sequencing

Single-cell RNA-seq library construction was performed as previously described (Cui et al., 2019; Lu et al., 2019). This modified method was based on the STRT-seq and Smart-seq2 methods. Briefly, the amplified cDNA bearing different cell barcode primers were pooled and purified with 0.8× XP DNA beads (Beckman) twice and then amplified for four cycles with primers with the Illumina index sequence and a biotin modification. After purification with 0.8× XP DNA beads once (Beckman), the DNA was sheared to approximately 300 bp by ultrasonicator (Covaris S2), followed by Dynabeads MyOne Streptavidin C1 beads (Invitrogen) to capture the 3ʹ cDNAs. Thereafter, the library was constructed using a Kapa Hyper Prep Kit (Kapa Biosystems) and the cleaned library was sequenced on an Illumina HiSeq 4000 platform with 150-bp paired-end read length.

### Single-cell RNA-seq data processing

Initially, for single-cell RNA-seq raw data, the cell barcode and unique molecular identifiers (UMI) sequence were extracted from read2 and added to the corresponding read1 with UMI_Tool (Smith et al., 2017). Then the read1 sequences were processed to filter the low-quality bases (*N* > 10%) and trimmed polyA tail sequence and TSO sequence, and the reads of <37 bp in length were discarded. After that, the clean reads were aligned to the zebrafish GRCz10 genome (Ensembl) with STAR (Dobin et al., 2013). Gene and transcript annotations used were also obtained from the Ensembl Zv10 Release 90 reference. Uniquely mapped reads were counted by feature counts and then grouped by cell barcodes for each cell. Then based on UMI information, we removed duplicate transcripts which had the same UMI sequence, and finally with UMI_Tools, distinct UMIs of each gene were counted as the transcript copy number of that gene in each individual cell.

### Identification of cell types and subtypes

For all the 2,336 sequenced single cells, we log-transformed expression data and retained cells that expressed more than 1000 genes, and only genes expressed in at least three single cells with >1 expression level were considered. We further removed blood cells, leaving 1,581 cells and 24,783 genes for the downstream analysis. We used the Seurat package (v3.2.2) and identified 2,000 highly variable genes, which were used for subsequent principal component analysis (PCA) (Butler et al., 2018). We performed JackStraw analysis to identify significant PCs. Fifteen PCs (PCs 1–15) were used for subsequent *t-SNE* analysis. We then used “FindClusters” function to cluster cells, with a parameter resolution = 0.2.

To further identify subpopulations of each cell type, we performed second-level clustering using Seurat as above.

Differentially expressed genes (DEGs) for different clusters were identified by a standard area under curve (AUC) analysis implemented in Seurat. Only DEGs with a power > 0.4 and fold change > 1 were retained. Gene Ontology (GO) analysis was performed using Metascape (Zhou et al., 2019).

### Pseudotime analysis

To specifically reveal the CM-V regeneration process and avoid the influence from other cells, we used Monocle2 to perform pseudotime analysis only in the 298 MTZ-treated CM-V based on the above clustering results (Trapnell et al., 2014). We identified variable genes with Monocle2 and used “reduceDimension” function to reduce dimensionalities, with parameters “max_components = 2” and “reduction_method = ‘DDRTree’”. Then we used “orderCells” to place the cells in pseudotime order. “plot_cell_trajectory” was used to display the pseudotime trajectory. We used “plot_pseudotime_heatmap” to display genes which are differentially expressed in different clusters identified by Seurat. We performed GO analysis using Metascape (Zhou et al., 2019). Genes under a certain GO term were downloaded from Gene Ontology resource (Gene Ontology, 2021) and was used for expression scoring analysis. The expression score was the sum of expression values for gene of each given GO term for each single cell.

### StemID analysis

We used the previously published algorithm StemID to calculate “stemness” of a given cell population (Grun et al., 2016). In short, StemID calculates the number of links between clusters, and multiplies this with entropy change, to generate StemID score.

### Cell cycle analysis

Cell cycle analysis was performed as previously reported (Zhong et al., 2018; Cui et al., 2019), where cells in either the G_1_/S or G_2_/M phases are defined as proliferative. Briefly, we used previously defined sets of cell cycle genes, including 43 G_1_/S and 54 G_2_/M genes (Tirosh et al., 2016), to evaluate the cell cycle state of each cell. The average expression level of each gene set in a single cell was defined as the corresponding “cell cycle score” of each cell. A cell is considered proliferative when the score of either of these two gene sets was > 2. Then the percentage of proliferative cells in a cluster is calculated to represent the proliferation potential of this cluster.

### Cryoinjury and histology staining

The ventricular cryoinjury to induce heart regeneration in adult zebrafish was performed as previously described (Gonzalez-Rosa et al., 2011). For histology staining, adult zebrafish hearts were dissected and fixed in 4% PFA for 2 h at room temperature, then transferred into 30% sucrose in PBS for 4 h at 4°C. The hearts were embedded using O.C.T. compound (Coolaber) and rapidly frozen in liquid nitrogen. Tissue samples were stored at −80°C. Masson’s trichrome staining was performed on 10 μm sections as previously reported (Zhang et al., 2013). Myocardium and collagen were stained by acid fuchsin and aniline blue, respectively. Scar size was analyzed using ImageJ (Fiji) software (National Institutes of Health, Bethesda).

### *In situ* hybridization

*In situ* hybridization was performed on zebrafish embryos and cryosections of adult zebrafish hearts, using digoxigenin-labeled RNA probes, as previously described (Chi et al., 2008; Tong et al., 2014). The primers used for *in situ* hybridization are listed in Table S3.

### Immunofluorescence staining

Immunofluorescence staining was performed on cryosections as previously described (Han et al., 2014). The primary antibodies used in this study were: anti-EGFP (Abcam), anti-pERK (Cell Signaling Technology), anti-pTie2 (Tyr992) (Millipore), anti-Mef2c (Abcam), anti-MF20 (DSHB), anti-BrdU (Sigma), anti-α-actinin (Sigma), anti-CD31(Abcam), anti-Angpt4 (Invitrogen), anti cTnT (Abcam), anti-Ki67 (Abcam), and anti-Aurora B (Abcam). The secondary antibodies used in this study were: Alexa Fluor 488 goat anti-mouse IgG (Invitrogen), Alexa Fluor 594 goat anti-rabbit IgG (Invitrogen), Alexa Fluor 647 goat anti-chicken IgY H&L (Abcam). Nikon A1 confocal microscope and Zeiss Axio Scan were used to observe and record the immunostaining images. To quantify the pERK and pTie2 signals, heart area and pERK/pTie2 positive area were analyzed using Surface function in Imaris, then we calculated the ratio of pERK/pTie2 positive area/heart section area.

### qRT-PCR analysis

qRT-PCR were performed as previously reported (Li et al., 2019). Briefly, RNA was isolated using TRIzol reagent (Invitrogen) following the standard protocol, and then used for reverse transcription with 5× All-In-One RT MasterMix (Applied Biological Materials). qRT-PCR were then performed in a Roche LightCycler 96 instrument using EvaGreen 2× qRT-PCR Mastermix (Applied Biological Materials). The primers used for qRT-PCR are listed in Table S3.

### Chemical treatment

Thirty microliters of 2.5 mg/mL BrdU (Sigma) were intrathoracically injected into adult zebrafish daily from 3 to 14 dpi. 30 μL of 50 μmol/L bpV (Selleck), 50 μmol/L Tie2 kinase inhibitor (Selleck), 20 μmol/L AZD6244 (Selleck) or 20 μmol/L DEAB (Sigma) was intrathoracically injected daily from 3 to 7 dpi for immunostaining or qRT-PCR analysis, or from 3 to 14 dpi for cell proliferation analysis, or from 3 to 30 dpi for Masson’s trichrome staining.

Heterozygous *Tg* (*vmhc:mcherry-NTR*; *amhc*:*EGFP*) transgenic zebrafish larvae were treated with 5 mmol/L MTZ (Sigma) at 3 dpf for 4 h, as previously described (Zhang et al., 2013). Siblings were treated with 0.2% DMSO as control. The embryos were washed with fish water from the circulating system of our fish facility several times after treatment.

Adult *Tg* (*tcf21*:*CreER*; *ubi*:*loxP-dsRed-STOP-loxP-EGFP*) transgenic fish were treated with 1 μmol/L 4-OHT (Sigma) every 2 days for a week, as previously reported (Jopling et al., 2010).

### Cardiac functional analysis

Zebrafish embryos were embedded in 1.5% low-melting agarose. Ventricular volume and fractional area change were calculated as previously described (Fink et al., 2009). Fractional area change = (Diastolic area − Systolic area)/Diastolic area. *Tg* (*vmhc*:*mCherry-NTR*; *amhc*:*EGFP*) hearts were visualized under confocal microscopy (Nikon A1), ventricular volume, and total fluorescence intensity were measured using Imaris.

### Neonatal rat cardiac cell culture and recombinant ANGPT4 protein treatment

Neonatal (P0) rat heart cells were isolated by enzymatic disassociation. Briefly, ventricles of P0 rat hearts were separated from atria and cut into pieces in cold Hanks (Sigma) buffer, then dissociated in Hanks buffer containing 1 mg/mL trypsin (Gibco), 0.8 mg/mL collagenase II (worthington) and 3.57 mmol/L NaHCO3, via physical stirring. The collected heart cells were filtered by a cell strainer (100 μm, BD Falcon), and centrifuged and resuspended in DMEM (Sigma) containing 10% fetal bovine serum (Gibco) and 1% penicillin and streptomycin (Macgene). The collected cells were then plated onto 100-mm plastic dishes for 2 h to remove fibroblasts. The supernatant composing mostly of cardiomyocytes and some endothelial cells was collected and plated on 24-well plates at approximately 10^4^ cells per well. Recombinant human Angiopoietin-4 (ANGPT4, R&D system) was added to the cell culture medium at the final concentrations of 0.25 μg/mL, 0.50 μg/mL, and 0.75 μg/mL. After 3 days of Angpt4 treatment, heart cells were fixed with 4% PFA for 15 min for further immunostaining. FACS analysis was performed at the same time point to analyze the percentage of endothelial cells in the *in vitro* culture system, using FITC anti-CD31 (Abcam).

### Western blot analysis

Western blot analysis was performed as previously reported (Song et al., 2021). Briefly, protein samples were prepared from neonatal rat cardiac cells and mice hearts, and were denatured and separated on 10% polyacrylamide gels by SDS-PAGE, and then transferred to polyvinylidene difluoride (PVDF) membranes. Target proteins were detected using standard procedures. Band intensities were normalized to housekeeping gene Histone H3 or β-actin. The following primary antibodies were used in this study: anti-Angpt4 (Invitrogen), anti-β-actin (Abclonal), anti-pERK (Cell Signaling Technology), and anti-Histone H3 (Beyotime).

### Adeno-associated virus *in vivo* delivery

Adeno-associated virus 9 (AAV9) vectors expressing *ANGPT4* and *GFP* under the control of CMV promoter were constructed by Vigene Biosciences. Mice in different groups were injected into tail vein with 10^12^ Vg of AAV9-*ANGPT4* or AAV9-*GFP*, respectively. Three weeks after AAV9 injection, we performed myocardial infarction as previously reported (Tarnavski et al., 2004).

### Echocardiography

Echocardiographic analysis was performed at 2 weeks and 6 weeks after MI with a Vevo2100 digital imaging system (Visual Sonics). Mice were anaesthetized under 1% isoflurane, with mid-ventricular M and B mode measurements acquired in the parasternal short-axis view at the level of the papillary muscles. Once the mice were accommodated to the procedures, images were stored for further analysis. LVIDd were recorded at the time of apparent maximal left ventricular diastolic dimension, while LVIDs were recorded at the time of the most anterior systolic excursion of the posterior wall. Left ventricular EF was calculated by LVEF (%) = ((LVIDd)^3^ − (LVIDs)^3^)/(LVIDd)^3^ × 100%, and left ventricular FS was calculated by LVFS (%)=(LVIDd – LVIDs)/LVIDd × 100%. The data were averaged from five cardiac cycles. We then calculated the difference between 6 weeks and 2 weeks of LVEF and LVFS to represent the recovery of heart function.

## Supplementary Material

pwac010_suppl_Supplementary_Figures

pwac010_suppl_Supplementary_Table_S1

pwac010_suppl_Supplementary_Table_S2

pwac010_suppl_Supplementary_Table_S3

## Abbreviations

AAV: adeno-associated virus
angpt4: angiopoietin 4
CM-A: atrial cardiomyocyte
CM-V: ventricular cardiomyocyte
CRISPR: clustered regularly interspaced short palindromic repeat
dpf: days post-fertilization
dpi: days post-injury
dpt: days post-treatment
EC: endocardial cell
EMT: epithelial-mesenchymal transition
EP: epicardial cell
EPDC: epicardium-derived cell
MI: myocardial infarction
MTZ: metronidazole
NRCM: neonatal rat cardiomyocyte
NTR: nitroreductase
RIC: regeneration-induced cell
t-SNE: t-distributed stochastic neighbor embedding

## Declarations

This work was partially supported by grants from the National Key Research and Development Program of China and the National Natural Science Foundation of China (NSFC) (Grant Nos. 2018YFA0801001, 32070824, 31871458, 2019YFA0802800, 2016YFA0100500, 31671500, 81371264, 31671177, and 2018YFA0800501).

A patent application has been filed relating to this work.

All animals are handled according to the regulation of Institutional Animal Care and Use Committee (IACUC) of Peking University. The Peking University IACUC reference number was LSC-ZhangB-2. All authors consent to participate in this study. All authors consent for publication of this study in Protein & Cell. All data and materials used in this study are available from the corresponding authors on reasonable request. The scRNA-seq data are available on SRA database: PRJNA629656, and R scripts used for graphic presentation are available from the corresponding authors on reasonable request.

Zekai Wu, Yuan Shi, Yueli Cui, Bo Zhang, Fuchou Tang, Shiqiang Wang and Yan Zhang conceived and designed the project. Zekai Wu, Yuan Shi and Yueli Cui performed the experiments including the collection of single cells, scRNA-seq library construction and functional experiments in zebrafish and mice with the assistance of Liya Zhang, Da Liu, Yutian Zhang and Li Jin. Yuan Shi performed the bioinformatics analyses with the help of Ji Dong. Xin Xing preformed the neonatal rat cardiac cell culture experiments. Meijun Pang generated the Tg (ubi:loxP-dsRed-STOP-loxP-EGFP) pku372 transgenic zebrafish. Rui-Ping Xiao, Jing-Wei Xiong, Xiangjun Tong and Zuoyan Zhu supervised the project. Bo Zhang, Fuchou Tang, Shiqiang Wang, Yan Zhang, Zekai Wu, Yuan Shi and Yueli Cui wrote the manuscript with feedback from all the other authors.

## References

[CIT0001] Bollini S , Vieira JM, Howard S et al. Re-activated adult epicardial progenitor cells are a heterogeneous population molecularly distinct from their embryonic counterparts. Stem Cells Dev 2014;23:1719–1730. 24702282 10.1089/scd.2014.0019

[CIT0002] Butler A , Hoffman P, Smibert P et al. Integrating single-cell transcriptomic data across different conditions, technologies, and species. Nat Biotechnol 2018;36:411–420. 29608179 10.1038/nbt.4096 PMC6700744

[CIT0003] Cahill TJ , Choudhury RP, Riley PR. Heart regeneration and repair after myocardial infarction: translational opportunities for novel therapeutics. Nat Rev Drug Discov 2017;16:699–717. 28729726 10.1038/nrd.2017.106

[CIT0004] Cai ZL , Liu C, Yao Q et al. The pro-migration and anti-apoptosis effects of HMGA2 in HUVECs stimulated by hypoxia. Cell Cycle 2020;19:3534–3545. 33315504 10.1080/15384101.2020.1850970 PMC7781619

[CIT0005] Cao J , Poss KD. The epicardium as a hub for heart regeneration. Nat Rev Cardiol 2018;15:631–647. 29950578 10.1038/s41569-018-0046-4 PMC6143401

[CIT0006] Chablais F , Veit J, Rainer G et al. The zebrafish heart regenerates after cryoinjury-induced myocardial infarction. BMC Dev Biol 2011;11:21. 21473762 10.1186/1471-213X-11-21 PMC3078894

[CIT0007] Chang N , Sun C, Gao L et al. Genome editing with RNA-guided Cas9 nuclease in zebrafish embryos. Cell Res 2013;23:465–472. 23528705 10.1038/cr.2013.45 PMC3616424

[CIT0008] Chi NC , Shaw RM, Jungblut B et al. Genetic and physiologic dissection of the vertebrate cardiac conduction system. PLoS Biol 2008;6:e109. 18479184 10.1371/journal.pbio.0060109 PMC2430899

[CIT0009] Cho CH , Sung HK, Kim KT et al. COMP-angiopoietin-1 promotes wound healing through enhanced angiogenesis, lymphangiogenesis, and blood flow in a diabetic mouse model. Proc Natl Acad Sci USA 2006;103:4946–4951. 16543381 10.1073/pnas.0506352103 PMC1458775

[CIT0010] Cui Y , Zheng Y, Liu X et al. Single-cell transcriptome analysis maps the developmental track of the human heart. Cell Rep 2019;26:1934–1950.e5. 30759401 10.1016/j.celrep.2019.01.079

[CIT0011] Davis S , Aldrich TH, Jones PF et al. Isolation of angiopoietin-1, a ligand for the TIE2 receptor, by secretion-trap expression cloning. Cell 1996;87:1161–1169. 8980223 10.1016/s0092-8674(00)81812-7

[CIT0012] de Bakker DEM , Bouwman M, Dronkers E et al. Prrx1b restricts fibrosis and promotes Nrg1-dependent cardiomyocyte proliferation during zebrafish heart regeneration. Development 2021;148:dev198937. 34486669 10.1242/dev.198937 PMC8513610

[CIT0013] Dobin A , Davis CA, Schlesinger F et al. STAR: ultrafast universal RNA-seq aligner. Bioinformatics 2013;29:15–21. 23104886 10.1093/bioinformatics/bts635 PMC3530905

[CIT0014] Duan J , Gherghe C, Liu D et al. Wnt1/betacatenin injury response activates the epicardium and cardiac fibroblasts to promote cardiac repair. EMBO J 2012;31:429–442. 22085926 10.1038/emboj.2011.418 PMC3261567

[CIT0015] Elamaa H , Kihlstrom M, Kapiainen E et al. Angiopoietin-4-dependent venous maturation and fluid drainage in the peripheral retina. Elife 2018;7:e37776. 30444491 10.7554/eLife.37776 PMC6239434

[CIT0016] Fang W , He A, Xiang MX et al. Cathepsin K-deficiency impairs mouse cardiac function after myocardial infarction. J Mol Cell Cardiol 2019;127:44–56. 30465799 10.1016/j.yjmcc.2018.11.010

[CIT0017] Feng L , Hernandez RE, Waxman JS et al. Dhrs3a regulates retinoic acid biosynthesis through a feedback inhibition mechanism. Dev Biol 2010;338:1–14. 19874812 10.1016/j.ydbio.2009.10.029 PMC2858591

[CIT0018] Fernandez CE , Bakovic M, Karra R. Endothelial contributions to zebrafish heart regeneration. J Cardiovasc Dev Dis 2018;5:56. 30544906 10.3390/jcdd5040056 PMC6306804

[CIT0019] Fink M , Callol-Massot C, Chu A et al. A new method for detection and quantification of heartbeat parameters in Drosophila, zebrafish, and embryonic mouse hearts. Biotechniques 2009;46:101–113. 19317655 10.2144/000113078 PMC2855226

[CIT0020] Gamba L , Amin-Javaheri A, Kim J et al. Collagenolytic activity is associated with scar resolution in zebrafish hearts after cryoinjury. J Cardiovasc Dev Dis 2017;4:2. 29367534 10.3390/jcdd4010002 PMC5715691

[CIT0021] Gemberling M , Karra R, Dickson AL et al. Nrg1 is an injury-induced cardiomyocyte mitogen for the endogenous heart regeneration program in zebrafish. Elife 2015;4:e05871. 25830562 10.7554/eLife.05871 PMC4379493

[CIT0022] Gene Ontology C. The Gene Ontology resource: enriching a GOld mine. Nucleic Acids Res 2021;49:D325–D334. 33290552 10.1093/nar/gkaa1113 PMC7779012

[CIT0023] Gonzalez-Rosa JM , Burns CE, Burnss CG. Zebrafish heart regeneration: 15 years of discoveries. Regeneration (Oxf) 2017;4:105–123. 28979788 10.1002/reg2.83 PMC5617908

[CIT0024] Gonzalez-Rosa JM , Martin V, Peralta M et al. Extensive scar formation and regression during heart regeneration after cryoinjury in zebrafish. Development 2011;138:1663–1674. 21429987 10.1242/dev.060897

[CIT0025] Grajevskaja V , Camerota D, Bellipanni G et al. Analysis of a conditional gene trap reveals that tbx5a is required for heart regeneration in zebrafish. PLoS One 2018;13:e0197293. 29933372 10.1371/journal.pone.0197293 PMC6014646

[CIT0026] Grun D , Muraro MJ, Boisset JC et al. De novo prediction of stem cell identity using single-cell transcriptome data. Cell Stem Cell 2016;19:266–277. 27345837 10.1016/j.stem.2016.05.010 PMC4985539

[CIT0027] Han P , Zhou XH, Chang N et al. Hydrogen peroxide primes heart regeneration with a derepression mechanism. Cell Res 2014;24:1091–1107. 25124925 10.1038/cr.2014.108 PMC4152734

[CIT0028] Honkoop H , de Bakker DE, Aharonov A et al. Single-cell analysis uncovers that metabolic reprogramming by ErbB2 signaling is essential for cardiomyocyte proliferation in the regenerating heart. Elife 2019;8:e50163. 31868166 10.7554/eLife.50163 PMC7000220

[CIT0029] Huang H , Bhat A, Woodnutt G et al. Targeting the ANGPT-TIE2 pathway in malignancy. Nat Rev Cancer 2010;10:575–585. 20651738 10.1038/nrc2894

[CIT0030] Huang S , Li X, Zheng H et al. Loss of super-enhancer-regulated circRNA Nfix induces cardiac regeneration after myocardial infarction in adult mice. Circulation 2019;139:2857–2876. 30947518 10.1161/CIRCULATIONAHA.118.038361 PMC6629176

[CIT0031] Ieda M , Tsuchihashi T, Ivey KN et al. Cardiac fibroblasts regulate myocardial proliferation through beta1 integrin signaling. Dev Cell 2009;16:233–244. 19217425 10.1016/j.devcel.2008.12.007 PMC2664087

[CIT0032] Itou J , Oishi I, Kawakami H et al. Migration of cardiomyocytes is essential for heart regeneration in zebrafish. Development 2012;139:4133–4142. 23034636 10.1242/dev.079756

[CIT0033] Jessup M , Brozena S. Heart failure. N Engl J Med 2003;348:2007–2018. 12748317 10.1056/NEJMra021498

[CIT0034] Jopling C , Sleep E, Raya M et al. Zebrafish heart regeneration occurs by cardiomyocyte dedifferentiation and proliferation. Nature 2010;464:606–609. 20336145 10.1038/nature08899 PMC2846535

[CIT0035] Ju BG , Kim WS. Upregulation of cathepsin D expression in the dedifferentiating salamander limb regenerates and enhancement of its expression by retinoic acid. Wound Repair Regen 1998;6:349–357. 9824553 10.1046/j.1524-475x.1998.60410.x

[CIT0036] Kalfon R , Friedman T, Eliachar S et al. JDP2 and ATF3 deficiencies dampen maladaptive cardiac remodeling and preserve cardiac function. PLoS One 2019;14:e0213081. 30818334 10.1371/journal.pone.0213081 PMC6394944

[CIT0037] Kawakami K , Takeda H, Kawakami N et al. A transposon-mediated gene trap approach identifies developmentally regulated genes in zebrafish. Dev Cell 2004;7:133–144. 15239961 10.1016/j.devcel.2004.06.005

[CIT0038] Kesler CT , Pereira ER, Cui CH et al. Angiopoietin-4 increases permeability of blood vessels and promotes lymphatic dilation. FASEB J 2015;29:3668–3677. 25977256 10.1096/fj.14-268920 PMC4550378

[CIT0039] Kikuchi K , Gupta V, Wang J et al. *tcf21*^+^ epicardial cells adopt non-myocardial fates during zebrafish heart development and regeneration. Development2011a;138:2895–2902. 21653610 10.1242/dev.067041 PMC3119303

[CIT0040] Kikuchi K , Holdway JE, Major RJ et al. Retinoic acid production by endocardium and epicardium is an injury response essential for zebrafish heart regeneration. Dev Cell2011b;20:397–404. 21397850 10.1016/j.devcel.2011.01.010 PMC3071981

[CIT0041] Lee HJ , Bae SW, Koh GY et al. COMP-Ang1, angiopoietin-1 variant protects radiation-induced bone marrow damage in C57BL/6 mice. J Radiat Res 2008;49:313–320. 18413981 10.1269/jrr.07064

[CIT0042] Lepilina A , Coon AN, Kikuchi K et al. A dynamic epicardial injury response supports progenitor cell activity during zebrafish heart regeneration. . Cell 2006;127:607–619. 17081981 10.1016/j.cell.2006.08.052

[CIT0043] Li L , Dong J, Yan L et al. Single-Cell RNA-Seq Analysis Maps Development of Human Germline Cells and Gonadal Niche Interactions. Cell Stem Cell 2017;20:891–892. 28575695 10.1016/j.stem.2017.05.009

[CIT0044] Li W , Zhang Y, Han B et al. One-step efficient generation of dual-function conditional knockout and geno-tagging alleles in zebrafish. Elife 2019;8:e48081. 31663848 10.7554/eLife.48081 PMC6845224

[CIT0045] Lie-Venema H , van den Akker NM, Bax NA et al. Origin, fate, and function of epicardium-derived cells (EPDCs) in normal and abnormal cardiac development. ScientificWorldJournal 2007;7:1777–1798. 18040540 10.1100/tsw.2007.294 PMC5901302

[CIT0046] Liu P , Zhong TP. MAPK/ERK signalling is required for zebrafish cardiac regeneration. Biotechnol Lett 2017;39:1069–1077. 28353145 10.1007/s10529-017-2327-0

[CIT0047] Lourenco AB , Artal-Sanz M. The mitochondrial prohibitin (PHB) complex in *C. elegans* metabolism and ageing regulation. Metabolites 2021;11:636. 34564452 10.3390/metabo11090636 PMC8472356

[CIT0048] Lu CJ , Fan XY, Guo YF et al. Single-cell analyses identify distinct and intermediate states of zebrafish pancreatic islet development. J Mol Cell Biol 2019;11:435–447. 30407522 10.1093/jmcb/mjy064 PMC6604604

[CIT0049] Missinato MA , Saydmohammed M, Zuppo DA et al. Dusp6 attenuates Ras/MAPK signaling to limit zebrafish heart regeneration. Development 2018;145:dev157206. 29444893 10.1242/dev.157206 PMC5868992

[CIT0050] Mosimann C , Kaufman CK, Li P et al. Ubiquitous transgene expression and Cre-based recombination driven by the ubiquitin promoter in zebrafish. Development 2011;138:169–177. 21138979 10.1242/dev.059345 PMC2998170

[CIT0051] Paffett-Lugassy N , Novikov N, Jeffrey S et al. Unique developmental trajectories and genetic regulation of ventricular and outflow tract progenitors in the zebrafish second heart field. Development 2017;144:4616–4624. 29061637 10.1242/dev.153411 PMC5769620

[CIT0052] Parmar D , Apte M. Angiopoietin inhibitors: a review on targeting tumor angiogenesis. Eur J Pharmacol 2021;899:174021. 33741382 10.1016/j.ejphar.2021.174021

[CIT0053] Picelli S , Faridani OR, Bjorklund AK et al. Full-length RNA-seq from single cells using Smart-seq2. Nat Protoc 2014;9:171–181. 24385147 10.1038/nprot.2014.006

[CIT0054] Porrello ER , Mahmoud AI, Simpson E et al. Transient regenerative potential of the neonatal mouse heart. Science 2011;331:1078–1080. 21350179 10.1126/science.1200708 PMC3099478

[CIT0055] Poss KD , Wilson LG, Keating MT. Heart regeneration in zebrafish. Science 2002;298:2188–2190. 12481136 10.1126/science.1077857

[CIT0056] Ren P , Xing L, Hong X et al. LncRNA PITPNA-AS1 boosts the proliferation and migration of lung squamous cell carcinoma cells by recruiting TAF15 to stabilize HMGB3 mRNA. Cancer Med 2020;9:7706–7716. 32871048 10.1002/cam4.3268 PMC7571819

[CIT0057] Sallin P , de Preux Charles AS, Duruz V et al. A dual epimorphic and compensatory mode of heart regeneration in zebrafish. Dev Biol 2015;399:27–40. 25557620 10.1016/j.ydbio.2014.12.002

[CIT0058] Saneshige S , Mano H, Tezuka K et al. Retinoic acid directly stimulates osteoclastic bone resorption and gene expression of cathepsin K/OC-2. Biochem J 1995;309:721–724. 7639684 10.1042/bj3090721 PMC1135691

[CIT0059] Schindler YL , Garske KM, Wang J et al. Hand2 elevates cardiomyocyte production during zebrafish heart development and regeneration. Development 2014;141:3112–3122. 25038045 10.1242/dev.106336 PMC4197543

[CIT0060] Schnabel K , Wu CC, Kurth T et al. Regeneration of cryoinjury induced necrotic heart lesions in zebrafish is associated with epicardial activation and cardiomyocyte proliferation. PLoS One 2011;6:e18503. 21533269 10.1371/journal.pone.0018503 PMC3075262

[CIT0061] Sierpinski R , Josiak K, Suchocki T et al. High soluble transferrin receptor in patients with heart failure: a measure of iron deficiency and a strong predictor of mortality. Eur J Heart Fail 2021;23:919–932. 33111457 10.1002/ejhf.2036

[CIT0062] Smith T , Heger A, Sudbery I. UMI-tools: modeling sequencing errors in Unique Molecular Identifiers to improve quantification accuracy. Genome Res 2017;27:491–499. 28100584 10.1101/gr.209601.116 PMC5340976

[CIT0063] Song Y , Xu C, Liu J et al. Heterodimerization With 5-HT2BR Is Indispensable for beta2AR-Mediated Cardioprotection. Circ Res 2021;128:262–277. 33208036 10.1161/CIRCRESAHA.120.317011

[CIT0064] Tahara N , Brush M, Kawakami Y. Cell migration during heart regeneration in zebrafish. Dev Dyn 2016;245:774–787. 27085002 10.1002/dvdy.24411 PMC5839122

[CIT0065] Tao G , Kahr PC, Morikawa Y et al. Pitx2 promotes heart repair by activating the antioxidant response after cardiac injury. Nature 2016;534:119–123. 27251288 10.1038/nature17959 PMC4999251

[CIT0066] Tarnavski O , McMullen JR, Schinke M et al. Mouse cardiac surgery: comprehensive techniques for the generation of mouse models of human diseases and their application for genomic studies. Physiol Genomics 2004;16:349–360. 14679301 10.1152/physiolgenomics.00041.2003

[CIT0067] Thiery JP , Acloque H, Huang RY et al. Epithelial-mesenchymal transitions in development and disease. Cell 2009;139:871–890. 19945376 10.1016/j.cell.2009.11.007

[CIT0068] Tirosh I , Izar B, Prakadan SM et al. Dissecting the multicellular ecosystem of metastatic melanoma by single-cell RNA-seq. Science 2016;352:189–196. 27124452 10.1126/science.aad0501 PMC4944528

[CIT0069] Tong X , Zu Y, Li Z et al. Kctd10 regulates heart morphogenesis by repressing the transcriptional activity of Tbx5a in zebrafish. Nat Commun 2014;5:3153. 24430697 10.1038/ncomms4153

[CIT0070] Trapnell C , Cacchiarelli D, Grimsby J et al. The dynamics and regulators of cell fate decisions are revealed by pseudotemporal ordering of single cells. Nat Biotechnol 2014;32:381–386. 24658644 10.1038/nbt.2859 PMC4122333

[CIT0071] Uygur A , Lee RT. Mechanisms of Cardiac Regeneration. Dev Cell 2016;36:362–374. 26906733 10.1016/j.devcel.2016.01.018 PMC4768311

[CIT0072] Vieira JM , Howard S, Villa Del Campo C et al. BRG1-SWI/SNF-dependent regulation of the Wt1 transcriptional landscape mediates epicardial activity during heart development and disease. Nat Commun 2017;8:16034. 28737171 10.1038/ncomms16034 PMC5527284

[CIT0073] Wang J , Cao J, Dickson AL et al. Epicardial regeneration is guided by cardiac outflow tract and Hedgehog signalling. Nature 2015;522:226–230. 25938716 10.1038/nature14325 PMC4494087

[CIT0074] Wang J , Karra R, Dickson AL et al. Fibronectin is deposited by injury-activated epicardial cells and is necessary for zebrafish heart regeneration. Dev Biol2013a;382:427–435. 23988577 10.1016/j.ydbio.2013.08.012 PMC3852765

[CIT0075] Wang J , Panakova D, Kikuchi K et al. The regenerative capacity of zebrafish reverses cardiac failure caused by genetic cardiomyocyte depletion. Development 2011;138:3421–3430. 21752928 10.1242/dev.068601 PMC3143562

[CIT0076] Wang L , Liu T, Xu L et al. Fev regulates hematopoietic stem cell development via ERK signaling. Blood2013b;122:367–375. 23591790 10.1182/blood-2012-10-462655

[CIT0077] Wu RS , Lam II, Clay H et al. A Rapid Method for Directed Gene Knockout for Screening in G0 Zebrafish. Dev Cell 2018;46:112–125. 29974860 10.1016/j.devcel.2018.06.003

[CIT0078] Xu W , Barrientos T, Mao L et al. Lethal Cardiomyopathy in Mice Lacking Transferrin Receptor in the Heart. Cell Rep 2015;13:533–545. 26456827 10.1016/j.celrep.2015.09.023 PMC4618069

[CIT0079] Youn SW , Lee HC, Lee SW et al. COMP-Angiopoietin-1 accelerates muscle regeneration through N-cadherin activation. Sci Rep 2018;8:12323. 30120297 10.1038/s41598-018-30513-7 PMC6098079

[CIT0080] Zhang C , Chen Y, Sun B et al. m(6)A modulates haematopoietic stem and progenitor cell specification. Nature 2017;549:273–276. 28869969 10.1038/nature23883

[CIT0081] Zhang R , Han P, Yang H et al. In vivo cardiac reprogramming contributes to zebrafish heart regeneration. Nature 2013;498:497–501. 23783515 10.1038/nature12322 PMC4090927

[CIT0082] Zhao L , Borikova AL, Ben-Yair R et al. Notch signaling regulates cardiomyocyte proliferation during zebrafish heart regeneration. Proc Natl Acad Sci USA 2014;111:1403–1408. 24474765 10.1073/pnas.1311705111 PMC3910613

[CIT0083] Zhong S , Zhang S, Fan X et al. A single-cell RNA-seq survey of the developmental landscape of the human prefrontal cortex. Nature 2018;555:524–528. 29539641 10.1038/nature25980

[CIT0084] Zhou Y , Zhou B, Pache L et al. Metascape provides a biologist-oriented resource for the analysis of systems-level datasets. Nat Commun 2019;10:1523. 30944313 10.1038/s41467-019-09234-6 PMC6447622

