# Supplementary material for: Single-cell analysis reveals an Angpt4-initiated EPDC-EC-CM cellular coordination cascade during heart regeneration
Source: Protein Cell. 2022 May 18;14(5):350–68. doi: 10.1093/procel/pwac010 (PMC10166170; doi:10.1093/procel/pwac010)
Supplement: pwac010_suppl_Supplementary_Figures [file pwac010_suppl_supplementary_figures.docx]

**
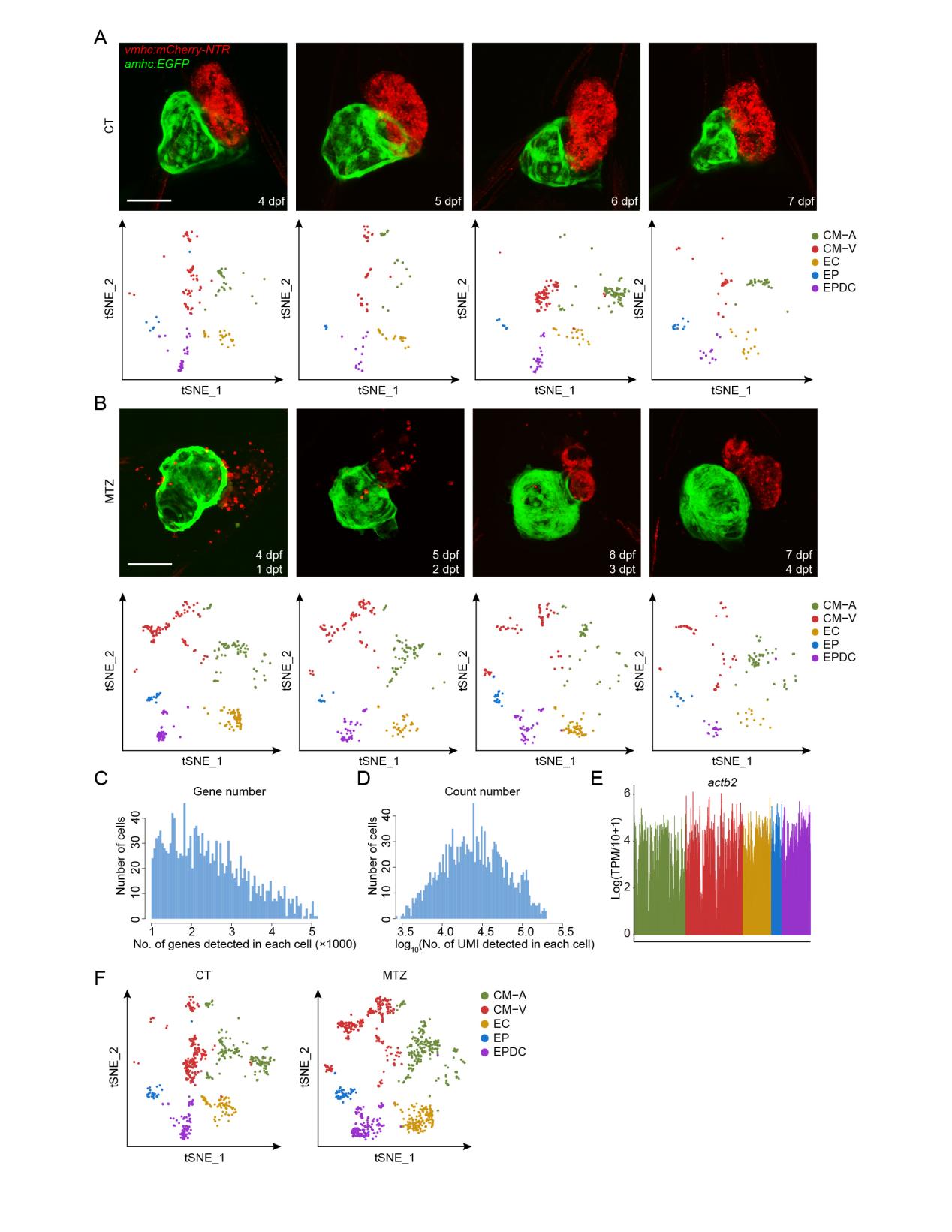
Figure S1. Genetic ablation of ventricular CM causes specific ventricular CM death and subsequent regeneration in the zebrafish embryo.**

**(A and B)** Maximum intensity projections of *Tg(vmhc:mCherry-NTR; amhc:EGFP)* zebrafish embryos, showing the process of normal heart development (**A**) and cardiac regeneration after MTZ treatment (**B**). Alongside, *t-SNE* analysis showed the distribution of cells at the corresponding developmental or regeneration stage. Cluster identification and *t-SNE* coordinates are the same as in **Figure 1B**. dpt, days post-treatment. Red, ventricular cardiomyocytes; Green, atrial cardiomyocytes; Scale bar, 50 μm.

**(C and D)** Histograms showing the distribution of numbers of genes (**C**) and unique molecular identifiers (UMI) (**D**) detected in each cell.

**(E)** Line plot showing the uniform expression pattern of housekeeping gene *actb2* in all sequenced cells.

**(F)** Separate *t-SNE* maps of cells from Fig. 1B, showing the distribution of untreated (CT) and MTZ-treated cells in zebrafish embryos. Each dot represents one cell and colored by clusters.

**
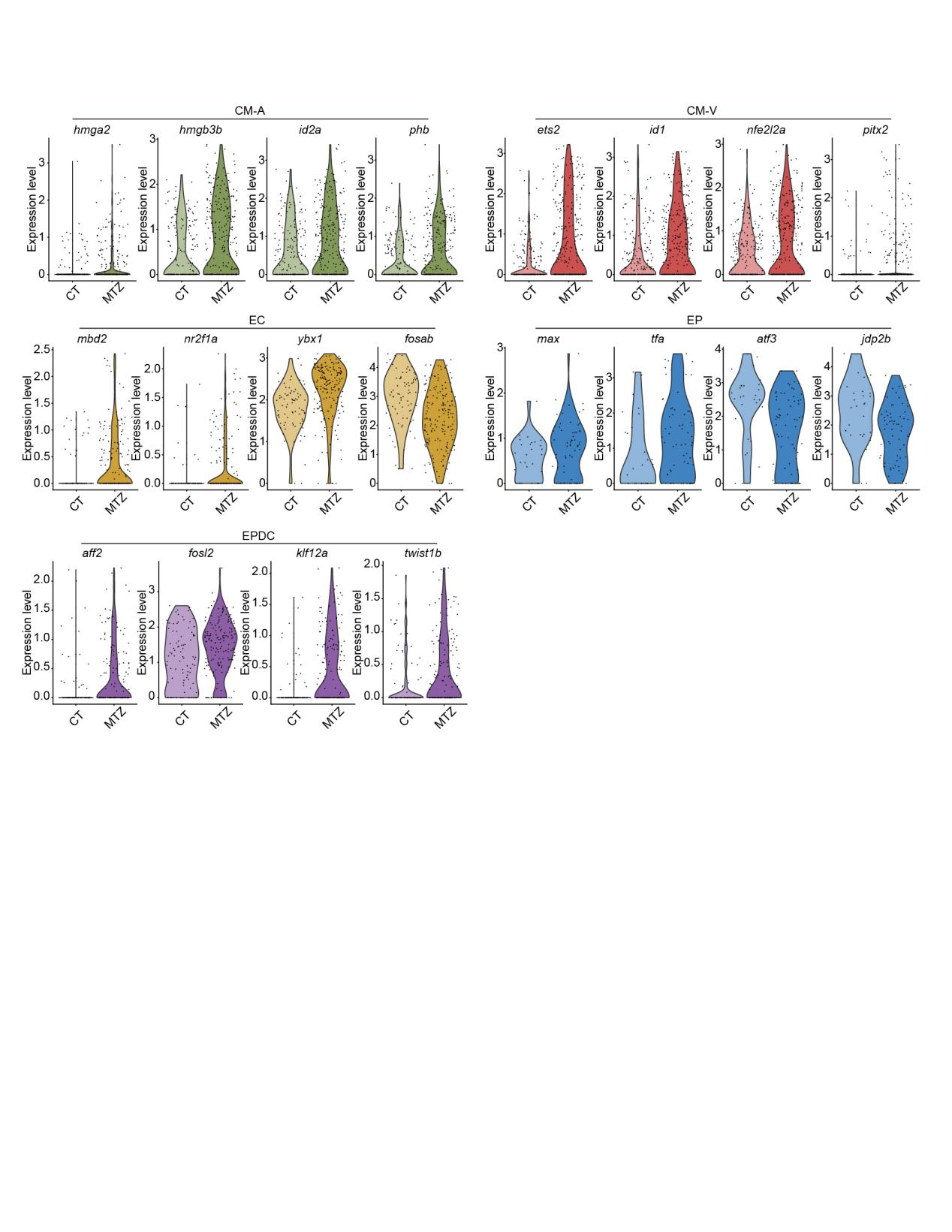
Figure S2. Representative differentially expressed transcription factor genes during zebrafish heart development and regeneration derived from scRNA-seq data.**

Violin plots showing representative transcription factor genes differentially expressed in CT and MTZ cells from CM-A, CM-V, EC, EP or EPDC clusters.

**
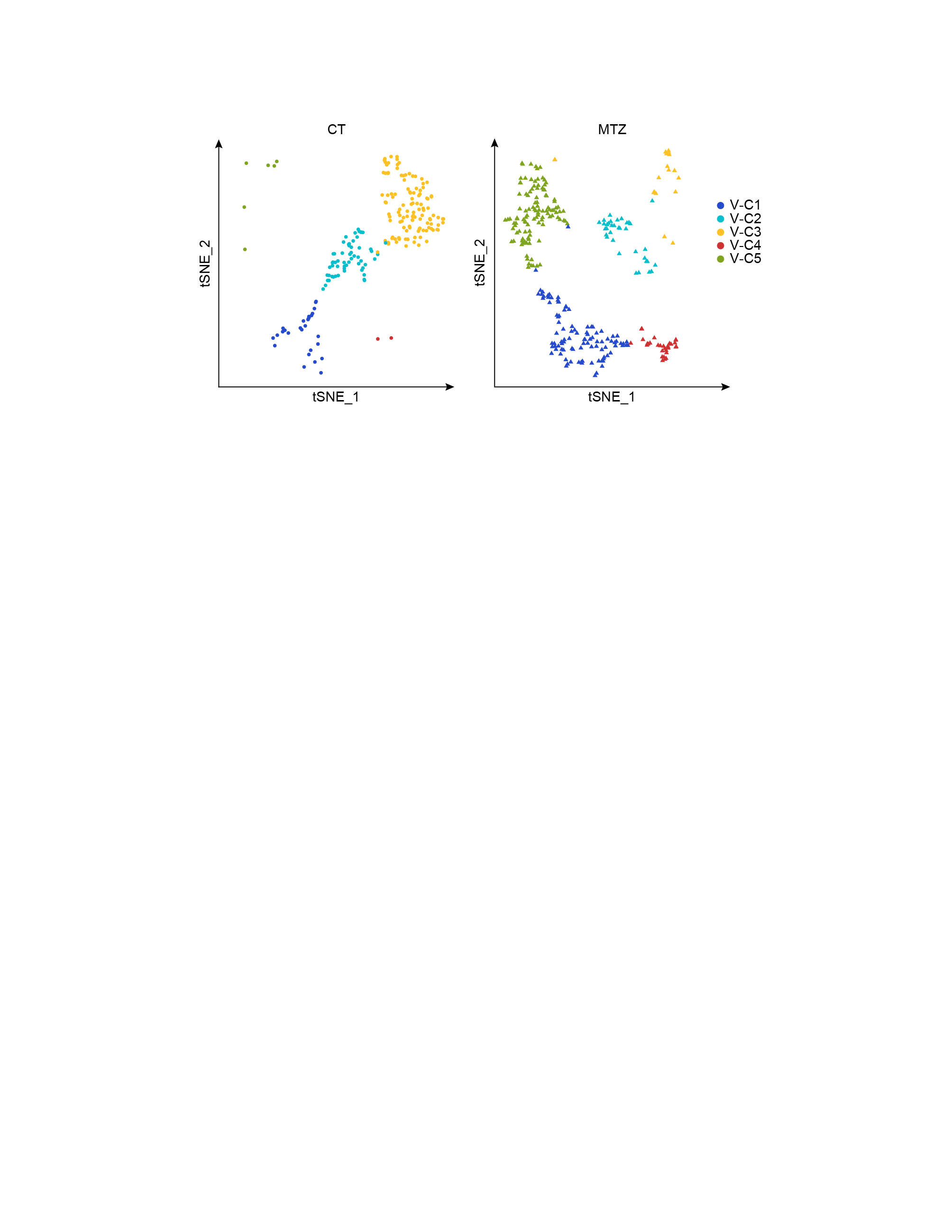
Figure S3. *t-SNE* analysis of CM-V during zebrafish heart development and regeneration.**

Separate *t-SNE* maps of CM-V populations from Fig. 2A, showing the distribution of untreated (CT) and MTZ-treated CM-V cells in zebrafish embryos. Each dot represents one cell, and colored by clusters and shaped by sample conditions. *n* = 510 cells.

**
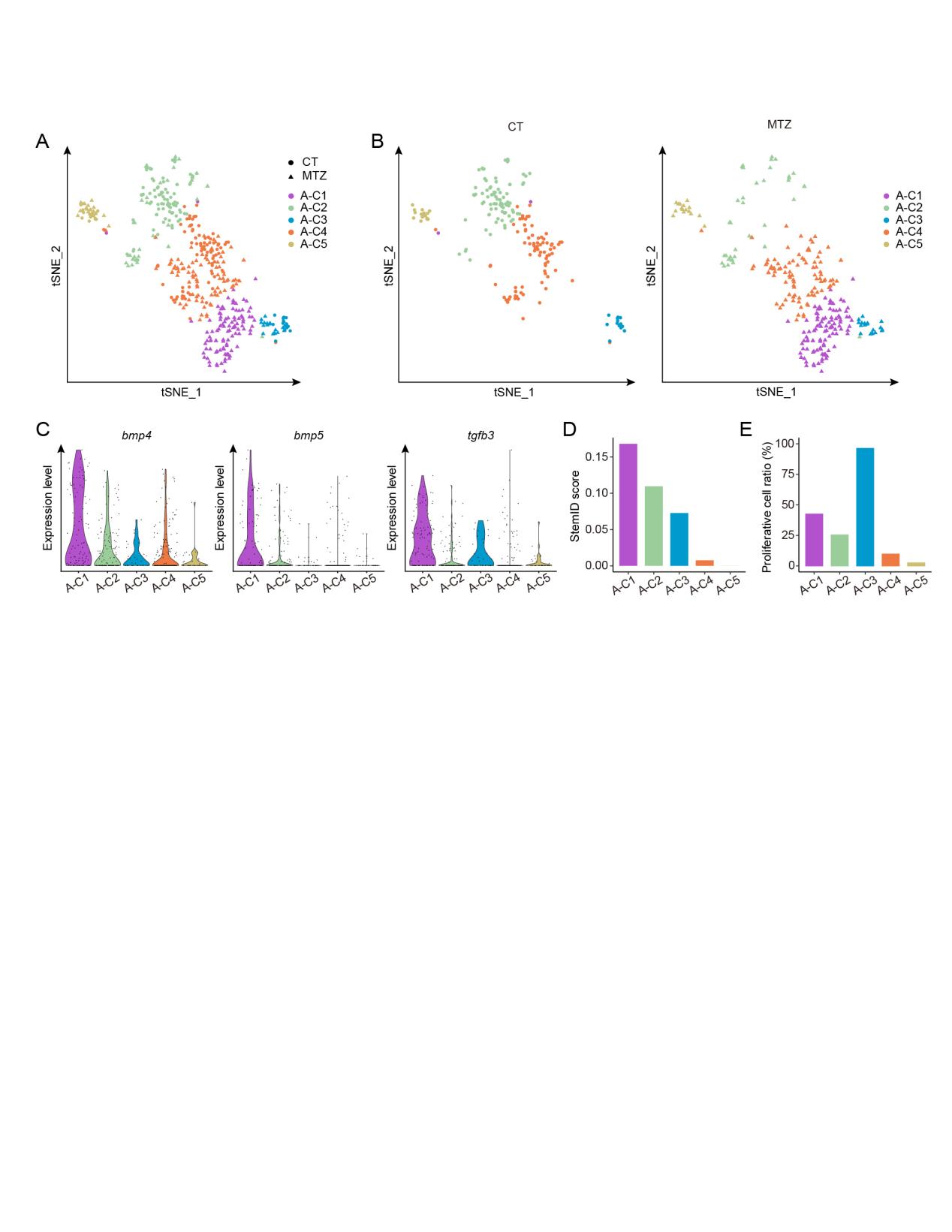
Figure S4. Molecular characteristics of atrial cardiomyocytes during larval zebrafish heart regeneration.**

**(A)** *t-SNE* analysis results of CM-A from both untreated (CT) and MTZ-treated zebrafish embryos, colored by clusters and shaped by sample conditions.

**(B)** Separate *t-SNE* maps of CM-A from **(A)**, showing the distribution of cells from untreated (CT) and MTZ-treated zebrafish embryos, colored by clusters and shaped by sample conditions.

**(C)** Violin plots showing the expression patterns of *bmp4*, *bmp5*, and *tgfb3* in different subpopulations of CM-A.

**(D and E)** StemID analysis **(D)** and cell cycle analysis **(E)** of CM-A subpopulations.

**
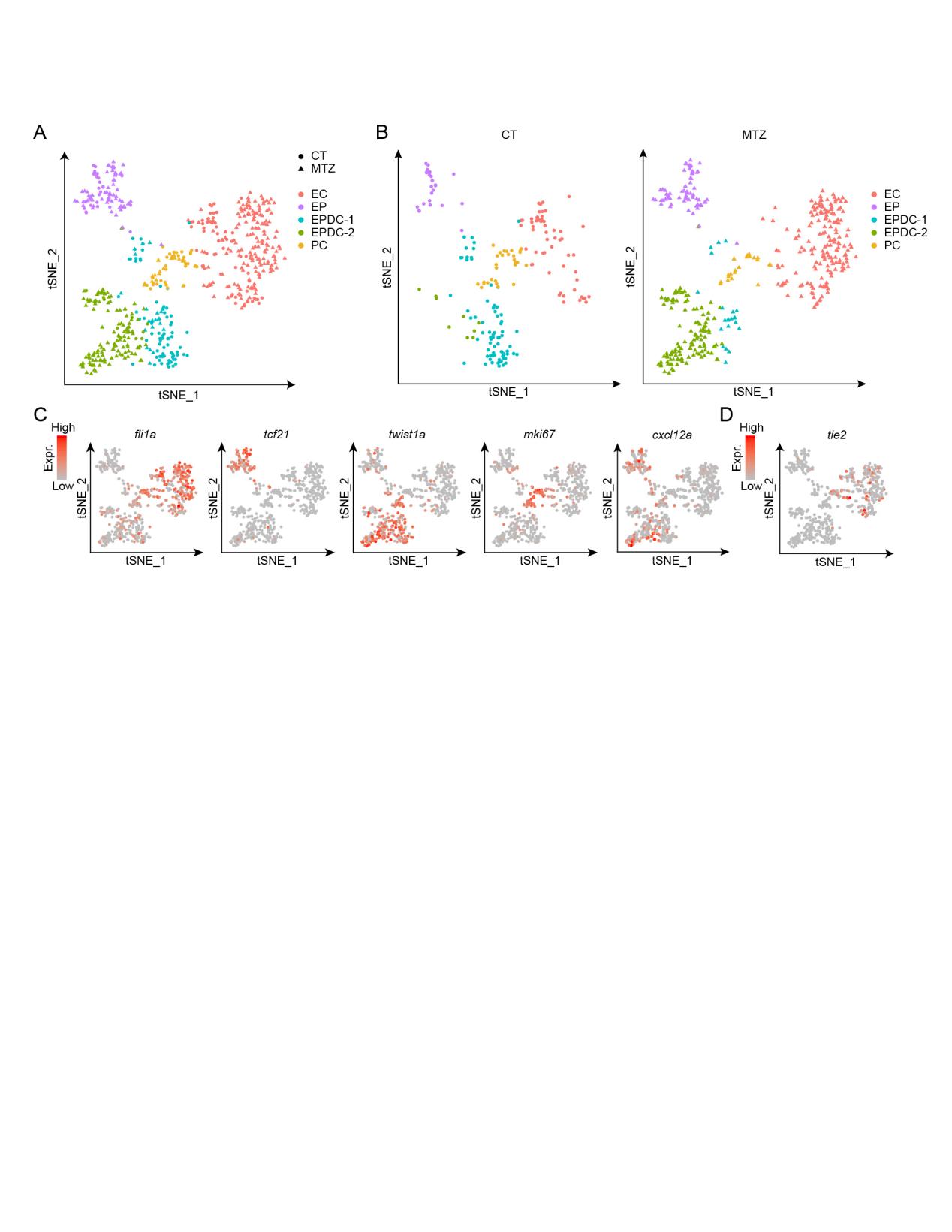
Figure S5. Molecular features of the non-muscle cardiac cells** **during zebrafish heart development and regeneration.**

**(A and B)** *t-SNE* analysis showing heterogeneity of the non-muscle cardiac cells, shaped by experimental conditions (CT or MTZ). The EPDC could be subdivided into two clusters, EPDC-C1 and EPDC-C2. CT, untreated control embryos; MTZ, MTZ-treated embryos.

**(C)** The expression pattern of some non-muscle cardiac cell marker genes displayed in the *t-SNE* map.

**(D)** *t-SNE* map showing the expression pattern of *tie2.*

**
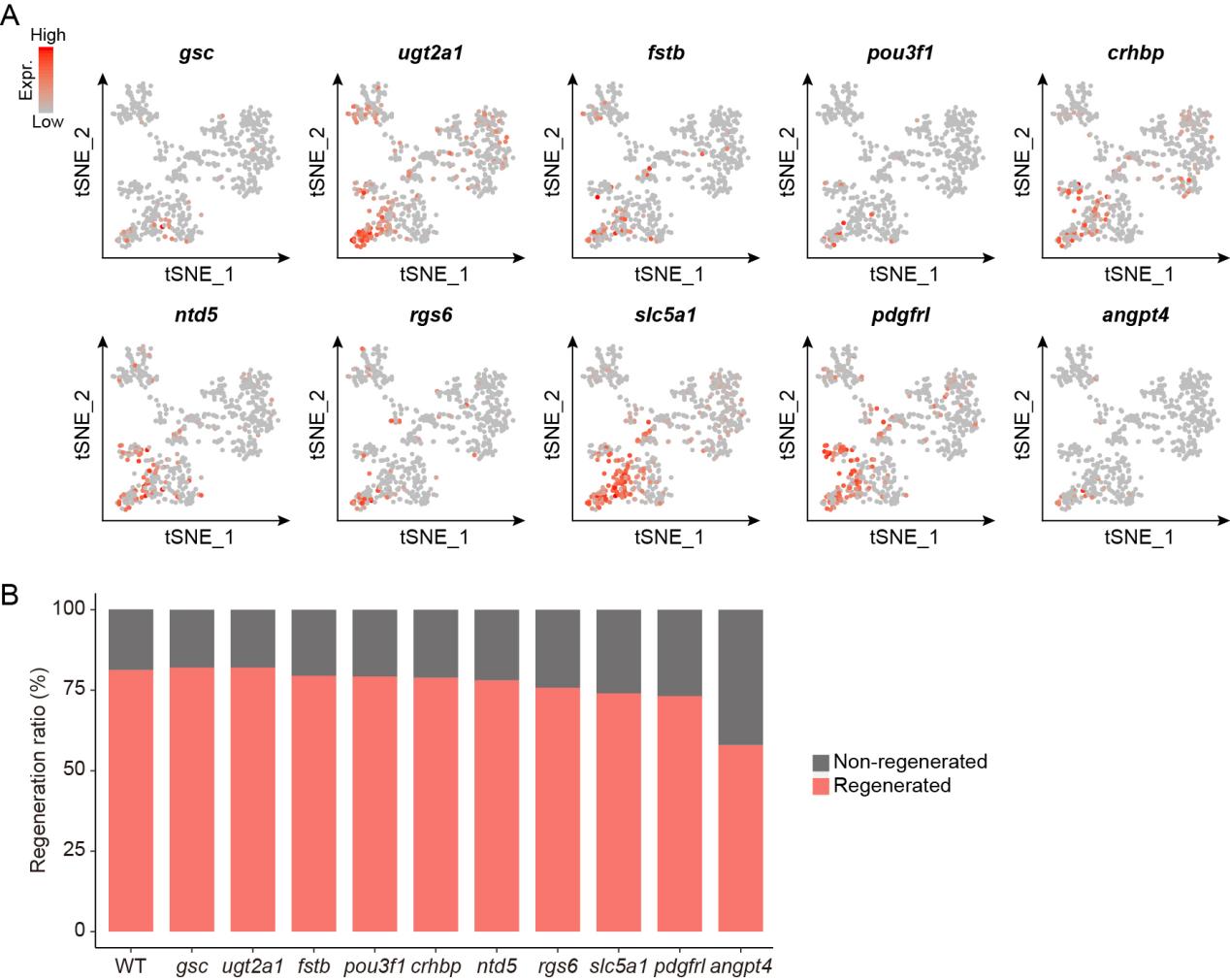
Figure S6. The expression pattern and functional screen of genes specifically up-regulated in the RIC population.**

**(A)** The expression pattern shown in the *t-SNE* map of the ten genes selected for functional screening by the CRISPR/Cas9 knockout strategy.

**(B)** Successful rate of heart regeneration in WT and mosaic zebrafish embryos after injection of Cas9 protein and 4x gRNAs targeting each corresponding gene in **(A)**.

**
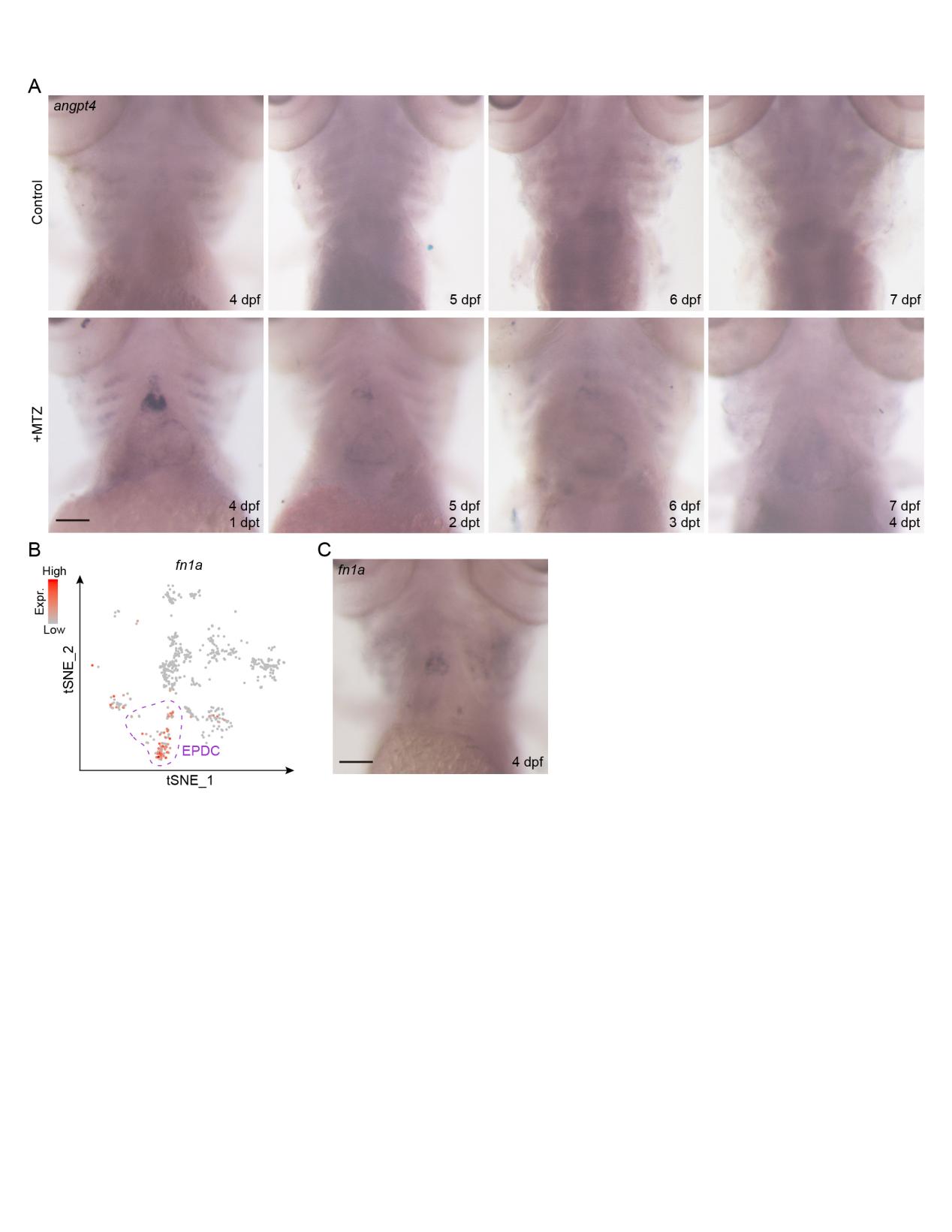
Figure S7. *angpt4* was up-regulated during larval zebrafish heart regeneration.**

**(A)** Whole-mount *in situ* hybridization results showed expression pattern of *angpt4* in *Tg(vmhc:mCherry-NTR; amhc:EGFP)* zebrafish heart during normal development and after MTZ treatment. The embryos are shown as ventral view with anterior on top. Scale bar, 100 μm.

**(B)** *t-SNE* map of control cells from Fig. 1B showed *fn1a* was highly expressed in the EPDC population during zebrafish heart development.

**(C)** Whole-mount *in situ* hybridization result showed *fn1a* was mainly expressed in the OFT region in 4 dpf zebrafish heart during normal development. Scale bar, 100 μm.


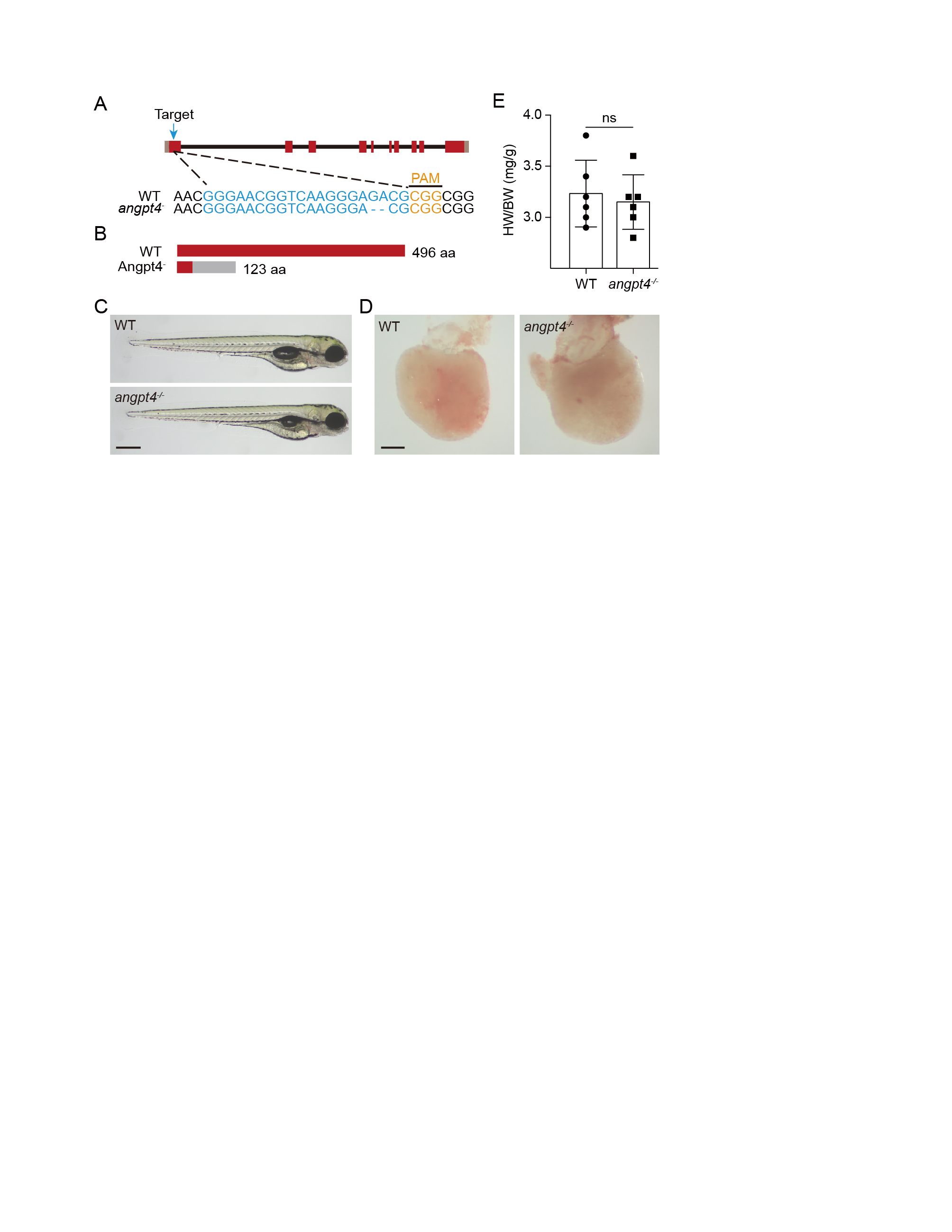


**Figure S8. *angpt4* is dispensable for zebrafish normal development.**

**(A)** Schematic diagram of zebrafish *angpt4* gene structure and the CRISPR/Cas9 target site as well as the mutant allele sequence. A 2-bp deletion in exon 1 was identified in the stable *angpt4* mutant. Blue letters represent the CRISPR/Cas9 target site. Red, coding sequence. Brown, untranslated region.

**(B)** Predicted truncated protein product of the 2-bp deletion allele of *angpt4*. Red, in-frame peptide sequence. Grey, the peptide encoded by the frame-shifted coding sequence.

**(C)** Embryo morphology of wildtype and *angpt4^-/-^* zebrafish embryos at 4 dpf. Scale bar, 400 μm.

**(D)** Heart morphology of wildtype and *angpt4^-/-^* adult zebrafish. Scale bar, 200 μm.

**(E)** Quantification results of heart weight (HW) to body weight (BW) ratio of wildtype and *angpt4^-/-^* zebrafish embryos. Two-tailed Student’s *t*-test, ns, not significant. *n* = 6 for each group. Error bar represents standard deviation.


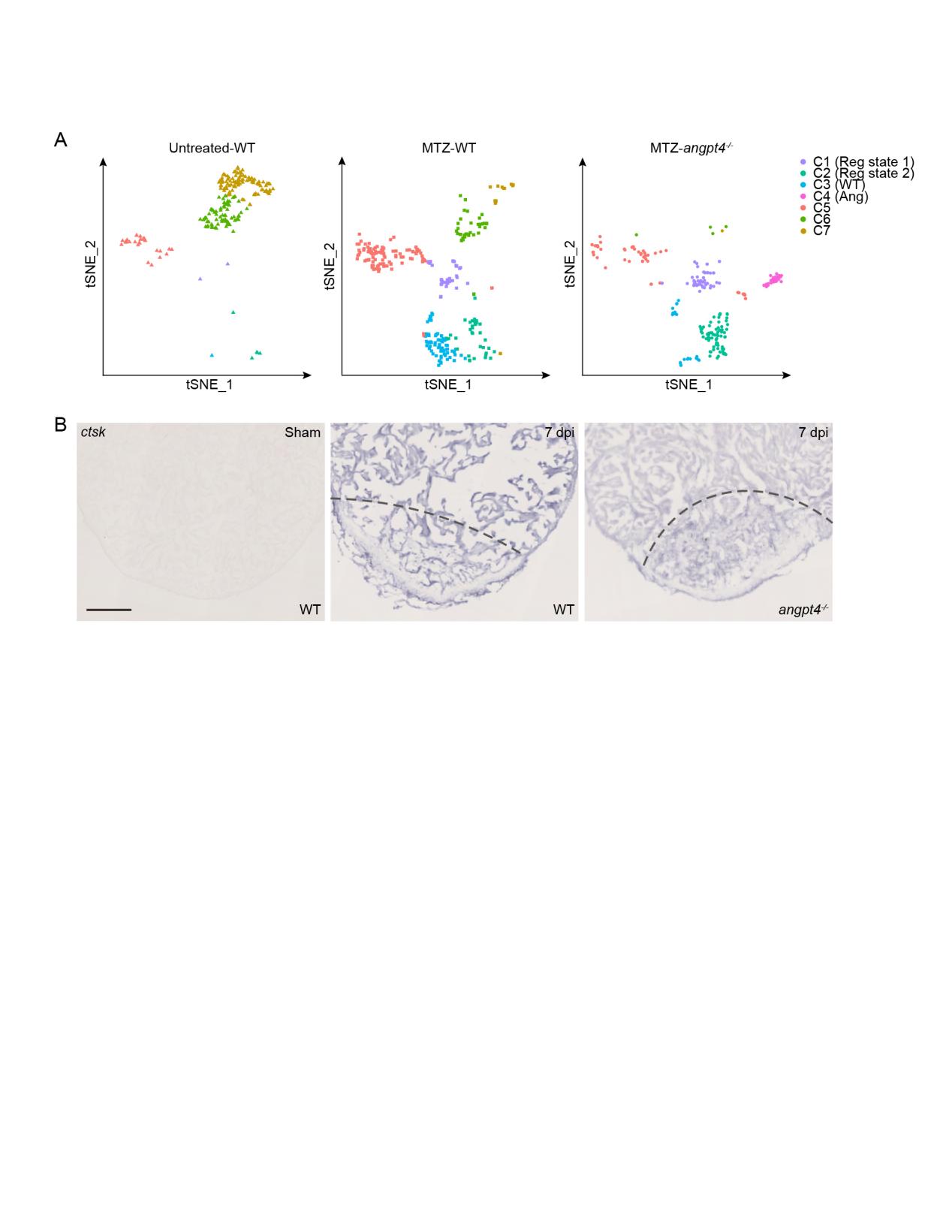


**Figure S9. *t-SNE* analysis of CM-V from WT zebrafish and *angpt4* mutants.**

**(A)** Separate *t-SNE* maps of CM-V populations from Fig. 5A, showing the distribution of CM-V cells in control (untreated) and MTZ-treated WT embryos, and MTZ-treated *angpt4* mutants. Cells are colored by clusters and shaped by sample conditions.

**(B)** *In situ* hybridization results showing expression pattern of *ctsk* in sham-operated WT, WT at 7 dpi, and *angpt4* mutants at 7 dpi. Representative images of three replicates are shown. Scale bar, 100 μm.

**
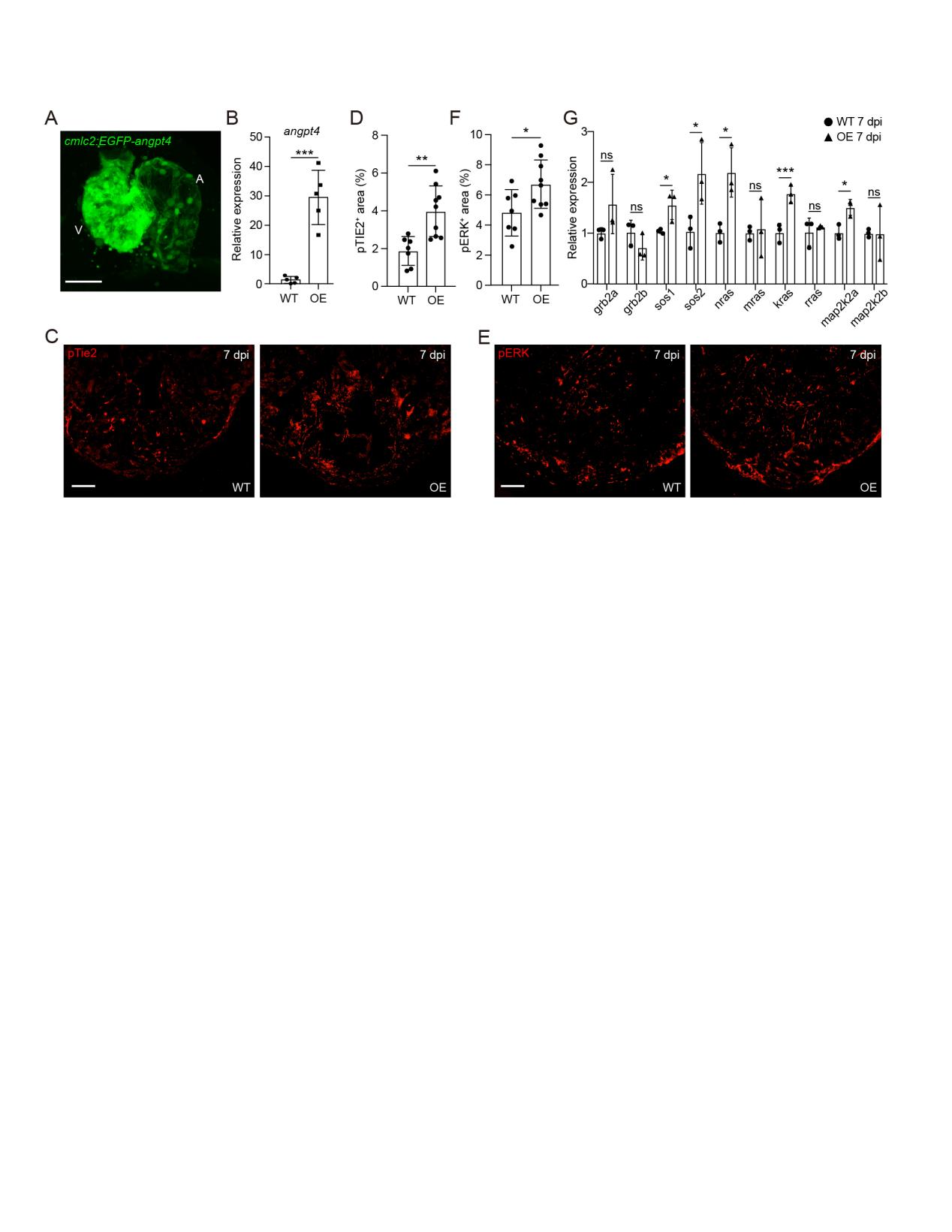
**

**Figure S10. *angpt4* overexpression activates pTie2 and pERK signaling during zebrafish heart regeneration.**

**(A)** Maximum intensity projection of the heart from *Tg(cmlc2:EGFP-angpt4)* fish at 3 dpf. Scale bar, 50 μm.

**(B)** qRT-PCR results of *angpt4* expression level in WT and *Tg(cmlc2:EGFP-angpt4)* embryos at 3 dpf. OE, *Tg(cmlc2:EGFP-angpt4)* fish. n = 5 for each group. Two-tailed Student’s *t*-test, *** *p* < 0.001.

**(C)** Immunofluorescence staining showing pTie2 signals in WT and *angpt4* OE hearts at 7 dpi. Scale bar, 100 μm.

**(D)** Statistical analysis results of relative pTie2 signals in WT and *angpt4* OE hearts at 7 dpi from **(C)**. Two-tailed Student’s *t*-test, ** *p* < 0.01. n = 7-9 for each group. Error bar represents standard deviation.

**(E)** Immunofluorescence staining showing pERK signals in WT and *angpt4* OE hearts at 7 dpi. Scale bar, 100 μm.

**(F)** Statistical analysis results of relative pERK signals in WT and *angpt4* OE hearts at 7 dpi from **(E)**. Two-tailed Student’s t-test, * *p* < 0.05. n = 7-9 for each group. Error bar represents standard deviation.

**(G)** qRT-PCR results showing the expression level of several genes in the MAPK signaling pathway in WT and *angpt4* OE hearts at 7 dpi. Two-tailed Student’s t-test, ns, not significant, * *p* < 0.05, *** *p* < 0.001. n = 3 for each group. Error bar represents standard deviation.


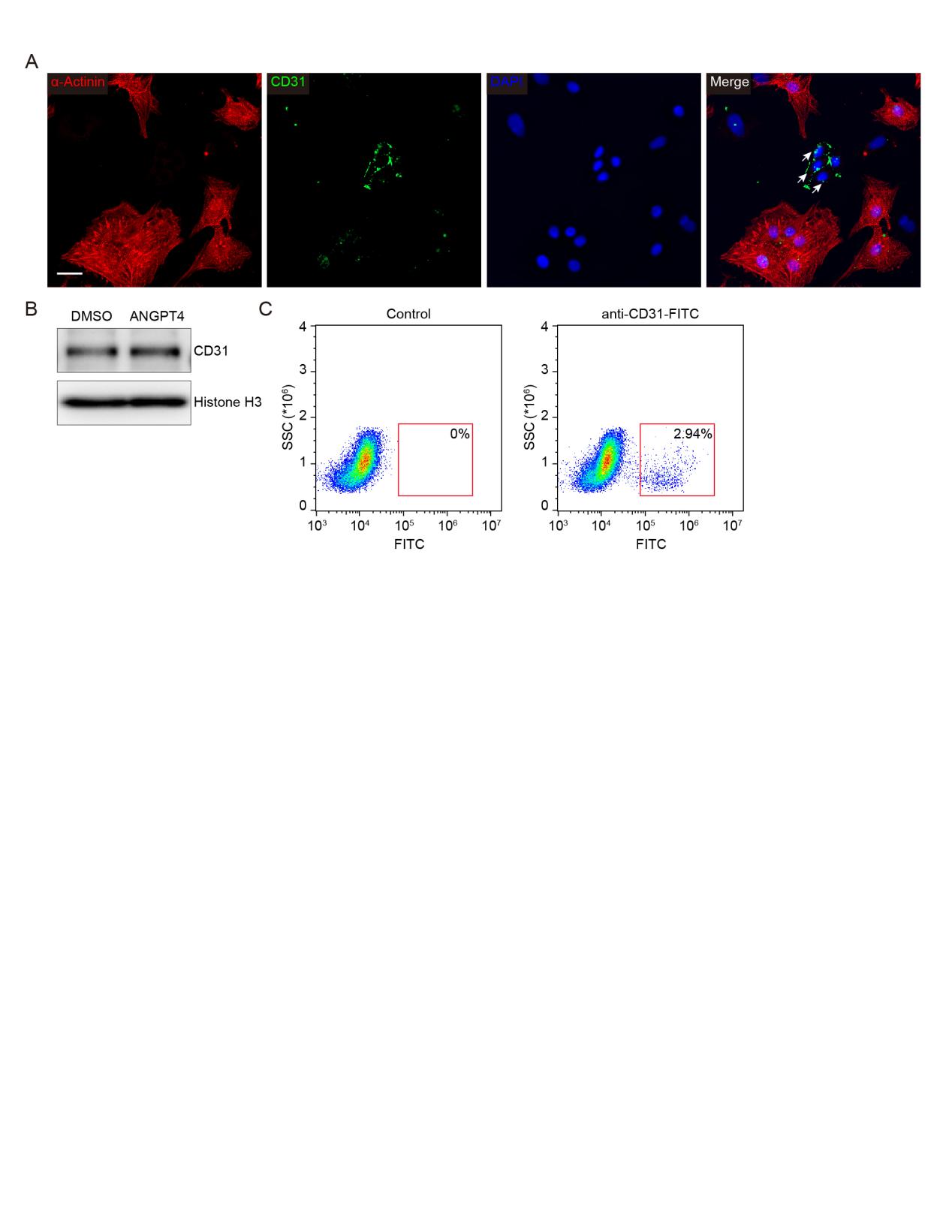


**Figure S11. *in vitro* culture of neonatal rat cardiac cells**

**(A)** Immunostaining of anti-α-actinin (red) and anti-CD31 (green), showing co-culture system of neonatal rat cardiomyocytes and endocardial cells. Arrows indicate CD31-positive cells. Scale bar, 25 μm.

**(B)** Western blot result showed existence of CD31-positive endocardial cells in cultured neonatal rat cardiac cells.

**(C)** FACS analysis result showed the percentage of CD31-positive endocardial cells after 3 days of *in vitro* culture of neonatal rat cardiac cells. Cells without anti-CD31-FITC incubation were used as negative control.
