# Supplementary material for: Single-cell analysis reveals an Angpt4-initiated EPDC-EC-CM cellular coordination cascade during heart regeneration
Source: Protein Cell. 2022 May 18;14(5):350–68. doi: 10.1093/procel/pwac010 (PMC10166170; doi:10.1093/procel/pwac010)
Supplement: pwac010_suppl_Supplementary_Table_S1 [file pwac010_suppl_supplementary_table_s1.pdf]

Table S1: Sample information

Batch	Stage	Condition	Sample name	Barcode	Cell number	Cell collection	Breed
B01	1 dpt	MTZ	B01_1dpt_MTZ_V1	1-48	48	pipette	*Tg(vmhc:mCherry-NTR; amhc:EGFP)*
B01	1 dpt	MTZ	B01_1dpt_MTZ_V2	49-96	48	pipette	*Tg(vmhc:mCherry-NTR; amhc:EGFP)*
B01	1 dpt	CT	B01_1dpt_CT_V1	33-64	32	pipette	*Tg(vmhc:mCherry-NTR; amhc:EGFP)*
B02	1 dpt	MTZ	B02_1dpt_MTZ_AV1	1-48	48	pipette	*Tg(vmhc:mCherry-NTR; amhc:EGFP)*
B02	1 dpt	MTZ	B02_1dpt_MTZ_AV2	49-96	48	pipette	*Tg(vmhc:mCherry-NTR; amhc:EGFP)*
B02	1 dpt	CT	B02_1dpt_CT_AV3	1-48	48	pipette	*Tg(vmhc:mCherry-NTR; amhc:EGFP)*
B02	1 dpt	CT	B02_1dpt_CT_AV4	49-96	48	pipette	*Tg(vmhc:mCherry-NTR; amhc:EGFP)*
B03	4 dpt	MTZ	B03_4dpt_MTZ_AV1	1-96	96	pipette	*Tg(vmhc:mCherry-NTR; amhc:EGFP)*
B03	4 dpt	CT	B03_4dpt_CT_AV1	1-96	96	pipette	*Tg(vmhc:mCherry-NTR; amhc:EGFP)*
B04	2 dpt	CT	B04_2dpt_CT_AV1	1-96	96	pipette	*Tg(vmhc:mCherry-NTR; amhc:EGFP)*
B04	2 dpt	CT	B04_2dpt_CT_AV2	1-48	48	pipette	*Tg(vmhc:mCherry-NTR; amhc:EGFP)*
B04	2 dpt	MTZ	B04_2dpt_MTZ_AV1	1-96	96	pipette	*Tg(vmhc:mCherry-NTR; amhc:EGFP)*
B04	2 dpt	MTZ	B04_2dpt_MTZ_AV2	49-96	48	pipette	*Tg(vmhc:mCherry-NTR; amhc:EGFP)*
B05	3 dpt	CT	B05_3dpt_CT_AV1	1-96	96	pipette	*Tg(vmhc:mCherry-NTR; amhc:EGFP)*
B05	3 dpt	CT	B05_3dpt_CT_AV2	49-96	48	pipette	*Tg(vmhc:mCherry-NTR; amhc:EGFP)*
B05	3 dpt	MTZ	B05_3dpt_MTZ_AV1	1-96	96	pipette	*Tg(vmhc:mCherry-NTR; amhc:EGFP)*
B05	3 dpt	MTZ	B05_3dpt_MTZ_AV2	1-48	48	pipette	*Tg(vmhc:mCherry-NTR; amhc:EGFP)*
B06	1 dpt	CT	B06_1dpt_CT_CMA	49-96	48	FACS	*Tg(vmhc:mCherry-NTR; amhc:EGFP)*
B06	1 dpt	CT	B06_1dpt_CT_CMV	1-48	48	FACS	*Tg(vmhc:mCherry-NTR; amhc:EGFP)*
B06	1 dpt	MTZ	B06_1dpt_MTZ_CMA	1-96	96	FACS	*Tg(vmhc:mCherry-NTR; amhc:EGFP)*
B06	1 dpt	MTZ	B06_1dpt_MTZ_CMV	1-96	96	FACS	*Tg(vmhc:mCherry-NTR; amhc:EGFP)*
B06	2 dpt	CT	B06_2dpt_CT_CMA	1-48	48	FACS	*Tg(vmhc:mCherry-NTR; amhc:EGFP)*
B06	2 dpt	CT	B06_2dpt_CT_CMV	49-96	48	FACS	*Tg(vmhc:mCherry-NTR; amhc:EGFP)*
B06	2 dpt	MTZ	B06_2dpt_MTZ_CMA	1-96	96	FACS	*Tg(vmhc:mCherry-NTR; amhc:EGFP)*
B06	2 dpt	MTZ	B06_2dpt_MTZ_CMV	1-96	96	FACS	*Tg(vmhc:mCherry-NTR; amhc:EGFP)*
B07	3 dpt	CT	B07_3dpt_CT_CMV2	1-96	96	FACS	*Tg(vmhc:mCherry-NTR; amhc:EGFP)*
B07	3 dpt	CT	B07_3dpt_CT_CMA2	1-96	96	FACS	*Tg(vmhc:mCherry-NTR; amhc:EGFP)*
B06	3 dpt	MTZ	B06_3dpt_MTZ_CMA	1-96	96	FACS	*Tg(vmhc:mCherry-NTR; amhc:EGFP)*
B06	3 dpt	MTZ	B06_3dpt_MTZ_CMV	1-96	96	FACS	*Tg(vmhc:mCherry-NTR; amhc:EGFP)*
B06	4 dpt	CT	B06_4dpt_CT_CMA	49-96	48	FACS	*Tg(vmhc:mCherry-NTR; amhc:EGFP)*

B06	4 dpt	CT	B06_4dpt_CT_CMV	1-48	48	FACS	*Tg(vmhc:mCherry-NTR; amhc:EGFP)*
B06	4 dpt	MTZ	B06_4dpt_MTZ_CMA	1-96	96	FACS	*Tg(vmhc:mCherry-NTR; amhc:EGFP)*
B06	4 dpt	MTZ	B06_4dpt_MTZ_CMV	1-96	96	FACS	*Tg(vmhc:mCherry-NTR; amhc:EGFP)*
B08	1 dpt	ANG	B08_1dpt_ANG_CMA	1-96	96	FACS	*angpt4-/-; Tg(vmhc:mCherry-NTR; amhc:EGFP)*
B08	1 dpt	ANG	B08_1dpt_ANG_CMV1	1-96	96	FACS	*angpt4-/-; Tg(vmhc:mCherry-NTR; amhc:EGFP)*
B08	1 dpt	ANG	B08_1dpt_ANG_CMV2	1-96	96	FACS	*angpt4-/-; Tg(vmhc:mCherry-NTR; amhc:EGFP)*
B08	2 dpt	ANG	B08_2dpt_ANG_CMA	1-96	96	FACS	*angpt4-/-; Tg(vmhc:mCherry-NTR; amhc:EGFP)*
B08	2 dpt	ANG	B08_2dpt_ANG_CMV1	1-96	96	FACS	*angpt4-/-; Tg(vmhc:mCherry-NTR; amhc:EGFP)*
B08	2 dpt	ANG	B08_2dpt_ANG_CMV2	1-96	96	FACS	*angpt4-/-; Tg(vmhc:mCherry-NTR; amhc:EGFP)*
