# Supplementary material for: Single-cell analysis reveals an Angpt4-initiated EPDC-EC-CM cellular coordination cascade during heart regeneration
Source: Protein Cell. 2022 May 18;14(5):350–68. doi: 10.1093/procel/pwac010 (PMC10166170; doi:10.1093/procel/pwac010)
Supplement: pwac010_suppl_Supplementary_Table_S2 [file pwac010_suppl_supplementary_table_s2.pdf]

Table S2: Enriched GO terms		
**Table S2-1: Enriched GO terms of CT-CMA**		
**Description**	***p* value**	**Log(*p* value)**
striated muscle cell differentiation	4.13029E-10	-9.384019614
muscle cell differentiation	1.40531E-09	-8.85222771
muscle structure development	4.67281E-09	-8.330421989
striated muscle cell development	4.05974E-08	-7.391501932
muscle cell development	6.92982E-08	-7.15927809
myofibril assembly	2.23425E-07	-6.650868859
cellular component assembly involved in morphogenesis	3.45861E-07	-6.461097797
supramolecular fiber organization	1.33217E-06	-5.875439768
actin cytoskeleton organization	1.98199E-06	-5.702898854
actomyosin structure organization	2.54376E-06	-5.594524433
actin filament-based process	2.62586E-06	-5.580729091
sarcomere organization	5.30412E-06	-5.275386664
cellular component morphogenesis	0.000181816	-3.740367671
organelle assembly	0.006007557	-2.221302101
keratinization	6.02284E-09	-8.220198543
formation of the cornified envelope	6.02284E-09	-8.220198543
developmental Biology	0.000330393	-3.480969723
tissue regeneration	0.005418203	-2.266144766
cardiac muscle contraction	1.07205E-08	-7.969785398
adrenergic signaling in cardiomyocytes	0.000258763	-3.587097982
cardiac muscle tissue development	1.32428E-08	-7.878021555
striated muscle tissue development	1.27265E-07	-6.895291747
muscle tissue development	1.75344E-07	-6.756108546
cardiac atrium development	1.17843E-06	-5.928697987
cardiac chamber development	7.73895E-06	-5.1113178
cardiac muscle cell differentiation	1.73493E-05	-4.760717317
heart development	1.98986E-05	-4.7011767
cardiocyte differentiation	5.33304E-05	-4.273025021
cardiac chamber morphogenesis	5.92611E-05	-4.22723018
heart morphogenesis	0.004881209	-2.311472592
heart contraction	2.23123E-07	-6.651456284
heart process	2.66297E-07	-6.574633945
muscle contraction	8.83059E-07	-6.054010092
muscle system process	1.2458E-06	-5.904550704
regulation of muscle contraction	3.48815E-06	-5.457404871
regulation of muscle system process	5.30412E-06	-5.275386664
blood circulation	5.74854E-06	-5.240442727
circulatory system process	6.38648E-06	-5.194738228
regulation of system process	5.28011E-05	-4.277357003
striated muscle contraction	0.000587724	-3.230826494
regulation of heart contraction	0.001018079	-2.992218352
regulation of blood circulation	0.002033965	-2.691656586
calcium signaling pathway	0.006786093	-2.168380216
striated Muscle Contraction	6.85042E-07	-6.164282526
muscle contraction	8.63953E-06	-5.063509983

ATP metabolic process	0.000523676	-3.280937298
purine ribonucleoside triphosphate metabolic process	0.000804567	-3.094437884
purine nucleoside triphosphate metabolic process	0.000845896	-3.072683177
ribonucleoside triphosphate metabolic process	0.001002378	-2.998968516
purine nucleoside monophosphate metabolic process	0.001125927	-2.948489691
purine ribonucleoside monophosphate metabolic process	0.001125927	-2.948489691
nucleoside triphosphate metabolic process	0.001345278	-2.871187909
ribonucleoside monophosphate metabolic process	0.001496625	-2.824886927
nucleoside monophosphate metabolic process	0.001625966	-2.788888552
generation of precursor metabolites and energy	0.002060475	-2.686032596
drug metabolic process	0.004724457	-2.325648101
oxidative phosphorylation	0.007105967	-2.148376842
purine ribonucleotide metabolic process	0.007803673	-2.107700955
ribonucleotide metabolic process	0.009456181	-2.024284208
purine nucleotide metabolic process	0.009789519	-2.009238667
muscle organ development	0.002139973	-2.669591662
skeletal muscle tissue development	0.005067306	-2.295222839
skeletal muscle organ development	0.006692011	-2.17444338
regulation of actin filament organization	0.008283164	-2.081803725

Table S2-2: Enriched GO terms of CT-CMV		
**Description**	***p* value**	**Log(*p* value)**
oxidative phosphorylation	2.83646E-27	-26.54722359
cardiac muscle contraction	3.15665E-20	-19.50077425
purine ribonucleoside triphosphate metabolic process	2.96996E-20	-19.52725005
purine nucleoside triphosphate metabolic process	3.80892E-20	-19.41919773
ribonucleoside triphosphate metabolic process	8.84943E-20	-19.05308475
ATP metabolic process	1.32046E-19	-18.87927511
nucleoside triphosphate metabolic process	3.81681E-19	-18.41829955
generation of precursor metabolites and energy	3.18176E-18	-17.49733207
purine nucleoside monophosphate metabolic process	4.69069E-18	-17.32876339
purine ribonucleoside monophosphate metabolic process	4.69069E-18	-17.32876339
purine ribonucleotide metabolic process	7.5915E-18	-17.11967238
ribonucleoside monophosphate metabolic process	1.76977E-17	-16.75208214
ribonucleotide metabolic process	2.27344E-17	-16.64331603
nucleoside monophosphate metabolic process	2.60606E-17	-16.58401616
purine nucleotide metabolic process	2.77229E-17	-16.55716113
ribose phosphate metabolic process	5.28794E-17	-16.27671354
purine-containing compound metabolic process	2.27725E-16	-15.64258873
nucleotide metabolic process	2.61833E-16	-15.58197604
nucleoside phosphate metabolic process	3.06299E-16	-15.51385485
nucleobase-containing small molecule metabolic process	1.04985E-14	-13.97887272
purine nucleoside triphosphate biosynthetic process	1.11313E-14	-13.95345543
purine ribonucleoside triphosphate biosynthetic process	1.11313E-14	-13.95345543
ribonucleoside triphosphate biosynthetic process	3.95588E-14	-13.40275645
drug metabolic process	6.62904E-14	-13.1785495
ATP biosynthetic process	8.18265E-14	-13.08710599
nucleoside triphosphate biosynthetic process	1.56943E-13	-12.80425795
purine ribonucleotide biosynthetic process	2.24753E-13	-12.64829517
organophosphate metabolic process	2.33166E-13	-12.63233553
purine nucleotide biosynthetic process	3.58129E-13	-12.44596068
ribonucleotide biosynthetic process	6.9852E-13	-12.15582099
purine-containing compound biosynthetic process	7.50732E-13	-12.124515
ribose phosphate biosynthetic process	9.29688E-13	-12.03166297
purine nucleoside monophosphate biosynthetic process	3.25838E-12	-11.48699813
purine ribonucleoside monophosphate biosynthetic process	3.25838E-12	-11.48699813
nucleotide biosynthetic process	1.1201E-11	-10.95074311
ribonucleoside monophosphate biosynthetic process	1.25064E-11	-10.90286611
nucleoside phosphate biosynthetic process	1.57664E-11	-10.80226713
nucleoside monophosphate biosynthetic process	1.66572E-11	-10.77839797
organophosphate biosynthetic process	2.93111E-11	-10.53296787
carbohydrate derivative metabolic process	1.19884E-09	-8.921240341
carbohydrate derivative biosynthetic process	2.01223E-07	-6.696323024
carbon metabolism	2.71994E-07	-6.565440291
nucleoside diphosphate phosphorylation	4.16278E-07	-6.380616766
nucleotide phosphorylation	4.62432E-07	-6.334951873
glycolysis, core module involving three-carbon compounds	5.87613E-07	-6.230908376
glycolysis (Embden-Meyerhof pathway), glucose => pyruvate	6.26285E-07	-6.203227926

glucose metabolism	7.31345E-07	-6.135877505
nucleoside diphosphate metabolic process	7.60713E-07	-6.118779426
carbohydrate catabolic process	1.20086E-06	-5.920507061
glycolytic process	1.68861E-06	-5.772471257
ATP generation from ADP	1.91549E-06	-5.717719531
pyruvate biosynthetic process	1.91549E-06	-5.717719531
pyridine nucleotide metabolic process	2.9168E-06	-5.53509383
nicotinamide nucleotide metabolic process	2.9168E-06	-5.53509383
ADP metabolic process	3.07352E-06	-5.512363783
gluconeogenesis, oxaloacetate => fructose-6P	3.41946E-06	-5.466042934
purine nucleoside diphosphate metabolic process	3.43464E-06	-5.464118812
purine ribonucleoside diphosphate metabolic process	3.43464E-06	-5.464118812
ribonucleoside diphosphate metabolic process	3.82826E-06	-5.416999122
pyridine-containing compound metabolic process	3.89712E-06	-5.409256164
glycolysis	4.1632E-06	-5.380572618
gluconeogenesis	5.02021E-06	-5.299278042
biosynthesis of amino acids	5.48332E-06	-5.260956373
pyruvate metabolic process	6.35843E-06	-5.196650111
oxidoreduction coenzyme metabolic process	1.08441E-05	-4.964804619
nicotinamide nucleotide biosynthetic process	1.09459E-05	-4.960750423
pyridine nucleotide biosynthetic process	1.09459E-05	-4.960750423
nucleobase-containing small molecule biosynthetic process	1.36155E-05	-4.865965995
pyridine-containing compound biosynthetic process	1.52141E-05	-4.817753602
nucleotide catabolic process	1.64601E-05	-4.783567882
nucleoside phosphate catabolic process	2.06803E-05	-4.684443444
cofactor biosynthetic process	2.3679E-05	-4.625635794
glycolysis / gluconeogenesis	3.85356E-05	-4.414138407
monocarboxylic acid biosynthetic process	7.1027E-05	-4.14857649
coenzyme biosynthetic process	8.65156E-05	-4.062905604
metabolism of carbohydrates	0.0001126	-3.948462012
coenzyme metabolic process	0.00012905	-3.889241917
organophosphate catabolic process	0.000248076	-3.60541556
cofactor metabolic process	0.00026038	-3.584391925
carbohydrate metabolic process	0.000351103	-3.454565347
monocarboxylic acid metabolic process	0.000364884	-3.437845216
carboxylic acid metabolic process	0.000469547	-3.328321284
oxoacid metabolic process	0.000653129	-3.185001145
carboxylic acid biosynthetic process	0.000756442	-3.121224226
organic acid biosynthetic process	0.000793981	-3.100190143
organic acid metabolic process	0.001102665	-2.957556299
small molecule biosynthetic process	0.003458748	-2.4610811
nucleobase-containing compound catabolic process	0.006217304	-2.206397915
RNA degradation	0.009440048	-2.025025779
respiratory electron transport, ATP synthesis by chemiosmotic couplin	4.26312E-14	-13.37027253
the citric acid (TCA) cycle and respiratory electron transport	6.87226E-12	-11.16290039
respiratory electron transport	1.95699E-10	-9.708410316
complex I biogenesis	1.48343E-06	-5.82873345
muscle structure development	2.77814E-13	-12.55624647

muscle tissue development	7.94441E-11	-10.09993819
striated muscle tissue development	6.93094E-10	-9.159207627
skeletal muscle organ development	4.33227E-07	-6.363284107
muscle organ development	5.71612E-07	-6.242898859
skeletal muscle tissue development	2.94639E-06	-5.530709571
myotube differentiation	4.6577E-05	-4.331828208
myotube cell development	0.000239676	-3.620374846
skeletal muscle fiber development	0.000239676	-3.620374846
muscle fiber development	0.000826779	-3.082610792
heart contraction	1.51766E-11	-10.81882462
heart process	2.06319E-11	-10.68546163
blood circulation	3.3369E-10	-9.476657369
circulatory system process	4.06655E-10	-9.390773982
muscle contraction	3.66953E-08	-7.435389614
cardiac muscle contraction	7.40182E-08	-7.130661295
striated muscle contraction	4.62432E-07	-6.334951873
regulation of heart contraction	1.42693E-06	-5.845598265
muscle contraction	1.99042E-06	-5.701055138
muscle system process	2.94639E-06	-5.530709571
regulation of blood circulation	5.85596E-06	-5.232401875
striated muscle contraction	7.11648E-06	-5.147734793
regulation of system process	8.36374E-06	-5.077599346
skeletal muscle contraction	0.000414584	-3.382387475
multicellular organismal movement	0.000556629	-3.254434334
musculoskeletal movement	0.000556629	-3.254434334
regulation of muscle contraction	0.000856206	-3.067421627
regulation of muscle system process	0.001157655	-2.936420702
muscle cell development	1.74831E-11	-10.75738112
striated muscle cell development	1.25675E-10	-9.900751742
muscle cell differentiation	3.0187E-10	-9.520179836
myofibril assembly	8.88554E-10	-9.051315983
striated muscle cell differentiation	9.06199E-10	-9.042776569
sarcomere organization	1.30441E-09	-8.88458526
cellular component assembly involved in morphogenesis	1.72016E-09	-8.764430991
actin cytoskeleton organization	9.23195E-09	-8.034706496
actin filament-based process	1.43634E-08	-7.842742433
actomyosin structure organization	3.45323E-08	-7.461774816
ventricular cardiac myofibril assembly	4.97377E-07	-6.303313954
ventricular cardiac muscle cell development	6.86176E-06	-5.163564771
cardiac muscle tissue development	1.21661E-05	-4.914847724
ventricular cardiac muscle cell differentiation	1.45962E-05	-4.835759033
cellular component morphogenesis	7.39348E-05	-4.13115109
cardiac myofibril assembly	8.06163E-05	-4.093577286
supramolecular fiber organization	0.000247454	-3.606504963
cardiac muscle cell development	0.00092621	-3.033290395
cardiac cell development	0.001157655	-2.936420702
cardiac muscle cell differentiation	0.002707525	-2.567427535
organelle assembly	0.002856489	-2.544167431

cardiocyte differentiation	0.00601311	-2.220900859
oxidative phosphorylation	2.72783E-11	-10.56418317
cellular respiration	6.58197E-10	-9.181644074
energy derivation by oxidation of organic compounds	7.85713E-10	-9.104735803
mitochondrial ATP synthesis coupled electron transport	6.39489E-08	-7.194167188
ATP synthesis coupled electron transport	1.2827E-07	-6.891875111
aerobic respiration	1.657E-07	-6.780676432
respiratory electron transport chain	3.73971E-07	-6.427161614
electron transport chain	5.67365E-07	-6.246137536
mitochondrial electron transport, cytochrome c to oxygen	1.99963E-05	-4.699049588
aerobic electron transport chain	1.99963E-05	-4.699049588
oxidation-reduction process	2.07592E-05	-4.68279014
heart development	2.27577E-10	-9.642872345
embryonic heart tube development	0.00030304	-3.518499524
heart morphogenesis	0.001455528	-2.836979386
embryonic organ development	0.00396689	-2.401549888
energy coupled proton transport, down electrochemical gradient	6.10424E-09	-8.21436856
ATP synthesis coupled proton transport	6.10424E-09	-8.21436856
mitochondrial biogenesis	9.24043E-08	-7.034307663
proton transmembrane transport	3.89712E-06	-5.409256164
mitochondrial ATP synthesis coupled proton transport	1.45962E-05	-4.835759033
formation of ATP by chemiosmotic coupling	5.43386E-05	-4.264891887
cristae formation	5.43386E-05	-4.264891887
organelle biogenesis and maintenance	0.000329401	-3.482275472
inorganic cation transmembrane transport	0.002740939	-2.562100694
cation transmembrane transport	0.004901014	-2.309714056
inorganic ion transmembrane transport	0.005013228	-2.299882543
mitochondrial transmembrane transport	0.006723218	-2.172422792
dicarboxylic acid metabolic process	0.00124217	-2.905818991
pyruvate metabolism	0.001518709	-2.81852537
cysteine and methionine metabolism	0.001943353	-2.71144827
arginine and proline metabolism	0.00330662	-2.480615737
adrenergic signaling in cardiomyocytes	0.003604029	-2.443211674
cardiac conduction	0.005139366	-2.289090447
PPAR signaling pathway	0.005139366	-2.289090447
focal adhesion	0.007409751	-2.130196384

Table S2-3: Enriched GO terms of CT-EC		
**Description**	***p* value**	**Log(*p* value)**
vasculature development	1.9589E-10	-9.707987157
cardiovascular system development	1.9589E-10	-9.707987157
blood vessel development	1.89054E-09	-8.723414907
blood vessel morphogenesis	3.49651E-09	-8.456364985
tube morphogenesis	1.56296E-07	-6.806051546
angiogenesis	2.97434E-07	-6.526609877
sprouting angiogenesis	2.28232E-05	-4.641622797
endothelial cell proliferation	0.000593266	-3.226750579
epithelial cell proliferation	0.002427503	-2.614840214
regulation of cell-cell adhesion	0.006470416	-2.189067818
cell adhesion	0.009304451	-2.031309253
biological adhesion	0.009304451	-2.031309253
wound healing	5.79297E-08	-7.237099069
response to wounding	3.29478E-07	-6.482174009
developmental growth	0.000171695	-3.765242248
tissue regeneration	0.000248294	-3.605034585
growth	0.000309218	-3.509734693
fin regeneration	0.000386413	-3.412948421
regeneration	0.001486342	-2.827881304
Notch signaling pathway	0.000129167	-3.888848128
lymph vessel development	0.000167555	-3.775843447
artery development	0.000593266	-3.226750579
regulation of Notch signaling pathway	0.002095626	-2.678686313
platelet degranulation	1.60236E-05	-4.795240077
response to elevated platelet cytosolic Ca2+	1.83573E-05	-4.73619055
hemostasis	5.67713E-05	-4.245870977
platelet activation, signaling and aggregation	0.000520659	-3.283446411
RAF/MAP kinase cascade	0.006064591	-2.217198504
MAPK1/MAPK3 signaling	0.006599452	-2.180492136
FLT3 Signaling	0.006878256	-2.162521641
MAPK family signaling cascades	0.009050391	-2.043332674
negative regulation of cell fate commitment	3.72841E-05	-4.428476748
negative regulation of multicellular organismal process	3.94236E-05	-4.404243804
negative regulation of developmental process	0.000181788	-3.740434067
mesodermal cell differentiation	0.000230614	-3.637113537
negative regulation of cell differentiation	0.000239269	-3.62111375
regulation of cell fate commitment	0.00025746	-3.589289851
formation of primary germ layer	0.000349406	-3.456669076
tissue morphogenesis	0.001000427	-2.9998147
mesoderm formation	0.001274904	-2.89452242
mesoderm morphogenesis	0.00199168	-2.700780368
cardiocyte differentiation	0.00423296	-2.373355842
regulation of multicellular organismal development	0.004842579	-2.314923251
muscle structure development	0.005763736	-2.239295894
regulation of cell differentiation	0.008455129	-2.072879779
blood coagulation	0.000213573	-3.670454496

hemostasis	0.000226368	-3.645185876
coagulation	0.000253599	-3.595852004
regulation of body fluid levels	0.00049108	-3.308847787
transmembrane receptor protein tyrosine kinase signaling pathway	0.000570046	-3.244089735
cell proliferation	0.00065366	-3.184648327
enzyme linked receptor protein signaling pathway	0.002524207	-2.597875113
regulation of cell proliferation	0.003337124	-2.476627676
peptidyl-tyrosine phosphorylation	0.00585713	-2.232315161
peptidyl-tyrosine modification	0.006470416	-2.189067818
negative regulation of signal transduction	0.002006955	-2.697462386
negative regulation of cell communication	0.002502171	-2.601682969
negative regulation of signaling	0.002502171	-2.601682969
negative regulation of response to stimulus	0.006119503	-2.213283855
negative regulation of Wnt signaling pathway	0.007574887	-2.120623826
negative regulation of proteolysis	0.003761283	-2.424663983
regulation of catalytic activity	0.004074842	-2.389889237
negative regulation of catalytic activity	0.008229452	-2.084629092
cellular response to xenobiotic stimulus	0.006470416	-2.189067818
response to xenobiotic stimulus	0.009038962	-2.043881441
biological oxidations	0.009561191	-2.019487998
eye development	0.007877997	-2.103584181
visual system development	0.007877997	-2.103584181

Table S2-4: Enriched GO terms of CT-EP		
**Description**	***p* value**	**Log(*p* value)**
ECM-receptor interaction	6.55805E-08	-7.183224992
focal adhesion	1.05218E-05	-4.977908966
AGE-RAGE signaling pathway in diabetic complications	0.000209545	-3.67872182
extracellular matrix organization	0.007146547	-2.145903762
extracellular structure organization	0.008040062	-2.0947406
response to wounding	2.85164E-06	-5.544905791
wound healing	5.82371E-05	-4.234800052
tissue remodeling	0.000136372	-3.865274055
regeneration	0.000174918	-3.757165001
tissue regeneration	0.000234426	-3.629993465
pectoral fin development	0.002818263	-2.55001853
fin regeneration	0.004749794	-2.323325234
fin development	0.009769094	-2.010145724
platelet degranulation	1.50915E-05	-4.821267575
response to elevated platelet cytosolic Ca2+	1.7291E-05	-4.762179006
hemostasis	0.000353085	-3.452121068
platelet activation, signaling and aggregation	0.000492061	-3.30798088
neutrophil degranulation	0.000529156	-3.27641628
innate immune system	0.001626556	-2.788731015
integrin cell surface interactions	1.64117E-05	-4.784845302
degradation of the extracellular matrix	0.000107328	-3.969287642
ECM proteoglycans	0.000198312	-3.702651684
extracellular matrix organization	0.000280818	-3.551574555
signaling by PDGF	0.000485177	-3.314099634
developmental biology	0.002485656	-2.604559012
tube morphogenesis	2.58105E-05	-4.588204181
vasculature development	0.000127619	-3.894084912
cardiovascular system development	0.000127619	-3.894084912
blood vessel development	0.000205031	-3.688180788
blood vessel morphogenesis	0.000454268	-3.342688324
angiogenesis	0.00071783	-3.143978574
vasculogenesis	0.001825668	-2.738578168
regulation of angiogenesis	0.008991591	-2.046163437
negative regulation of multicellular organismal process	3.64031E-05	-4.438861897
negative regulation of developmental process	0.000169896	-3.76981695
regulation of multicellular organismal development	0.001255683	-2.901119862
negative regulation of cell differentiation	0.002251653	-2.647498478
antimicrobial humoral response	4.40906E-05	-4.355653924
response to external biotic stimulus	0.000491127	-3.30880634
response to other organism	0.000491127	-3.30880634
response to biotic stimulus	0.000529156	-3.27641628
humoral immune response	0.000537202	-3.269862768
multi-organism process	0.006791483	-2.168035362
immune response	0.009914336	-2.003736385
glomerulus morphogenesis	5.33688E-05	-4.272712419
glomerulus development	0.000140606	-3.851994884

kidney morphogenesis	0.000444997	-3.351642639
nephron development	0.000537202	-3.269862768
morphogenesis of an epithelium	0.001257818	-2.900382055
kidney development	0.001341803	-2.872311166
renal system development	0.001341803	-2.872311166
embryonic morphogenesis	0.001463502	-2.834606685
urogenital system development	0.001474762	-2.831278133
nephron epithelium development	0.001640512	-2.785020529
kidney epithelium development	0.002818263	-2.55001853
epithelial tube formation	0.003075895	-2.512028564
tissue morphogenesis	0.003675299	-2.434707346
tube formation	0.003781868	-2.422293675
epithelial tube morphogenesis	0.00560859	-2.25114634
embryonic organ development	0.005709856	-2.243374829
sensory organ morphogenesis	0.00985181	-2.006483993
cellular response to lipid	0.000333384	-3.477055155
cell motility	0.000523698	-3.280919317
localization of cell	0.000523698	-3.280919317
response to lipid	0.000701049	-3.154251838
regulation of response to external stimulus	0.000805361	-3.094009356
regulation of immune effector process	0.000954213	-3.020354724
cell migration	0.001095747	-2.960289735
response to cytokine	0.001102725	-2.957532885
cellular response to organic cyclic compound	0.001230503	-2.909917246
peptide ligand-binding receptors	0.00131727	-2.880325324
regulation of inflammatory response	0.001385639	-2.858349889
regulation of defense response	0.001457528	-2.836383176
inflammatory response	0.001654332	-2.781377355
negative regulation of cell-cell adhesion	0.001825668	-2.738578168
cell chemotaxis	0.002123306	-2.672987482
cell adhesion	0.002496041	-2.602748366
biological adhesion	0.002496041	-2.602748366
G alpha (i) signalling events	0.002640377	-2.578334048
regulation of cell adhesion	0.002974393	-2.526601642
negative regulation of cell adhesion	0.003347555	-2.475272263
GPCR ligand binding	0.003392606	-2.469466634
defense response	0.003478085	-2.458659779
granulocyte chemotaxis	0.004924291	-2.307656285
regulation of cell-cell adhesion	0.006254283	-2.203822498
immune effector process	0.007146547	-2.145903762
GPCR downstream signalling	0.007343911	-2.134072612
Signaling by GPCR	0.008078648	-2.092661322
leukocyte chemotaxis	0.008494224	-2.070876315
chemotaxis	0.008663529	-2.062305166
granulocyte migration	0.009246551	-2.034020221
regulation of response to stress	0.009454734	-2.024350683
Class A/1 (Rhodopsin-like receptors)	0.009687122	-2.013805229
liver development	0.00043414	-3.362370443

hepaticobiliary system development	0.000492061	-3.30798088
gland development	0.000769168	-3.113978557
anterior/posterior pattern specification	0.000769168	-3.113978557
somitogenesis	0.001272697	-2.895275066
regionalization	0.002091316	-2.679580241
segmentation	0.002123306	-2.672987482
somite development	0.004756723	-2.322692111
chordate embryonic development	0.008080952	-2.092537455
embryo development ending in birth or egg hatching	0.008243792	-2.083872985
hematopoietic or lymphoid organ development	0.001818287	-2.740337668
immune system development	0.002076501	-2.682667939
RIG-I-like receptor signaling pathway	0.002574412	-2.589321879
NOD-like receptor signaling pathway	0.004756723	-2.322692111
hemopoiesis	0.006307852	-2.200118531
Toll-like receptor signaling pathway	0.008991591	-2.046163437
coagulation	0.003488721	-2.457333773
organic hydroxy compound transport	0.005470856	-2.261944751
lipid transport	0.00758462	-2.120066162

Table S2-5: Enriched GO terms of CT-EPDC		
**Description**	***p* value**	**Log(*p* value)**
extracellular matrix organization	6.77372E-08	-7.169173054
extracellular structure organization	9.0784E-08	-7.041990577
collagen fibril organization	0.000222394	-3.652876153
extracellular matrix organization	0.000280818	-3.551574555
collagen formation	0.000619446	-3.207996443
focal adhesion	9.8201E-08	-7.007884196
regulation of actin cytoskeleton	0.006074283	-2.216505
supramolecular fiber organization	2.19087E-06	-5.659383326
muscle contraction	0.000159319	-3.797732508
muscle system process	0.000209545	-3.67872182
actin filament-based process	0.000676134	-3.169967488
actin cytoskeleton organization	0.002335622	-2.631597356
actin filament organization	0.002776578	-2.556490133
skeletal system development	0.00019796	-3.703421617
cartilage development	0.005323414	-2.273809769
connective tissue development	0.006190326	-2.208286468
AGE-RAGE signaling pathway in diabetic complications	0.000209545	-3.67872182
ECM-receptor interaction	0.000447324	-3.349378284
notochord development	0.000285018	-3.545127726
embryonic organ development	0.005709856	-2.243374829
sensory organ morphogenesis	0.000325651	-3.487248029
eye morphogenesis	0.000343738	-3.463772244
camera-type eye morphogenesis	0.000873269	-3.058852021
mesenchyme development	0.003206799	-2.493928234
ameboidal-type cell migration	0.004516872	-2.345162214
mesenchymal cell differentiation	0.006588979	-2.181181884
vascular smooth muscle contraction	0.000492061	-3.30798088
apelin signaling pathway	0.008040062	-2.0947406
heart contraction	0.000555622	-3.25522075
heart process	0.000625167	-3.204004163
blood circulation	0.000769168	-3.113978557
circulatory system process	0.000836141	-3.077720564
developmental biology	0.002485656	-2.604559012
axon guidance	0.004756723	-2.322692111
Nervous system development	0.004866644	-2.312770383
salmonella infection	0.008991591	-2.046163437
wound healing	0.003087087	-2.5104512
response to wounding	0.006771194	-2.169334739
cell adhesion	0.008661712	-2.062396271
biological adhesion	0.008661712	-2.062396271
regeneration	0.009341557	-2.029580749
platelet degranulation	0.003781868	-2.422293675
response to elevated platelet cytosolic Ca2+	0.00408962	-2.388317014
platelet activation, signaling and aggregation	0.004132156	-2.383823245
chordate embryonic development	0.008080952	-2.092537455
embryo development ending in birth or egg hatching	0.008243792	-2.083872985

Table S2-6: Enriched GO terms of MTZ-CMA		
**Description**	***p* value**	**Log(*p* value)**
muscle structure development	4.27468E-22	-21.3690967
muscle tissue development	1.31962E-21	-20.87955219
muscle cell differentiation	1.28147E-20	-19.89229276
striated muscle tissue development	1.54971E-20	-19.80974976
heart development	3.75733E-20	-19.42512099
muscle cell development	2.47615E-18	-17.6062235
striated muscle cell differentiation	1.56429E-17	-16.80568311
striated muscle cell development	1.92426E-17	-16.71573613
cardiac muscle tissue development	5.64701E-17	-16.24818159
myofibril assembly	6.04291E-16	-15.21875398
cellular component assembly involved in morphogenesis	1.62912E-15	-14.78804666
actomyosin structure organization	1.4663E-13	-12.83377628
actin cytoskeleton organization	8.88697E-12	-11.0512463
actin filament-based process	1.5311E-11	-10.81499563
sarcomere organization	1.98831E-11	-10.7015162
cardiac muscle cell differentiation	2.39676E-10	-9.620375451
supramolecular fiber organization	3.58525E-10	-9.445480201
cardiac muscle cell development	5.87212E-10	-9.231205251
cardiac cell development	1.03374E-09	-8.985588906
cardiocyte differentiation	2.46364E-09	-8.608421982
cardiac myofibril assembly	1.47422E-08	-7.831438476
cellular component morphogenesis	7.50145E-08	-7.124854868
ventricular cardiac myofibril assembly	4.50342E-07	-6.346457655
organelle assembly	9.33999E-07	-6.029653506
ventricular cardiac muscle cell development	6.21591E-06	-5.206495593
ventricular cardiac muscle cell differentiation	1.32256E-05	-4.878583557
heart process	4.18158E-20	-19.37865971
circulatory system process	5.01768E-19	-18.2994969
heart contraction	8.60915E-19	-18.0650398
blood circulation	8.17302E-18	-17.08761759
muscle contraction	1.05056E-11	-10.97857929
muscle system process	2.00344E-11	-10.69822348
regulation of heart contraction	1.75093E-09	-8.756731621
regulation of system process	2.09928E-09	-8.677928701
regulation of blood circulation	1.21478E-08	-7.915503302
cardiac muscle contraction	6.07732E-08	-7.216287651
striated muscle contraction	3.80479E-07	-6.419669395
regulation of muscle contraction	3.11765E-05	-4.506173293
regulation of muscle system process	4.70964E-05	-4.327012431
muscle contraction	5.09439E-11	-10.29290745
striated muscle contraction	1.22735E-07	-6.911032689
muscle organ development	1.67753E-10	-9.775329303
skeletal muscle tissue development	8.84513E-09	-8.053295926
skeletal muscle organ development	2.13642E-08	-7.670312356
muscle fiber development	5.36693E-05	-4.270274083
myotube cell development	0.000211282	-3.675136493

skeletal muscle fiber development	0.000211282	-3.675136493
myotube differentiation	0.000578046	-3.238037691
cardiac muscle contraction	1.16818E-09	-8.932490046
adrenergic signaling in cardiomyocytes	6.32442E-07	-6.19897962
atrioventricular canal development	4.82499E-09	-8.316504006
cardiac muscle cell proliferation	4.92662E-05	-4.307451082
striated muscle cell proliferation	7.31088E-05	-4.136030516
cardiac muscle tissue growth	7.31088E-05	-4.136030516
heart growth	0.00012127	-3.91624772
muscle cell proliferation	0.000162665	-3.788705754
cardiac conduction	0.000362242	-3.441001511
organ growth	0.000778358	-3.108820528
heart morphogenesis	2.80835E-08	-7.551548873
embryonic heart tube development	0.001899691	-2.72131701
tissue morphogenesis	0.001937372	-2.712786897
retina morphogenesis in camera-type eye	0.00229195	-2.639794902
heart looping	0.004575377	-2.339573102
mesenchyme development	0.00503454	-2.298040203
embryonic heart tube morphogenesis	0.006439691	-2.191134993
epithelial tube morphogenesis	0.008696105	-2.060675244
camera-type eye morphogenesis	0.009307842	-2.031150977
retina development in camera-type eye	0.009692134	-2.013580606
oxidative phosphorylation	3.97082E-07	-6.401119902
complex I biogenesis	3.85379E-05	-4.414112432
respiratory electron transport	0.000120846	-3.917769127
the citric acid (TCA) cycle and respiratory electron transport	0.000187224	-3.727638497
respiratory electron transport, ATP synthesis by chemiosmotic couplin	0.000362242	-3.441001511
cardiac chamber development	2.61327E-06	-5.582815093
cardiac atrium development	6.21591E-06	-5.206495593
cardiac ventricle development	6.24504E-06	-5.204464594
cardiac ventricle morphogenesis	7.31088E-05	-4.136030516
cardiac chamber morphogenesis	0.000303543	-3.517780003
ATP metabolic process	4.60943E-05	-4.336352781
purine ribonucleoside triphosphate metabolic process	8.71284E-05	-4.059840255
purine nucleoside triphosphate metabolic process	9.38325E-05	-4.027646607
ribonucleoside triphosphate metabolic process	0.000120598	-3.918658605
purine nucleoside monophosphate metabolic process	0.000143181	-3.844114788
purine ribonucleoside monophosphate metabolic process	0.000143181	-3.844114788
nucleoside triphosphate metabolic process	0.000186163	-3.730106712
ribonucleoside monophosphate metabolic process	0.000217817	-3.661907231
nucleoside monophosphate metabolic process	0.000246077	-3.60892956
generation of precursor metabolites and energy	0.000348504	-3.457791824
purine ribonucleotide metabolic process	0.000426221	-3.370365498
ribonucleotide metabolic process	0.000594654	-3.225735688
purine nucleotide metabolic process	0.000631435	-3.199671169
ribose phosphate metabolic process	0.000767296	-3.115036923
purine-containing compound metabolic process	0.001188689	-2.924931864
nucleotide metabolic process	0.002716085	-2.566056689

nucleoside phosphate metabolic process	0.002836282	-2.547250607
drug metabolic process	0.002960531	-2.528630455
oxidative phosphorylation	0.003630305	-2.440056832
carbohydrate catabolic process	0.004501879	-2.346606218
ATP biosynthetic process	0.005490008	-2.260427056
organophosphate metabolic process	0.005811563	-2.23570703
glycolysis / gluconeogenesis	0.005918813	-2.227765405
nucleobase-containing small molecule metabolic process	0.007398035	-2.130883628
purine nucleoside triphosphate biosynthetic process	0.008093268	-2.091876061
purine ribonucleoside triphosphate biosynthetic process	0.008093268	-2.091876061
organophosphate biosynthetic process	0.008215786	-2.085350871
inorganic cation transmembrane transport	0.000105632	-3.976206144
cation transmembrane transport	0.000230984	-3.636417825
inorganic ion transmembrane transport	0.000238165	-3.623122772
monovalent inorganic cation transport	0.000424248	-3.372379946
ion transmembrane transport	0.000455334	-3.341669866
cation transport	0.000546813	-3.262161099
metal ion transport	0.001018745	-2.991934604
sodium ion transmembrane transport	0.00229195	-2.639794902
sodium ion transport	0.005863264	-2.231860578
calcium signaling pathway	0.000334429	-3.475696312
regulation of actin cytoskeleton	0.001835858	-2.736160837
actin filament capping	0.000909062	-3.041406279
regulation of actin filament depolymerization	0.000979311	-3.009079249
negative regulation of actin filament depolymerization	0.000979311	-3.009079249
negative regulation of protein depolymerization	0.001381958	-2.859505288
actin filament depolymerization	0.001666752	-2.778129132
negative regulation of protein complex disassembly	0.001769215	-2.752219295
regulation of protein depolymerization	0.002099934	-2.677794285
negative regulation of actin filament polymerization	0.002340352	-2.630718811
negative regulation of protein polymerization	0.002340352	-2.630718811
negative regulation of supramolecular fiber organization	0.003630305	-2.440056832
negative regulation of protein complex assembly	0.003795506	-2.420730329
regulation of protein complex disassembly	0.004318377	-2.364679425
actin filament organization	0.004683859	-2.329396233
negative regulation of cytoskeleton organization	0.004690044	-2.328823088
protein depolymerization	0.005701983	-2.243974091
regulation of actin filament organization	0.005863264	-2.231860578
regulation of supramolecular fiber organization	0.009307842	-2.031150977

Table S2-7: Enriched GO terms of MTZ-CMV		
**Description**	***p* value**	**Log(*p* value)**
muscle contraction	1.2915E-19	-18.88890555
muscle system process	3.22783E-19	-18.49109001
striated muscle contraction	1.80931E-18	-17.74248597
skeletal muscle contraction	4.48545E-18	-17.34819399
multicellular organismal movement	1.43227E-17	-16.84397388
musculoskeletal movement	1.43227E-17	-16.84397388
cardiac muscle contraction	1.9808E-10	-9.703159851
regulation of muscle contraction	4.30577E-09	-8.365948947
regulation of muscle system process	8.23211E-09	-8.08448905
heart contraction	8.72423E-08	-7.059272878
heart process	1.06671E-07	-6.971954427
sarcomere organization	4.00191E-07	-6.39773262
blood circulation	3.46084E-06	-5.460818604
circulatory system process	3.89711E-06	-5.409257704
regulation of system process	1.51522E-05	-4.819524948
cardiac muscle contraction	1.47229E-15	-14.83200584
adrenergic signaling in cardiomyocytes	7.69206E-07	-6.11395716
supramolecular fiber organization	1.68985E-14	-13.77215085
actin cytoskeleton organization	3.74193E-14	-13.4269041
actin filament-based process	6.57528E-14	-13.18208595
striated muscle cell development	1.09237E-13	-12.96163105
muscle cell development	2.66123E-13	-12.57491705
striated muscle cell differentiation	9.87669E-13	-12.00538874
myofibril assembly	1.34736E-12	-11.87051749
cellular component assembly involved in morphogenesis	2.85306E-12	-11.54468852
muscle cell differentiation	4.89611E-12	-11.31014861
muscle structure development	5.29132E-11	-10.27643583
actomyosin structure organization	8.69449E-11	-10.06075567
actin filament organization	1.75891E-09	-8.754757612
myosin filament organization	3.51503E-08	-7.454070576
cellular component morphogenesis	2.21806E-06	-5.654026538
myosin filament assembly	3.98482E-06	-5.399591736
striated muscle myosin thick filament assembly	3.98482E-06	-5.399591736
skeletal myofibril assembly	5.25271E-05	-4.279616479
organelle assembly	5.53325E-05	-4.257019454
response to external biotic stimulus	1.69735E-09	-8.770229134
response to other organism	1.69735E-09	-8.770229134
response to biotic stimulus	1.95586E-09	-8.708662238
response to bacterium	8.75019E-09	-8.057982443
defense response	7.52073E-08	-7.123740257
defense response to other organism	1.80381E-07	-6.743808802
neutrophil chemotaxis	2.40435E-07	-6.619002342
multi-organism process	2.8721E-07	-6.541800696
defense response to bacterium	2.92381E-07	-6.534051239
granulocyte chemotaxis	4.22568E-07	-6.374103253
neutrophil migration	1.05621E-06	-5.976251547

leukocyte chemotaxis	1.36133E-06	-5.866036782
granulocyte migration	1.63455E-06	-5.786602063
myeloid leukocyte migration	3.38647E-06	-5.470252304
cell chemotaxis	6.13914E-06	-5.211892366
leukocyte migration	9.62392E-06	-5.016648026
immune response	2.8667E-05	-4.542617974
cellular response to interleukin-1	0.000289847	-3.537831512
response to interleukin-1	0.000400471	-3.397428418
response to wounding	0.000481208	-3.317667581
inflammatory response	0.00075136	-3.124152008
fin regeneration	0.002900346	-2.537550199
chemotaxis	0.003699512	-2.431855563
taxis	0.004521839	-2.344684859
cell migration	0.004555897	-2.341426118
cell motility	0.007174491	-2.144208881
localization of cell	0.007174491	-2.144208881
wound healing	0.009466309	-2.023819303
voluntary skeletal muscle contraction	2.53886E-09	-8.595360774
twitch skeletal muscle contraction	2.53886E-09	-8.595360774
slow-twitch skeletal muscle fiber contraction	4.79439E-07	-6.319266252
striated muscle contraction	2.96978E-08	-7.527275611
cardiac myofibril assembly	3.15817E-05	-4.500564205
muscle contraction	3.23758E-05	-4.489779186
cardiac muscle cell development	0.000370643	-3.43104428
cardiac cell development	0.000464663	-3.332861899
cardiac muscle tissue development	0.000530896	-3.274990916
cardiac muscle cell differentiation	0.001102138	-2.957764099
cardiocyte differentiation	0.002492064	-2.60344084
striated muscle tissue development	1.2607E-05	-4.899387372
muscle fiber development	1.37554E-05	-4.861527945
muscle tissue development	1.65215E-05	-4.78195118
muscle organ development	0.000734073	-3.134260988
skeletal muscle tissue development	0.001110864	-2.954338934
myotube cell development	0.001418793	-2.848081069
skeletal muscle fiber development	0.001418793	-2.848081069
skeletal muscle organ development	0.001609471	-2.793316971
myotube differentiation	0.003008453	-2.521656754
ATP synthesis coupled electron transport	2.69362E-05	-4.569663646
respiratory electron transport chain	5.40985E-05	-4.266814553
electron transport chain	7.10315E-05	-4.148548795
oxidative phosphorylation	8.60487E-05	-4.065255619
purine ribonucleoside triphosphate metabolic process	0.000217644	-3.662253375
purine nucleoside triphosphate metabolic process	0.000231647	-3.635173334
ribonucleoside triphosphate metabolic process	0.000286123	-3.543447389
cellular respiration	0.000328197	-3.483864974
nucleoside triphosphate metabolic process	0.000412505	-3.38457091
mitochondrial ATP synthesis coupled electron transport	0.000499088	-3.30182298
energy derivation by oxidation of organic compounds	0.001214622	-2.915559011

ATP metabolic process	0.001442191	-2.840977117
oxidative phosphorylation	0.001743473	-2.758584674
purine nucleoside monophosphate metabolic process	0.003038172	-2.517387689
purine ribonucleoside monophosphate metabolic process	0.003038172	-2.517387689
purine ribonucleotide metabolic process	0.003636962	-2.439261205
ribonucleoside monophosphate metabolic process	0.004003065	-2.39760731
nucleoside monophosphate metabolic process	0.004337275	-2.36278305
ribonucleotide metabolic process	0.004607968	-2.336490545
purine nucleotide metabolic process	0.004808753	-2.317967497
generation of precursor metabolites and energy	0.005452005	-2.263443725
ribose phosphate metabolic process	0.005523106	-2.257816589
purine-containing compound metabolic process	0.007539109	-2.12267996
macrophage differentiation	3.15817E-05	-4.500564205
myeloid leukocyte differentiation	6.21453E-05	-4.206591521
leukocyte differentiation	0.000952025	-3.021351681
myeloid cell development	0.002210671	-2.65547584
myeloid cell differentiation	0.007096324	-2.148966582
response to fungus	0.000164403	-3.784088951
actin filament bundle assembly	0.001102138	-2.957764099
actin filament bundle organization	0.001418793	-2.848081069
regulation of actin cytoskeleton organization	0.006860253	-2.163659852
regulation of actin filament-based process	0.007337717	-2.134439051
regulation of immune system process	0.002058806	-2.686384517
protein activation cascade	0.00259055	-2.586608026
humoral immune response	0.003834829	-2.416253972
phagosome	0.002652121	-2.576406594
phagocytosis	0.006036292	-2.219229771

Table S2-8: Enriched GO terms of MTZ-EC		
**Description**	***p* value**	**Log(*p* value)**
blood vessel development	1.09692E-10	-9.959823434
blood vessel morphogenesis	2.00595E-10	-9.697680451
vasculature development	9.67815E-10	-9.014207532
cardiovascular system development	9.67815E-10	-9.014207532
tube morphogenesis	1.32237E-08	-7.878645387
angiogenesis	2.0534E-08	-7.687527079
sprouting angiogenesis	7.55718E-08	-7.121640105
wound healing	4.02723E-08	-7.394993769
response to wounding	2.30617E-07	-6.637108087
tissue regeneration	0.000208466	-3.680964209
fin regeneration	0.000335099	-3.474826844
developmental growth	0.0007839	-3.105739525
regeneration	0.001258077	-2.900292637
growth	0.001298851	-2.886440693
head development	0.0069266	-2.159479891
central nervous system development	0.008941487	-2.048590267
Notch signaling pathway	4.90876E-06	-5.309028046
negative regulation of multicellular organismal process	3.09306E-05	-4.50961173
negative regulation of developmental process	0.000147958	-3.829862531
negative regulation of cell differentiation	0.000200862	-3.697101395
negative regulation of neurogenesis	0.003385957	-2.4703185
negative regulation of nervous system development	0.004113255	-2.385814315
negative regulation of cell development	0.00459175	-2.338021745
regulation of cell differentiation	0.0069266	-2.159479891
enzyme linked receptor protein signaling pathway	8.98329E-05	-4.046564632
transmembrane receptor protein tyrosine kinase signaling pathway	0.000454381	-3.342579736
peptidyl-tyrosine phosphorylation	0.005280206	-2.277349132
peptidyl-tyrosine modification	0.005835161	-2.233947156
vasculogenesis	9.07039E-05	-4.042374112
calcium-mediated signaling	0.000164111	-3.784863616
second-messenger-mediated signaling	0.000356923	-3.447425339
morphogenesis of a branching epithelium	0.00018415	-3.734828231
morphogenesis of a branching structure	0.000313488	-3.503778628
endothelial cell proliferation	0.000531952	-3.27412789
epithelial cell proliferation	0.002182658	-2.661014291
tissue morphogenesis	0.003146064	-2.502232402
regulation of cell-cell adhesion	0.005835161	-2.233947156
blood coagulation	0.000184949	-3.732947624
hemostasis	0.000196055	-3.707623048
coagulation	0.000219696	-3.658177309
regulation of body fluid levels	0.00042614	-3.370447389
platelet degranulation	0.000245325	-3.61025803
response to elevated platelet cytosolic Ca2+	0.000273037	-3.563778484
hemostasis	0.001827851	-2.738059278
platelet activation, signaling and aggregation	0.003778012	-2.42273669
artery development	0.000531952	-3.27412789

somitogenesis	0.001159597	-2.935692906
regionalization	0.001810126	-2.74229116
segmentation	0.001937242	-2.712816144
anterior/posterior pattern specification	0.004200914	-2.376656226
somite development	0.004351241	-2.361386901
pattern specification process	0.004813977	-2.317496004
embryonic cranial skeleton morphogenesis	0.005432424	-2.265006345
chordate embryonic development	0.007068188	-2.150691903
embryo development ending in birth or egg hatching	0.007211924	-2.141948864
embryonic skeletal system morphogenesis	0.007513436	-2.124161389
sensory system development	0.001211173	-2.916793834
mechanosensory lateral line system development	0.004758412	-2.322537937
lateral line development	0.009630636	-2.016345051
cell adhesion	0.002097934	-2.678208158
biological adhesion	0.002097934	-2.678208158
regulation of catalytic activity	0.003165953	-2.499495584
negative regulation of catalytic activity	0.007228517	-2.140950767
negative regulation of Wnt signaling pathway	0.006835245	-2.165245896
cell surface receptor signaling pathway involved in cell-cell signaling	0.007086056	-2.149595415
negative regulation of signal transduction	0.00734199	-2.134186226
negative regulation of cell communication	0.008822327	-2.054416872
negative regulation of signaling	0.008822327	-2.054416872

Table S2-9: Enriched GO terms of MTZ-EP		
**Description**	***p* value**	**Log(*p* value)**
ECM-receptor interaction	3.60867E-09	-8.442653103
focal adhesion	1.62904E-06	-5.788069437
extracellular matrix organization	0.000149443	-3.825523969
extracellular structure organization	0.000179912	-3.744940654
AGE-RAGE signaling pathway in diabetic complications	0.000262629	-3.580657291
skeletal system development	0.001467807	-2.833331163
response to wounding	4.63686E-07	-6.333775593
wound healing	9.19218E-07	-6.036581321
somite development	7.14625E-06	-5.145922022
somitogenesis	1.00875E-05	-4.996216334
segmentation	2.23652E-05	-4.650426824
regeneration	2.52308E-05	-4.598068668
tissue regeneration	2.56379E-05	-4.591118339
regionalization	0.000107876	-3.967074463
chordate embryonic development	0.000143326	-3.843673882
anterior/posterior pattern specification	0.000145722	-3.836475045
embryo development ending in birth or egg hatching	0.000147854	-3.830167235
pattern specification process	0.000528608	-3.27686651
developmental growth	0.00121093	-2.916881119
growth	0.00198871	-2.701428609
fin regeneration	0.005433406	-2.264927833
integrin cell surface interactions	5.42244E-07	-6.265805301
ECM proteoglycans	5.8546E-06	-5.23250254
degradation of the extracellular matrix	5.91329E-06	-5.228170717
extracellular matrix organization	3.19408E-05	-4.495653656
assembly of collagen fibrils and other multimeric structures	5.0933E-05	-4.293000698
collagen chain trimerization	5.0933E-05	-4.293000698
collagen degradation	8.72541E-05	-4.059214231
collagen biosynthesis and modifying enzymes	0.000512558	-3.290256658
signaling by PDGF	0.000558748	-3.252783781
collagen formation	0.000713032	-3.146890797
signaling by Receptor Tyrosine Kinases	0.005828844	-2.234417541
muscle structure development	0.006853415	-2.164092969
tube morphogenesis	1.52528E-06	-5.816649198
vasculature development	0.000191464	-3.717912494
cardiovascular system development	0.000191464	-3.717912494
blood vessel development	0.000296032	-3.528661788
blood vessel morphogenesis	0.000629031	-3.201328283
angiogenesis	0.004751162	-2.323200129
morphogenesis of an epithelium	2.10383E-06	-5.676988629
tissue morphogenesis	2.12899E-06	-5.671825709
embryonic morphogenesis	6.73692E-06	-5.171538507
epithelial tube formation	1.33434E-05	-4.874731914
tube formation	1.90914E-05	-4.719161995
epithelial tube morphogenesis	2.53175E-05	-4.596579095
embryonic eye morphogenesis	0.000104294	-3.981739147

embryonic organ development	0.000123247	-3.909222702
embryonic organ morphogenesis	0.000174839	-3.757362425
morphogenesis of embryonic epithelium	0.000217737	-3.662068465
sensory organ morphogenesis	0.000437728	-3.358795838
eye morphogenesis	0.000445927	-3.350735864
neural tube formation	0.000468891	-3.32892842
embryonic epithelial tube formation	0.000607519	-3.216440137
mesenchyme development	0.000624598	-3.204399598
neural tube development	0.001419363	-2.8479065
sensory organ development	0.004633407	-2.334099547
sensory system development	0.007260474	-2.139035008
camera-type eye morphogenesis	0.007638031	-2.11701859
mesenchymal cell differentiation	0.007796168	-2.10811883
specification of animal organ identity	2.02785E-05	-4.692963279
animal organ formation	0.000658928	-3.181161976
blood coagulation	0.003524748	-2.452871959
hemostasis	0.003677787	-2.43441347
coagulation	0.003995907	-2.398384592
protein activation cascade	0.004862897	-2.313104959
regulation of body fluid levels	0.006470416	-2.189067818
antimicrobial humoral response	5.0933E-05	-4.293000698
humoral immune response	0.000644697	-3.190644177
keratinization	0.000179238	-3.746570966
formation of the cornified envelope	0.000179238	-3.746570966
developmental biology	0.000457177	-3.339915307
morphogenesis of a polarized epithelium	0.000203001	-3.692501527
pectoral fin development	0.000217737	-3.662068465
fin development	0.001184794	-2.926357117
appendage development	0.001521656	-2.817683568
fin morphogenesis	0.002440521	-2.61251753
appendage morphogenesis	0.002951968	-2.529888282
cell migration	0.000417631	-3.379207556
cell motility	0.000792727	-3.100876552
localization of cell	0.000792727	-3.100876552
hemostasis	0.000474251	-3.323991924
cell surface interactions at the vascular wall	0.002095759	-2.67865867
platelet degranulation	0.004330294	-2.363482623
response to elevated platelet cytosolic Ca2+	0.004681187	-2.329643969
platelet activation, signaling and aggregation	0.004906411	-2.309236072
nephron development	0.000644697	-3.190644177
kidney development	0.001663367	-2.779011791
renal system development	0.001663367	-2.779011791
urogenital system development	0.001826905	-2.738284085
nephron epithelium development	0.001883814	-2.724961931
glomerulus development	0.002688722	-2.570454035
kidney epithelium development	0.00323055	-2.49072349
negative regulation of canonical Wnt signaling pathway	0.00256276	-2.591292127
negative regulation of response to stimulus	0.007426661	-2.129206389

negative regulation of Wnt signaling pathway	0.008360114	-2.077787798
dorsal/ventral pattern formation	0.005514866	-2.258465017
phagosome	0.005903141	-2.228916871
response to cytokine	0.009139383	-2.039083142

Table S2-10: Enriched GO terms of MTZ-EPDC		
**Description**	***p* value**	**Log(*p* value)**
extracellular matrix organization	4.79066E-09	-8.319605075
degradation of the extracellular matrix	9.73642E-05	-4.011600638
ECM proteoglycans	0.00018415	-3.734828231
platelet degranulation	0.000245325	-3.61025803
response to elevated platelet cytosolic Ca2+	0.000273037	-3.563778484
platelet activation, signaling and aggregation	0.000438427	-3.358102341
integrin cell surface interactions	0.000450858	-3.345959976
hemostasis	0.001827851	-2.738059278
signaling by receptor tyrosine kinases	0.004273197	-2.369247091
ECM-receptor interaction	1.29357E-06	-5.888208493
focal adhesion	8.72745E-06	-5.059112682
AGE-RAGE signaling pathway in diabetic complications	0.001937242	-2.712816144
hydrogen peroxide catabolic process	1.44402E-06	-5.840426655
hydrogen peroxide metabolic process	2.61349E-06	-5.58277929
drug catabolic process	5.91228E-06	-5.228245318
reactive oxygen species metabolic process	6.30958E-06	-5.199999875
antibiotic catabolic process	1.03066E-05	-4.986883261
cofactor catabolic process	2.81873E-05	-4.549946364
oxygen transport	5.92422E-05	-4.227368874
gas transport	0.00011043	-3.956911387
antibiotic metabolic process	0.000273037	-3.563778484
response to drug	0.002664149	-2.574441495
drug metabolic process	0.006513783	-2.186166708
drug transport	0.008878498	-2.051660511
wound healing	5.26461E-06	-5.278633392
fin regeneration	1.98628E-05	-4.701959275
response to wounding	2.04677E-05	-4.688930267
tissue regeneration	0.000208466	-3.680964209
regeneration	0.001258077	-2.900292637
heart field specification	6.2708E-06	-5.202677232
specification of animal organ identity	1.62716E-05	-4.788570016
heart formation	0.000284026	-3.546641396
animal organ formation	0.000531952	-3.27412789
cell-matrix adhesion	0.002397505	-2.620240457
blood coagulation	0.002865441	-2.542808512
hemostasis	0.002990569	-2.52424623
coagulation	0.003250799	-2.488009901
regulation of body fluid levels	0.005280206	-2.277349132
cell adhesion	0.007476493	-2.126302073
biological adhesion	0.007476493	-2.126302073
protein activation cascade	1.63576E-05	-4.78627936
humoral immune response	0.000488546	-3.311094982
complement activation	0.002625018	-2.580867764
extracellular matrix organization	9.94904E-05	-4.00221873
extracellular structure organization	0.000119972	-3.920919157
elastic fibre formation	0.000144242	-3.840906787

collagen formation	0.000575769	-3.239751635
skeletal system development	0.000166155	-3.779486901
cartilage development	0.004871664	-2.312322645
connective tissue development	0.005668327	-2.246545127
sensory organ morphogenesis	0.00889221	-2.050990274
muscle contraction	0.001562807	-2.80609468
muscle system process	0.001937242	-2.712816144
vasculature development	0.002210457	-2.655517874
cardiovascular system development	0.002210457	-2.655517874
blood vessel development	0.00384474	-2.415133054
tube morphogenesis	0.0066657	-2.176154229
blood vessel morphogenesis	0.008529685	-2.06906702
G alpha (i) signalling events	0.002366718	-2.625853397
signaling by GPCR	0.007170549	-2.144447583
inflammatory response	0.00971703	-2.012466462
vascular smooth muscle contraction	0.003778012	-2.42273669
heart contraction	0.004154054	-2.38152782
heart process	0.004554658	-2.341544219
