# Supplementary material for: Single-cell analysis reveals an Angpt4-initiated EPDC-EC-CM cellular coordination cascade during heart regeneration
Source: Protein Cell. 2022 May 18;14(5):350–68. doi: 10.1093/procel/pwac010 (PMC10166170; doi:10.1093/procel/pwac010)
Supplement: pwac010_suppl_Supplementary_Table_S3 [file pwac010_suppl_supplementary_table_s3.pdf]

Table S3: Primer sequence used in this study

Primer name	Sequence
aldh1a2 in situ F	GAGGCAAAGCTCCTGCTACT
aldh1a2 in situ R	ACTCGATTTCTACCCAGTGGA
angpt4 in situ F	TGTGGTCGAAGCCAAGGTTAT
angpt4 in situ R	CTGGGTCCTCGGAAGTGATG
ctsk in situ F	GTGTGGACTGGCTCACTCTC
ctsk in situ R	ACTTCTTGCCTCTCGGTGTG
fn1a in situ F	CCAGCACTCCCATCCATGTT
fn1a in situ R	GCCAGAAATGGCCTCAGTCT
aldh1a2 qpcr F	ACAAAGTGGCGTTCACAGGA
aldh1a2 qpcr R	CTCACCTGAGGTCCGTGTTC
angpt4 qpcr F	GCCAGTACTCCCTTCGAGTG
angpt4 qpcr R	CAAACCACCAGCCTCCAGTC
ctsk qpcr F	ACTGGGATACGTCACTTCGG
ctsk qpcr R	TGTTGTAGGCACACTGCTGA
grb2a qpcr F	ACAAGGCTGAACTCAACGGAA
grb2a qpcr R	TATTTTCCAGCTCCGTCCCG
grb2b qpcr F	TCCTCTGGTCCACAATAGGGAT
grb2b qpcr R	CCCGAAAAACCACGGATGTG
gapdh qpcr F	TGACTTCAATGGGGATTGCC
gapdh qpcr R	AAACAGCAAAGGGGTCACAT
kras qpcr F	AAAGCGCTCTCACCATCCAA
kras qpcr R	CCTCGGAGTCCTTTACTCGC
map2k2a qpcr F	GCGTATCTTCGGGAGAAGCA
map2k2a qpcr R	GCTCCACACATCCGACTGAA
map2k2b qpcr F	ATTCTGGTAAACTGTCGCGGG
map2k2b qpcr R	GTGCTGTGTGTTTCTGCCTCT
mras qpcr F	TCGAAACGGACTGTTGGCAT
mras qpcr R	CCGTCGATCTCAGTGTGCTT
nras qpcr F	GAGAGGGTTTCCTCTGCGTC
nras qpcr R	GGATCTCACGGACAAGGGTG
rras qpcr F	AGAGCAGTACATGCGCTCTG
rras qpcr R	GGATCCTGTTTTCTCGGGCA
rxrga qpcr F	AGGTTGGAACGAGCTGCTTA
rxrga qpcr R	GCTGATCCGGGTAGTTGTGT
sos1 qpcr F	CCCGATGGAGGACATGATGG
sos1 qpcr R	GATGCGGCTGTAGCTGATCT
sos2 qpcr F	CCGCTCTTTCTGCAAACCAC
sos2 qpcr R	TGATGTTCCACCCACTGACG
